# Design, Synthesis, and Biological Evaluation of *N*,*N*-Disubstituted-4-arylthiazole-2-methylamine Derivatives as Cholesteryl Ester Transfer Inhibitors

**DOI:** 10.3390/molecules22111925

**Published:** 2017-11-07

**Authors:** Xinran Wang, Xuehua Lin, Xuanqi Xu, Wei Li, Lijuan Hao, Chunchi Liu, Dongmei Zhao, Maosheng Cheng

**Affiliations:** 1 Key Laboratory of Structure-Based Drug Design & Discovery of Ministry of Education, Shenyang Pharmaceutical University, Shenyang 110016, China; wangxinrancai@163.com (X.W.); lin_xuehua1995@163.com (X.L.); 15542173071@163.com (W.L.); 18341482606@163.com (L.H.); liuchunchi1990@163.com (C.L.); mscheng@263.net (M.C.); 2 Department of Chemistry, University of Wisconsin-Madison, Madison, WI 53715, USA; xxu53@wisc.edu

**Keywords:** synthesis, *N*,*N*-disubstituted-4-arylthiazole-2-methylamine derivatives, CETP inhibitors

## Abstract

Cholesteryl ester transfer protein (CETP) has been identified as a potential target for cardiovascular disease (CVD) for its important role in the reverse cholesteryl transfer (RCT) process. In our previous work, compound **5** was discovered as a moderate CETP inhibitor. The replacement of the amide linker by heterocyclic aromatics and then a series of *N*,*N*-substituted-4-arylthiazole-2-methylamine derivatives were designed by utilizing a conformational restriction strategy. Thirty-six compounds were synthesized and evaluated for their CETP inhibitory activities. Structure-activity relationship studies indicate that electron donor groups substituted ring A, and electron-withdrawing groups at the 4-position of ring B were critical for potency. Among these compounds, compound **30** exhibited excellent CETP inhibitory activity (IC_50_ = 0.79 ± 0.02 μM) in vitro and showed an acceptable metabolic stability.

## 1. Introduction

Robust evidence suggests that a high level of low-density lipoprotein cholesterol (LDL-C) and a low level of high-density lipoprotein cholesterol (HDL-C) are closely associated with cardiovascular disease (CVD) [1,2,3,4]. Epidemiological studies have identified that each 1 mmol/L decrease of LDL-C reduces cardiovascular events by 22% [5]. In contrast to LDL-C, the risk of cardiovascular events will be reduced 2% to 3% for each 0.1 mg/dL increase in HDL-C [6]. Despite the successful utilization of statins in clinical treatment for reducing LDL-C levels, the residual risk of CVD events remains at high levels [7,8,9,10]. Plasma cholesteryl ester transfer protein (CETP), secreted mainly from the liver, plays a coordinating part in reverse cholesteryl transfer (RCT) that facilitates the transfer of triglyceride and cholesteryl ester (CE) between lipoproteins. The elevation of HDL-C via inhibiting CETP is an effective strategy for reducing the risk of cardiovascular events.

**Figure 1:**
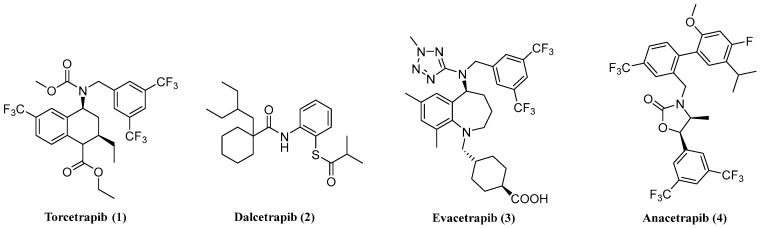
Representative cholesteryl ester transfer protein (CETP) inhibitors.

Up to now, several CETP inhibitors have been reported, and four of them have exhibited preeminent activity in phase III clinical trials (Figure 1) [11]. Torcetrapib was the first small molecule CETP inhibitor be appraised in the clinic. Early evidence has demonstrated that torcetrapib exhibited a dose-dependent increase of HDL-C greater than 100% and resulted in incremental LDL-C decreasing by up to 42% in human studies. However, torcetrapib was prematurely halted because of off-target hyperaldosteronism that lead to a 58% increase in deaths in the torcetrapib/atorvastatin group compared to the atorvastatin group [12,13,14]. Compared with torcetrapib, dalcetrapib showed modest CETP inhibition and no off-target effects. However, the phase III clinical trial was terminated due to the fact that dalcetrapib did not significantly decrease the risk of cardiovascular events [15,16]. Recently, DalCor Pharmaceuticals licensed dalcetrapib from Roche to conduct clinical trials for the treatment of acute coronary syndrome. The effect of evacetrapib on a reduction in CVD events was similar to trocetrapib, while avoiding torcetrapib’s side effects [17]. Subsequently, the ACCELERATE trials of evacetrapib were terminated after just over two years, but the reason for failure has not been announced by Lilly. Anacetrapib is currently ongoing in phase III trials at Merck and Co. (Kenilworth, NJ, USA) for the treatment of coronary artery disease. More recently, Merck announced that anacetrapib significantly reduced the incidence of major coronary events, while anacetrapib’s safety profile was generally consistent with that of previous studies of the drug. The phase II clinical trials demonstrated that AMG-899 seems to be free of the off-target effects of torcetrapib, effectively reduced LDL-C levels by 45.3%, and increased HDL-C levels by 179.1% [18].

**Figure 2:**
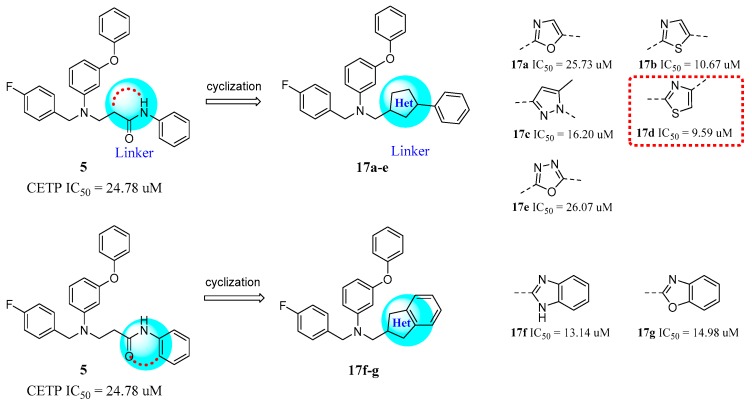
Design of *N*,*N*-substituted-aryl-methylamines.

In previous work, we found that compound **5** exhibited weak CETP inhibition activity. Based on the structure–activity relationship of compound **5**, we replaced amide fragments with different heterocyclic aromatics and benzoheteroaromatics to decrease molecular flexibility while keeping the key pharmacophores invariant. As shown in Figure 2, the replacement of amide linker with seven fragments revealed a slight increase in activity, and the replacement of compound **17d** with a 4-phenylthiazole side chain showed better CETP inhibition activities (IC_50_ = 9.03 ± 0.21 μM). Under the consideration of the structural novelty and the difficulty of synthesis, compound **17d** was selected as the leader for structure optimization, and a series of *N*,*N*-substituted-4-arylthiazole-2-methylamine derivatives were synthesized. Further optimization efforts in part A and part B led to the discovery of compound **30** (IC_50_ = 0.79 ± 0.02 μM).

## 2. Results and Discussion

### 2.1. Chemistry

**Scheme 1:**
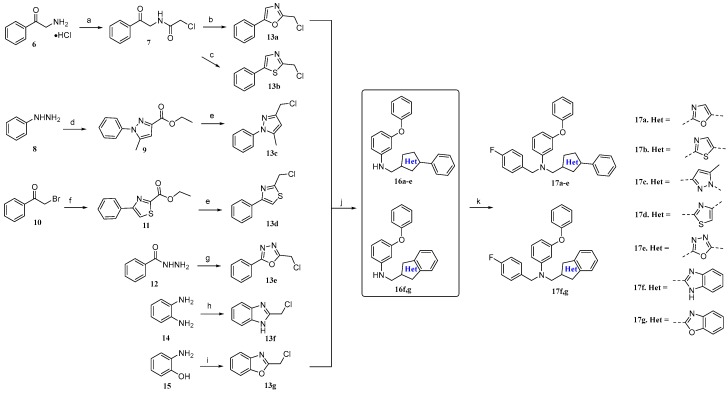
Synthesis of target compounds **17a**–**g**. Reagents and conditions: (**a**) 2-Chloroacetyl chloride, TEA, DCM, 0 °C, 62.9%; (**b**) POCl_3_, CH_3_CN, reflux, 66.2%; (**c**) Lawesson’s reagent, THF, reflux, 59.6%; (**d**) Ethyl acetopyruvate, EtOH, reflux, 43.6%; (**e**) (i) NaBH_4_, THF, r.t.; (ii) SOCl_2_, DMF, r.t., 75.9~80.5%; (**f**) Ethyl 2-amino-2-thioxoacetate, EtOH, reflux, 73.6%; (**g**) 2-Chloroacetic acid, POCl_3_, reflux, 81.1%; (**h**) Ethyl 2-chloroacetate, HCl, 110 °C, 91.3%; (**i**) 2-Chloroacetyl chloride, p-TsOH, pyridine, chlorobenzene, reflux, 90.2%; (**j**) 3-Phenoxyaniline, K_2_CO_3_, KI, DMF, r.t., 52.6~70.3%; (**k**) 1-(Bromomethyl)-4-fluorobenzene, K_2_CO_3_, KI, DMF, r.t., 64.9~71.6%.

Compounds **17a**–**g** were prepared according to Scheme 1 and ^1^H-NMR, ^13^C-NMR, HRMS for these target compounds are gave in supplementary materials. The commercially available 2-amino-1-phenylethan-1-one hydrochloride (**6**) reacted with 2-chloroacetyl chloride to give **7**. The resulting **7** underwent cyclization reactions, respectively, with POCl_3_ and Lawesson’s reagent to produce **13a** and **13b**. The treatment of **8** with ethyl acetopyruvate achieved **9** in moderate yield. Then **9** was reacted with NaBH_4_ and, subsequently, SOCl_2_ to afford **13c**. The key intermediate **11** was prepared by **10** with ethyl 2-amino-2-thioxoacetate in ethanol under reflux conditions. In the next step, **11** was treated with the same operation as **9** to obtain **13d**. Compounds **13e**–**f** were respectively prepared from the cyclization of **12**, **14**, and **15** with different derivatives of chloroacetic acid. Compounds **16a**–**g** were obtained by the nucleophilic substitution of **13a**–**g** and 3-phenoxyaniline in the presence of K_2_CO_3_ and KI in DMF. The secondary amines **16a**–**g** were treated with 1-(bromomethyl)-4-fluorobenzene to generate target compounds **17a**–**g**.

**Scheme 2:**
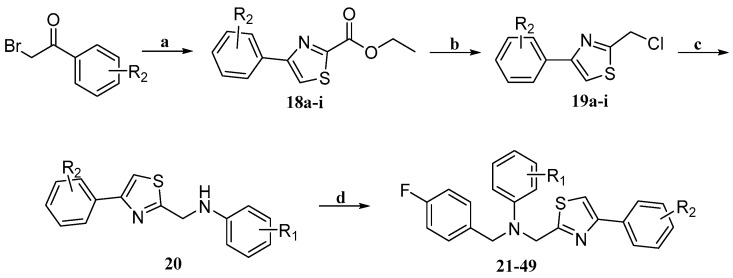
Synthesis of target compounds **21** to **49**. Reagents and conditions: (**a**) Ethyl 2-amino-2-thioxoacetate, EtOH, reflux, 70.6~79.1%; (**b**) (**i**) NaBH_4_, THF, r.t.; (**ii**) SOCl_2_, DMF, r.t., 75.9~80.5%; (**c**) Corresponding anilines, K_2_CO_3_, KI, DMF, r.t., 52.6~70.3%; (**d**) 1-(Bromomethyl)-4-fluorobenzene, K_2_CO_3_, KI, DMF, r.t., 64.9~71.6%.

The synthesis of compounds **21**–**49** was outlined in Scheme 2 and ^1^H-NMR, ^13^C-NMR, HRMS for these target compounds are gave in supplementary materials. The intermediate **18** was prepared by the cyclization of 2-bromo-1-phenylethan-1-one with ethyl 2-amino-2-thioxoacetate in ethanol under reflux conditions. Then the ester function in **18** was reduced with NaBH_4_ and reacted subsequently with SOCl_2_ to afford **19**. Compound **20** was obtained by the nucleophilic substitution of **19** and 3-phenoxyaniline in the presence of K_2_CO_3_ and KI in DMF. Intermediate **20** and 1-(bromomethyl)-4-fluorobenzene underwent a nucleophilic reaction to get target compounds **21** to **49**.

### 2.2. In Vitro Activity and Structure–Activity Relationships

The biological activity of *N*,*N*-disubstituted-aryl-methylamine derivatives and reference compound anacetrapib (**4**) against CETP was evaluated by a BODIPY-CE fluorescence assay with the CETP RP Activity Assay Kit (catalog # RB-RPAK; Roar, New York, NY, USA). The results showed that most of the target compounds exhibit potent CETP inhibitory activity.

As shown in Table 1, 4-phenylthiazole (**17d**) revealed better activity than other links. Further optimization based on *N*,*N*-substituted-4-arylthiazole-2-methylamine scaffolding was underway, and in vitro activity against CETP is shown in Table 2. We investigated the relationship between various groups at the 2-position, 3-position, and 4-position of the benzene (Ring A) and the CETP inhibitory activity. The introduction of the 3,4-dimethoxy group (**22**, **30**–**33**) was beneficial to activity. The replacement of hydrogen by trifluoromethoxy (**23**) and *N*-methyl-5-yl-pyrazole (**29**) at the 3-position was detrimental to activity. Changing the 3-H group to 3-OCH_3_ (**21**) and 3-CF_3_ (**24**) slightly decreased CETP inhibition activity. Thus, we could conclude that electron donor groups, in particular the 3,4-dimethoxy group, substituted in ring A were conducive to activity. Next, the effect of the aromatic ring on activity was investigated; then phenyl, 2-thienyl and *N*-methyl-5-yl-pyrazolyl were induced to modify ring A. Specifically, it was observed that the activity of compound **27** (IC_50_ = 1.02 ± 0.01 μM) was a seven-fold improvement over compound **26** (IC_50_ = 8.98 ± 0.05 μM) and an eight-fold improvement over compound **25** (IC_50_ = 9.05 ± 0.08 μM). Compared to the phenyl-substituted ring A at the 3-position (**26**), 2-thienyl (**28**) and *N*-methyl-5-yl-pyrazolyl (**29**) showed no advantage. The results from the modification of the ring A moiety indicated that phenyl-substituted compounds were favorable for activity compared to thienyl and pyrazolyl, and the position of the phenyl group provided an important contribution to the inhibitory activity. However, the potency of compound **30** (IC_50_ = 0.79 ± 0.02 μM) was superior to compound **27**, and, for that reason, the (3,4-dimethoxyl) phenyl fragment was chosen for further study of the relationship between ring B and the CETP inhibitory activity. For this purpose, another nineteen compounds, **31** to **49**, were synthesized and evaluated for their activities. Compounds containing electron withdrawing groups (**31** to **33**) at the 4-position of ring B revealed significant improvement of activity compared to those compounds with electron donor groups (**42** to **45**). The nitro group at the 4-position of ring B (**30**) exhibited better potency of CETP inhibitory activity than that substituted at the 2-position (**35**) and the 3-position (**34**). The introduction of bromine (**36**), 1-methylpyrazole (**37**, **39**), isoxazole (**38**), thiazole (**40**), and benzene (**41**) was tolerated, but 4-morpholinyl (**49**) led to a disappearance of activity. Replacing the nitro group on the 4-position of ring B with an ester side chain (**48**) caused nearly a 10-fold decrease of activity. Changing the 4-NH_2_ group (**44**) to a 4-NHCOCH_3_ group (**46**) showed a dramatic decrease in the activity; however, potency was recovered when modified by a 4-NHCOCF_3_ group (**45**) was introduced. Based on these in vitro structure-activity relationshipstudies, we could speculate that electron-withdrawing groups at the 4-position of ring B provide an important contribution to the potency compared with electron withdrawing groups and bulkier rigid fragments.

### 2.3. In Vitro Metabolic Stability Study

Based on the result of the in vitro CETP inhibitory assay, potent inhibitors **30** and **32** were selected for the in vitro metabolic stability study. As shown in Table 3, compound **30** showed weak stability, with a clearance rate of 48.1 and 121.7 μL/min/mg in human and rat liver microsomes, while compound **32** exhibited acceptable stability, with a clearance rate of 29.8 and 79.3 μL/min/mg in human and rat liver microsomes.

## 3. Experimental

### 3.1. Chemicals and Instruments

All chemicals and reagents were obtained from commercial sources and were used without purification. TLC, performed on silica gel plates (Indicator F-254), was used to monitor the reactions. Column chromatography, performed on silica gel (200 to 300 mesh) was utilized to purify the compounds. The melting points (uncorrected) were determined on a Buchi 353 melting-point apparatus. The purities of the target compounds were detected by HPLC, performed on a Waters 1525-2489 (Waters, Milford, MA, USA), with a chromatographic column (Kromasil, C-18, 5 μm, 150 mm × 4.6 mm), at ≥95%. The method conditions were as follows: a mixture of solvents H_2_O (A) and CH_3_CN (B) (VA:VB = 5:95) as eluent and a flow rate of 1.0 mL/min. Peaks were detected at *λ* = 254 nm. NMR spectra were collected on Bruker (Billerica, MA, USA) 400 MHz and 600 MHz instruments, using tetramethylchlorosilane as an internal standard and CDCl_3_ or DMSO-*d*_6_ as solvent. ESI-HRMS spectra were obtained on a Bruker Micromass time of flight mass spectrometer.

### 3.2. Synthesis

*2-Chloro-N-(2-oxo-2-phenylethyl)acetamide* (**7**). 2-amino-1-phenylethan-1-one hydrochloride **6** (0.50 g, 2.9 mmol) was dissolved in dichloromethane (10 mL), and triethylamine (13 mL, 8.9 mmol) was added. The mixture was cooled, and 2-chloroacetyl chloride (0.45 g, 4.0 mmol) was added in drops at 0 °C. After 2 h, the solution was recovered to room temperature for 16 h. Then the mixture was poured into water (20 mL) and extracted with dichloromethane (10 mL × 3), and the combined organic layers were washed with water (10 mL × 3) and brine (10 mL × 3), dried over Na_2_SO_4_, and concentrated in vacuo. The residue was purified by chromatography on silica gel (petroleum ether:ethyl acetate = 4:1) to give **7** (0.39 g, 62.9%) as a white solid.

*2-(Chloromethyl)-5-phenyloxazole* (**13a**). Intermediate **7** (0.20 g, 1 mmol) was dissolved in acetonitrile (10 mL), and phosphorus oxychloride (0.17 mL, 1.9 mmol) was added in a slow stream. The solution was heated at reflux for 4 h and then cooled to room temperature and concentrated. Ethyl acetate (20 mL) was added, washed with water and brine, dried over Na_2_SO_4_, and concentrated in vacuo. The residue was purified by chromatography on silica gel (petroleum ether:ethyl acetate = 20:1) to give **13a** (0.13 g, 66.2%) as a white solid.

*2-(Chloromethyl)-5-phenylthiazole* (**13b**). Intermediate **7** (0.30 g, 1.4 mmol) was dissolved in tetrahydrofuran (5 mL), and Lawesson’s reagent (0.34 g, 0.80 mmol) was added. The solution was heated at reflux for 4 h and then cooled to room temperature. The solvent was removed under reduced pressure, and the resulting residue was dissolved in ethyl acetate. The solution was washed with water and brine, dried over Na_2_SO_4_, and concentrated in vacuo. The residue was purified by chromatography on silica gel (petroleum ether:ethyl acetate = 10:1) to give **13b** (0.10 g, 59.6%) as a white solid.

*Ethyl 5-Methyl-1-phenyl-1H-pyrazole-3-carboxylate* (**9**). Phenylhydrazine (0.50 g, 5.0 mmol) and ethyl acetopyruvate (1.1 g, 7.0 mmol) were dissolved in ethanol (10 mL). After being heated at reflux for 2 h, the reaction mixture was cooled to room temperature and concentrated in vacuo. The residue was dissolved in ethyl acetate, washed with water and brine, dried over Na_2_SO_4_, and concentrated in vacuo. The residue was purified by chromatography on silica gel (petroleum ether:ethyl acetate = 10:1) to give **9** (0.50 g, 43.6%) as a white solid.

*3-(Chloromethyl)-5-methyl-1-phenyl-1H-pyrazole* (**13c**). In a solution of intermediate **9** (92.1 mg, 0.40 mmol) dissolved in ethanol (5 mL), sodium borohydride (19.0 mg, 0.50 mmol) was added. The mixture was stirred at room temperature for 30 min, and then water was added. The solution was extracted with ethyl acetate, and the combined organic layers were washed with water and brine, dried over Na_2_SO_4_, and concentrated in vacuo. The residue was dissolved in DMF (2 mL); then thionyl chloride (0.10 mL, 1.4 mmol) was added. After reflux for 1 h, water was added and extracted with ethyl acetate. The combined organic layers were washed with water and brine, dried over Na_2_SO_4_, and concentrated in vacuo. The residue was purified by chromatography on silica gel (petroleum ether:ethyl acetate = 10:1) to give **13c** (66.6 mg, 80.5%) as a white solid.

*Ethyl 4-Phenylthiazole-2-carboxylate* (**11**). A mixture of 2-bromo-1-phenylethan-1-one (0.50 g, 2.5 mmol) and ethyl 2-amino-2-thioxoacetate (0.50 g, 3.8 mmol) was dissolved in ethanol (10 mL). The solution was heated at reflux for 6 h and then cooled to room temperature. After being concentrated, the residue was dissolved in ethyl acetate (20 mL); then the solution was washed with water and brine, dried over Na_2_SO_4_, and concentrated in vacuo. The residue was purified by chromatography on silica gel (petroleum ether:ethyl acetate = 20:1) to give **11** (0.43 g, 73.6%) as a white solid.

*2-(Chloromethyl)-4-phenylthiazole* (**13d**). The ester function in **11** was reduced with NaBH_4_ and reacted subsequently with SOCl_2_ to afford **13d**, and this operation was the same as the step in the synthetic intermediate **13c**. Compound **13d** was obtained as a white solid. Yield: 75.9%.

*2-(Chloromethyl)-5-phenyl-1,3,4-oxadiazole* (**13e**). A mixture of benzohydrazide (0.50 g, 3.7 mmol), 2-chloroacetic acid (0.35 g, 3.7 mmol), and phosphorus oxychloride (1.0 mL, 11.0 mmol) was added to a three-necked round bottom flask. The solution was heated at reflux for 6 h and then cooled to 0 °C and neutralized to pH 9 with a saturated sodium carbonate aqueous solution. The precipitate was filtered, washed with water, and dried under an infrared lamp. Compound **13e** (0.58 g, 81.1%) was obtained as a white solid.

*2-(Chloromethyl)-1H-benzo[d]imidazole* (**13f**). A mixture of benzene-1,2-diamine (2.0 g, 18.0 mmol) and ethyl 2-chloroacetate, (2.6 mL, 24.0 mmol) was dissolved in dilute hydrochloric acid solution (4 mol/L, 16 mL). The solution was heated at 110 °C for 4 h and then cooled to room temperature. The reaction solution was poured into ice water and then neutralized to pH 9 with ammonium hydroxide. The precipitate was filtered, washed with water, and dried under an infrared lamp to obtain compound **13f** (2.7 g, 91.3%) was obtained as a white solid.

*2-(Chloromethyl)benzo[d]oxazole* (**13g**). In a solution of 2-aminophenol (0.50 g, 4.6 mmol) dissolved in chlorobenzene (5 mL), 2-chloroacetyl chloride (0.52 g, 4.6 mmol) and pyridine (0.02 mL) were added. The mixture was stirred at room temperature for 2 h; then *p*-toluene sulfonic acid (0.08 g, 0.46 mmol) was added. The mixture was heated at reflux for 8 h and then cooled to room temperature. The solvent was removed under reduced pressure, and the resulting residue was dissolved in ethyl acetate (30 mL). The solution was washed with water and brine, dried over Na_2_SO_4_, and concentrated in vacuo. The residue was purified by chromatography on silica gel (petroleum ether:ethyl acetate = 10:1) to give **13g** (0.70 g, 90.2%) as a yellow oil.

*3-Phenoxy-N-((5-phenyloxazol-2-yl)methyl)aniline* (**16a**). A mixture of 3-phenoxyaniline (0.20 g, 18.0 mmol) and **13a** (0.20 g, 18.0 mmol) was dissolved in DMF (10 mL), followed by the addition of potassium carbonate (0.90 g, 6.48 mmol) and KI. The solution was stirred at room temperature for 12 h and then poured into water and extracted with ethyl acetate. The combined organic layers were washed with water and brine, dried over Na_2_SO_4_, and concentrated in vacuo. The residue was purified by chromatography on silica gel (petroleum ether:ethyl acetate = 4:1) to give **16a** (0.26 g, 70.3%) as a colourless oil.

*N-(4-Fluorobenzyl)-3-phenoxy-N-((5-phenyloxazol-2-yl)methyl)aniline* (**17a**). Compound **16a** (0.20 g, 0.60 mmol) was added to a solution of (Bromomethyl)-4-fluorobenzene (0.08 mL, 0.60 mmol) in DMF (5 mL), followed by the addition of potassium carbonate (0.50 g, 3.6 mmol) and KI. The solution was stirred at room temperature for 24 h and then poured into water and extracted with ethyl acetate. The combined organic layers were washed with water and brine, dried over Na_2_SO_4_, and concentrated in vacuo. The residue was purified by chromatography on silica gel (petroleum ether:ethyl acetate = 4:1) to give **17a** (0.18 g, 66.9%) as a yellow solid. m.p. 81.0–82.4 °C. ^1^H-NMR (400 MHz, DMSO-*d*_6_) δ: 7.65~7.60 (m, 3H), 7.46 (t, *J* = 7.6 Hz, 2H), 7.38~7.27 (m, 5H), 7.18~7.11 (m, 3H), 7.06 (t, *J* = 7.4 Hz, 1H), 6.90 (d, *J* = 7.7 Hz, 2H), 6.61 (dd, *J* = 8.3, 2.2 Hz, 1H), 6.46 (t, *J* = 2.2 Hz, 1H), 6.26 (dd, *J* = 7.9, 1.8 Hz, 1H), 4.84 (s, 2H), 4.72 (s, 2H). ^13^C-NMR (150 MHz, DMSO-*d*_6_) δ: 161.65(d, *J* = 240 Hz), 161.50, 157.82, 157.03, 151.18, 149.93, 135.13, 135.11, 130.67(×2), 130.26(×2), 129.55, 129.02, 128.97, 127.87, 124.26(×2), 123.52, 122.98, 118.77(×2), 115.75, 115.61, 108.70, 107.51, 104.09, 54.71, 48.70. HRMS calcd. for C_29_H_23_FN_2_O_2_, [M + Na]^+^, 473.1641; found 473.1684. HPLC: *t*_R_ = 11.84 min, 97.50%.

*N-(4-Fluorobenzyl)-3-phenoxy-N-((5-phenylthiazol-2-yl)methyl)aniline* (**17b**). Yellow solid, yield: 84.0%. m.p. 90.5–92.5 °C. ^1^H-NMR (400 MHz, DMSO-*d*_6_) δ: 8.13 (s, 1H), 7.65~7.60 (m, 2H), 7.43 (t, *J* = 7.5 Hz, 2H), 7.37~7.26 (m, 5H), 7.19~7.11 (m, 3H), 7.07 (t, *J* = 7.4 Hz, 1H), 6.91~6.86 (m, 2H), 6.59 (dd, *J* = 8.4, 2.2 Hz, 1H), 6.41 (t, *J* = 2.2 Hz, 1H), 6.27 (dd, *J* = 7.9, 1.9 Hz, 1H), 4.96 (s, 2H), 4.74 (s, 2H). ^13^C-NMR (150 MHz, DMSO-*d*_6_) δ: 169.45, 161.69 (d, *J* = 240 Hz), 157.88, 156.93, 149.50, 138.98, 138.90, 134.92, 131.32, 130.77, 130.25(×2), 129.73(×2), 129.18, 129.13, 128.76, 126.76(×2), 123.57, 118.84(×2), 115.82, 115.68, 108.94, 107.62, 104.32, 54.47, 53.54. HRMS calcd. for C_29_H_23_FN_2_OS, [M + Na]^+^, 489.1413; found 489.1462. HPLC: *t*_R_ = 16.10 min, 95.77%.

*N-(4-Fluorobenzyl)-N-((5-methyl-1-phenyl-1H-pyrazol-3-yl)methyl)-3-phenoxyaniline* (**17c**). Yellow oil, yield: 64.9%. ^1^H-NMR (400 MHz, DMSO-*d*_6_) δ: 7.49~7.43 (m, 4H), 7.41~7.36 (m, 1H), 7.34~7.29 (m, 2H), 7.19~7.15 (m, 2H), 7.12~7.06 (m, 4H), 6.90~6.86 (m, 2H), 6.43~6.39 (m, 1H), 6.24~6.20 (m, 2H), 6.00 (s, 1H), 4.69 (s, 2H), 4.51 (s, 2H), 2.18 (s, 3H). ^13^C-NMR (150 MHz, DMSO-*d*_6_) δ: 160.96 (d, *J* = 240 Hz), 157.16, 156.37, 149.08, 147.95, 140.64, 139.21, 134.39, 130.02, 129.64(×2), 128.99(×2), 128.27, 128.22, 127.27, 124.17(×2), 122.90, 118.14(×2), 115.13, 114.99, 107.92, 106.47, 106.02, 103.20, 53.23, 47.01, 13.15. HRMS calcd. for C_30_H_26_FN_3_O, [M + H]^+^, 464.2138; found 464.2176. HPLC: *t*_R_ = 10.74 min, 97.05%.

*N-(4-Fluorobenzyl)-3-phenoxy-N-((4-phenylthiazol-2-yl)methyl)aniline* (**17d**). Colourless oil, yield: 70.3%. ^1^H-NMR (400 MHz, DMSO-*d*_6_) δ: 8.02 (s, 1H), 7.95~7.91 (m, 2H), 7.44 (t, *J* = 7.5 Hz, 2H), 7.37~7.31 (m, 3H), 7.29~7.25 (m, 2H), 7.19~7.11 (m, 3H), 7.03 (t, *J* = 7.4 Hz, 1H), 6.90~6.86 (m, 2H), 6.58 (dd, *J* = 8.3, 2.2 Hz, 1H), 6.42 (t, *J* = 2.2 Hz, 1H), 6.26 (dd, *J* = 8.0, 2.0 Hz, 1H), 5.02 (s, 2H), 4.75 (s, 2H). ^13^C-NMR (150 MHz, DMSO-*d*_6_) δ: 170.52, 161.68 (d, *J* = 241 Hz), 157.88, 156.91, 154.70, 149.51, 134.97, 134.95, 134.54, 130.75, 130.25(×2), 129.25(×2), 129.21, 129.16, 128.47, 126.42(×2), 123.55, 118.81(×2), 115.80, 115.65, 114.58, 108.90, 107.62, 104.27, 54.49, 53.63. HRMS calcd. for C_29_H_23_FN_2_OS, [M + Na]^+^, 489.1413; found 489.1467. HPLC: *t*_R_ = 17.55 min, 98.71%.

*N-(4-Fluorobenzyl)-3-phenoxy-N-((5-phenyl-1,3,4-oxadiazol-2-yl)methyl)aniline* (**17e**). White solid, yield: 64.9%. m.p. 97.3–98.2 °C. ^1^H-NMR (400 MHz, CDCl_3_) δ: 7.99 (d, *J* = 7.0 Hz, 2H), 7.58~7.48 (m, 3H), 7.32~7.26 (m, 4H), 7.21 (t, *J* = 8.2 Hz, 1H), 7.11~6.97 (m, 5H), 6.71 (dd, *J* = 8.3, 2.0 Hz, 1H), 6.60 (s, 1H), 6.47 (d, *J* = 8.0 Hz, 1H), 4.78 (s, 2H), 4.67 (s, 2H). ^13^C-NMR (150 MHz, DMSO-*d*_6_) δ: 164.72, 164.67, 161.68 (d, *J* = 241 Hz), 157.88, 157.03, 149.70, 134.90, 134.88, 132.53, 130.77, 130.27(×2), 129.93, 129.07, 129.01, 126.91(×2), 123.68, 123.56, 118.78(×2), 115.78, 115.64, 108.88, 107.87, 104.33, 54.49, 49.07. HRMS calcd. for C_28_H_22_FN_3_O_2_, [M + Na]^+^, 474.1594; found 474.1638. HPLC: *t*_R_ = 8.75 min, 96.33%.

*N-((1H-Benzo[d]imidazol-2-yl)methyl)-N-(4-fluorobenzyl)-3-phenoxyaniline* (**17f**). Yellow oil, yield: 57.3%. ^1^H-NMR (600 MHz, DMSO-*d*_6_) δ: 7.65~7.60 (m, 1H), 7.42~7.39 (m, 1H), 7.31 (t, *J* = 7.9 Hz, 2H), 7.20~7.15 (m, 4H), 7.12~7.06 (m, 3H), 7.04 (t, *J* = 8.1 Hz, 1H), 6.93 (d, *J* = 7.8 Hz, 2H), 6.52~6.45 (m, 2H), 6.36 (t, *J* = 2.1 Hz, 1H), 6.16 (dd, *J* = 7.9, 2.0 Hz, 1H), 5.55 (s, 2H), 4.52 (d, *J* = 5.1 Hz, 2H). ^13^C-NMR (150 MHz, DMSO-*d*_6_) δ: 161.90 (d, *J* = 241 Hz), 157.88, 157.29, 152.73, 150.39, 142.43, 135.93, 133.48, 130.52, 130.26(×2), 129.34, 129.28, 123.44, 122.81, 122.16, 119.42, 118.83(×2), 115.97, 115.83, 110.90, 108.31, 106.99, 103.35, 46.16, 41.14. HRMS calcd. for C_27_H_22_FN_3_O, [M + H]^+^, 424.1825; found 424.1859. HPLC: *t*_R_ = 5.72 min, 97.98%.

*N-(Benzo[d]oxazol-2-ylmethyl)-N-(4-fluorobenzyl)-3-phenoxyaniline* (**17g**). Colourless oil, yield: 71.0%. ^1^H-NMR (400 MHz, DMSO-*d*_6_) δ: 7.74~7.67 (m, 2H), 7.40~7.32 (m, 4H), 7.27~7.21 (m, 2H), 7.17~7.03 (m, 4H), 6.89~6.85 (m, 2H), 6.58 (dd, *J* = 8.3, 2.2 Hz, 1H), 6.41 (t, *J* = 2.2 Hz, 1H), 6.24 (dd, *J* = 8.0, 1.9 Hz, 1H), 5.01 (s, 2H), 4.76 (s, 2H). ^13^C-NMR (150 MHz, DMSO-*d*_6_) δ: 164.45, 161.66 (d, *J* = 241 Hz), 157.89, 156.89, 150.76, 149.81, 141.04, 135.06, 130.72, 130.21(×2), 128.95, 128.90, 125.59, 124.96, 123.56, 120.10, 118.83(×2), 115.78, 115.64, 111.28, 108.50, 107.39, 103.89, 54.81, 48.96. HRMS calcd. for C_27_H_21_FN_2_O_2_, [M + H]^+^, 425.1665; found 425.1725. HPLC: *t*_R_ = 18.55 min, 96.41%.

### 3.3. General Procedure for the Synthesis of Compounds ***21**–**49***

A mixture of substituted 2-bromo-1-phenylethan-1-one (2.5 mmol) and ethyl 2-amino-2-thioxoacetate (3.8 mmol) was dissolved in ethanol (10 mL). The solution was heated at reflux for 6 h and then cooled to room temperature. After being concentrated, the residue was dissolved in ethyl acetate (20 mL); then the solution was washed with water and brine, dried over Na_2_SO_4_, and concentrated in vacuo. The residue was purified by chromatography on silica gel to give **18**. The intermediate **19** was obtained by an operation similar to that described for the preparation of **13c**. Subsequently, substituted arylamine reacted with **19** according to the synthesis condition of compound **16a** to produce **20**. Finally, target compounds **21** to **49** were prepared according to the procedure for **17a**, except that different substrates were used.

*N-(4-Fluorobenzyl)-3-methoxy-N-((4-phenylthiazol-2-yl)methyl)aniline* (**21**). Colourless oil, yield: 64.1%. ^1^H-NMR (400 MHz, DMSO-*d*_6_) δ: 8.00 (s, 1H), 7.98~7.94 (m, 2H), 7.45 (t, *J* = 7.6 Hz, 2H), 7.39~7.32 (m, 3H), 7.17 (t, *J* = 12.3 Hz, 2H), 7.04 (t, *J* = 8.2 Hz, 1H), 6.39 (dd, *J* = 8.3, 2.2 Hz, 1H), 6.31 (t, *J* = 2.2 Hz, 1H), 6.27 (dd, *J* = 8.1, 2.1 Hz, 1H), 5.01 (s, 2H), 4.76 (s, 2H), 3.62 (s, 3H). ^13^C-NMR (150 MHz, DMSO-*d*_6_) δ: 171.03, 162.47~160.87 (d, *J* = 240 Hz), 160.66, 154.60, 149.19, 135.30, 134.56, 130.30, 129.28(×3), 129.22, 128.47, 126.40(×2), 115.76, 115.62, 114.61, 106.54, 102.82, 100.12, 55.18, 54.44, 53.62. HRMS calcd. for C_24_H_21_FN_2_OS, [M + H]^+^, 405.1437; found 405.1480. HPLC: *t*_R_ = 8.71 min, 95.81%.

*N-(4-Fluorobenzyl)-3,4-dimethoxy-N-((4-phenylthiazol-2-yl)methyl)aniline* (**22**). Colourless oil, yield: 65.6%. ^1^H-NMR (600 MHz, DMSO-*d*_6_) δ: 7.99 (s, 1H), 7.96~7.92 (m, 2H), 7.44 (t, *J* = 7.7 Hz, 2H), 7.40~7.36 (m, 2H), 7.34 (t, *J* = 7.4 Hz, 1H), 7.16 (t, *J* = 8.9 Hz, 2H), 6.74 (d, *J* = 8.8 Hz, 1H), 6.56 (d, *J* = 2.8 Hz, 1H), 6.27 (dd, *J* = 8.8, 2.8 Hz, 1H), 4.90 (s, 2H), 4.65 (s, 2H), 3.62 (d, *J* = 6.6 Hz, 6H). ^13^C-NMR (150 MHz, DMSO-*d*_6_) δ: 171.25, 161.67 (d, *J* = 240 Hz), 154.40, 149.86, 142.94, 142.04, 135.47, 134.59, 130.06, 129.69, 129.28(×2), 129.45, 126.37(×2), 115.67, 115.53, 114.67, 113.96, 106.28, 101.19, 56.54, 55.79, 55.27, 54.19. HRMS calcd. for C_25_H_23_FN_2_O_2_S, [M + Na]^+^, 457.1362; found 457.1409. HPLC: *t*_R_ = 7.11 min, 98.24%.

*N-(4-Fluorobenzyl)-N-((4-(4-nitrophenyl)thiazol-2-yl)methyl)-3-(trifluoromethoxy)aniline* (**23**). Colourless oil, yield: 54.9%. ^1^H-NMR (600 MHz, DMSO-*d*_6_) δ: 8.39 (s, 1H), 8.31 (d, *J* = 9.0 Hz, 2H), 8.22 (d, *J* = 9.0 Hz, 2H), 7.40~7.35 (m, 2H), 7.24 (t, *J* = 8.3 Hz, 1H), 7.21~7.16 (m, 2H), 6.81 (dd, *J* = 8.5, 2.4 Hz, 1H), 6.72 (s, 1H), 6.61 (d, *J* = 8.1 Hz, 1H), 5.11 (s, 2H), 4.82 (s, 2H). ^13^C-NMR (150 MHz, DMSO-*d*_6_) δ: 171.07, 161.76 (d, *J* = 241 Hz), 152.47, 149.92, 149.43, 147.14, 140.39, 134.54, 131.01, 129.30, 129.25, 127.31(×2), 124.75(×2), 119.15, 115.87, 115.73, 112.34, 109.27, 105.94, 62.92, 54.30, 53.32. HRMS calcd. for C_24_H_17_F_4_N_3_O_3_S, [M + H]^+^, 504.1005; found 504.1063. HPLC: *t*_R_ = 12.63 min, 95.83%.

*N-(4-Fluorobenzyl)-N-((4-(4-nitrophenyl)thiazol-2-yl)methyl)-3-(trifluoromethyl)aniline* (**24**). Brown solid, yield: 65.9%. m.p. 107.0–108.4 °C. ^1^H-NMR (600 MHz, CDCl_3_) δ: 8.31~8.27 (m, 2H), 8.06~8.03 (m, 2H), 7.61 (s, 1H), 7.31 (t, *J* = 8.0 Hz, 1H), 7.26 (d, *J* = 7.2 Hz, 2H), 7.14 (s, 1H), 7.07~7.03 (m, 3H), 6.97 (dd, *J* = 8.4, 2.6 Hz, 1H), 4.92 (s, 2H), 4.73 (s, 2H). ^13^C-NMR (150 MHz, DMSO-*d*_6_) δ: 170.91, 161.76 (d, *J* = 241 Hz), 152.48, 148.16, 147.15, 140.37, 134.49, 130.57, 129.27, 129.23, 127.30(×2), 125.71, 124.75(×2), 123.91, 119.19, 117.13, 115.91, 115.77, 113.85, 109.41, 54.26, 53.21. HRMS calcd. for C_24_H_17_F_4_N_3_O_2_S, [M + H]^+^, 488.1056; found 488.1104. HPLC: *t*_R_ = 9.95 min, 96.05%.

*N-(4-Fluorobenzyl)-N-((4-(4-nitrophenyl)thiazol-2-yl)methyl)-[1,1′-biphenyl]-4-amine* (**25**). Light yellow solid, yield: 54.9%. m.p. 164.5–166.2 °C. ^1^H-NMR (600 MHz, DMSO-*d*_6_) δ: 8.38 (s, 1H), 8.34~8.30 (m, 2H), 8.25~8.22 (m, 2H), 7.54 (dd, *J* = 8.3, 1.1 Hz, 2H), 7.48 (d, *J* = 8.9 Hz, 2H), 7.41~7.35 (m, 4H), 7.23 (dt, *J* = 8.5, 1.1 Hz, 1H), 7.21~7.16 (m, 2H), 6.88 (d, *J* = 9.0 Hz, 2H), 5.10 (s, 2H), 4.84 (s, 2H). ^13^C-NMR (150 MHz, DMSO-*d*_6_) δ: 172.20, 161.72 (d, *J* = 240 Hz), 152.53, 147.20, 147.11, 140.48, 140.39, 135.08, 129.59, 129.29, 129.24(×3), 127.80(×2), 127.33(×2), 126.65, 126.12(×2), 124.77(×2), 119.07, 115.85, 115.70, 113.94(×2), 54.34, 54.35. HRMS calcd. for C_29_H_22_FN_3_O_2_S, [M + H]^+^, 496.1495; found 496.1552. HPLC: *t*_R_ = 14.39 min, 97.20%.

*N-(4-Fluorobenzyl)-N-((4-(4-nitrophenyl)thiazol-2-yl)methyl)-[1,1′-biphenyl]-3-amine* (**26**). Light yellow solid, yield: 59.8%. m.p. 149.5–150.8 °C. ^1^H-NMR (600 MHz, CDCl_3_) δ: 8.27 (d, *J* = 8.8 Hz, 2H), 8.04 (d, *J* = 8.8 Hz, 2H), 7.59 (s, 1H), 7.48 (d, *J* = 7.3 Hz, 2H), 7.37 (t, *J* = 7.5 Hz, 2H), 7.32~7.27 (m, 4H), 7.10 (s, 1H), 7.06~7.02 (m, 3H), 6.83 (dd, *J* = 8.3, 2.3 Hz, 1H), 4.93 (s, 2H), 4.75 (s, 2H). ^13^C-NMR (150 MHz, DMSO-*d*_6_) δ: 172.05, 161.70 (d, *J* = 240 Hz), 152.33, 148.25, 147.12, 141.53, 141.24, 140.46, 135.22, 130.16, 129.37, 129.31, 129.25(×2), 127.82, 127.32(×2), 127.12(×2), 124.76(×2), 119.21, 116.52, 115.83, 115.69, 112.88, 112.12, 55.47, 53.52. HRMS calcd. for C_29_H_22_FN_3_O_2_S, [M + H]^+^, 496.1495; found 496.1567. HPLC: *t*_R_ = 13.88 min, 96.81%.

*N-(4-Fluorobenzyl)-N-((4-(4-nitrophenyl)thiazol-2-yl)methyl)-[1,1′-biphenyl]-2-amine* (**27**). Colourless oil, yield: 50.4%. ^1^H-NMR (600 MHz, DMSO-*d*_6_) δ: 8.31 (s, 1H), 8.28 (d, *J* = 8.9 Hz, 2H), 8.17 (d, *J* = 8.9 Hz, 2H), 7.61 (d, *J* = 7.2 Hz, 2H), 7.49 (t, *J* = 7.6 Hz, 2H), 7.37 (t, *J* = 7.4 Hz, 1H), 7.23~7.19 (m, 2H), 7.13~7.10 (m, 3H), 7.10~7.04 (m, 3H), 4.31 (s, 2H), 4.00 (s, 2H). ^13^C-NMR (150 MHz, DMSO-*d*_6_) δ: 171.99, 161.71 (d, *J* = 240 Hz), 160.91, 152.34, 148.26, 147.09, 141.54, 141.24, 140.45, 135.17, 130.16, 129.35, 129.30, 129.24(×2), 127.80, 127.29(×2), 127.11(×2), 124.72(×2), 119.17, 116.54, 115.82, 115.68, 112.88, 112.13, 54.48, 53.51. HRMS calcd. for C_29_H_22_FN_3_O_2_S, [M + H]^+^, 496.1495; found 496.1563. HPLC: *t*_R_ = 15.05 min, 95.79%.

*N-(4-Fluorobenzyl)-N-((4-(4-nitrophenyl)thiazol-2-yl)methyl)-3-(thiophen-2-yl)aniline* (**28**). Dark yellow solid, yield: 63.1%. m.p. 143.9–148.8 °C. ^1^H-NMR (600 MHz, DMSO-*d*_6_) δ: 8.38 (s, 1H), 8.32 (d, *J* = 8.7 Hz, 2H), 8.24 (d, *J* = 8.7 Hz, 2H), 7.46 (d, *J* = 5.0 Hz, 1H), 7.41 (dd, *J* = 8.0, 5.8 Hz, 2H), 7.35 (d, *J* = 3.5 Hz, 1H), 7.18 (dd, *J* = 16.3, 8.2 Hz, 3H), 7.07 (t, *J* = 4.1 Hz, 2H), 6.96 (d, *J* = 7.5 Hz, 1H), 6.75 (dd, *J* = 8.3, 1.9 Hz, 1H), 5.12 (s, 2H), 4.84 (s, 2H). ^13^C-NMR (150 MHz, DMSO-*d*_6_) δ: 171.84, 161.71 (d, *J* = 240 Hz), 152.38, 148.22, 148.12, 144.44, 140.46, 135.14, 134.90, 130.34, 129.36, 129.30, 128.75, 127.33(×2), 125.90, 124.76(×2), 123.91, 119.18, 115.85, 115.70, 115.31, 113.07, 110.66, 54.51, 53.55. HRMS calcd. for C_27_H_20_FN_3_O_2_S_2_, [M + H]^+^, 502.1059; found 502.1118. HPLC: *t*_R_ = 14.17 min, 98.03%.

*N-(4-Fluorobenzyl)-3-(1-methyl-1H-pyrazol-5-yl)-N-((4-(4-nitrophenyl)thiazol-2-yl)methyl)aniline* (**29**) Brown solid, yield: 68.9%. m.p. 102.2–104.5 °C. ^1^H-NMR (600 MHz, DMSO-*d*_6_) δ: 8.38 (s, 1H), 8.32 (d, *J* = 8.9 Hz, 2H), 8.22 (d, *J* = 8.9 Hz, 2H), 7.43~7.37 (m, 3H), 7.26 (t, *J* = 8.0 Hz, 1H), 7.19 (t, *J* = 8.8 Hz, 2H), 6.87 (dd, *J* = 8.4, 2.2 Hz, 1H), 6.84 (s, 1H), 6.80 (d, *J* = 7.5 Hz, 1H), 6.24 (d, *J* = 1.8 Hz, 1H), 5.14 (s, 2H), 4.85 (s, 2H), 3.59 (s, 3H). ^13^C-NMR (150 MHz, DMSO-*d*_6_) δ: 171.92, 161.72 (d, *J* = 241 Hz), 152.46, 148.22, 147.11, 143.64, 140.41, 138.26, 134.99, 131.29, 130.05, 129.27, 129.22, 127.29(×2), 124.73(×2), 119.09, 117.99, 115.84, 115.70, 115.04, 113.58, 106.05, 54.48, 53.64, 49.08. HRMS calcd. for C_27_H_22_FN_5_O_2_S, [M + H]^+^, 500.1556; found 500.1626. HPLC: *t*_R_ = 6.76 min, 95.89%.

*N-(4-Fluorobenzyl)-3,4-dimethoxy-N-((4-(4-nitrophenyl)thiazol-2-yl)methyl)aniline* (**30**). Light yellow solid, yield: 63.8%. m.p. 111.0–112.8 °C. ^1^H-NMR (400 MHz, DMSO-*d*_6_) δ: 8.37 (s, 1H), 8.32 (d, *J* = 9.0 Hz, 2H), 8.22 (d, *J* = 8.9 Hz, 2H), 7.39 (dd, *J* = 8.4, 5.7 Hz, 2H), 7.17 (t, *J* = 8.8 Hz, 2H), 6.75 (d, *J* = 8.8 Hz, 1H), 6.56 (d, *J* = 2.7 Hz, 1H), 6.28 (dd, *J* = 8.8, 2.7 Hz, 1H), 4.94 (s, 2H), 4.66 (s, 2H), 3.62 (d, *J* = 4.1 Hz, 6H). ^13^C-NMR (150 MHz, DMSO-*d*_6_) δ: 163.14, 162.02 (d, *J* = 241 Hz), 160.86, 153.36, 149.87, 142.92, 142.05, 135.47, 131.23, 129.64, 128.44, 128.38, 116.21, 116.07, 115.67(×2), 115.53, 114.48, 113.97, 106.27, 101.19, 56.55, 55.80, 55.26, 54.15. HRMS calcd. for C_25_H_22_FN_3_O_4_S, [M + Na]^+^, 502.1213; found 502.1217. HPLC: *t*_R_ = 7.59 min, 98.98%.

*N-(4-Fluorobenzyl)-N-((4-(4-fluorophenyl)thiazol-2-yl)methyl)-3,4-dimethoxyaniline* (**31**). Light yellow oil, yield: 71.1%. ^1^H-NMR (400 MHz, DMSO-*d*_6_) δ: 8.05~7.94 (m, 3H), 7.38 (s, 2H), 7.27 (t, *J* = 7.6 Hz, 2H), 7.16 (t, *J* = 7.7 Hz, 2H), 6.74 (d, *J* = 8.1 Hz, 1H), 6.57 (s, 1H), 6.28 (d, *J* = 8.4 Hz, 1H), 4.90 (s, 2H), 4.65 (s, 2H), 3.63 (d, *J* = 2.7 Hz, 6H). ^13^C-NMR (150 MHz, DMSO-*d*_6_) δ: 171.42, 162.33 (d, *J* = 243 Hz), 162.47~160.86 (d, *J* = 241 Hz), 153.36, 149.87, 142.92, 142.05, 135.46, 131.24, 129.69, 129.64, 128.44, 128.38, 116.21, 116.07, 115.67, 115.53, 114.48, 113.97, 106.27, 101.19, 56.55, 55.80, 55.26, 54.15. HRMS calcd. for C_25_H_22_F_2_N_2_O_2_S, [M + Na]^+^, 475.1268; found 475.1324. HPLC: *t*_R_ = 4.87 min, 99.31%.

*N-(4-Fluorobenzyl)-3,4-dimethoxy-N-((4-(4-(trifluoromethyl)phenyl)thiazol-2-yl)methyl)aniline* (**32**). White solid, yield: 69.9%. m.p. 88.7–89.5 °C. ^1^H-NMR (400 MHz, DMSO-*d*_6_) δ: 8.24 (s, 1H), 8.17 (d, *J* = 8.1 Hz, 2H), 7.81 (d, *J* = 8.3 Hz, 2H), 7.39 (dd, *J* = 8.5, 5.7 Hz, 2H), 7.17 (t, *J* = 8.9 Hz, 2H), 6.75 (d, *J* = 8.8 Hz, 1H), 6.56 (d, *J* = 2.8 Hz, 1H), 6.28 (dd, *J* = 8.8, 2.8 Hz, 1H), 4.93 (s, 2H), 4.66 (s, 2H), 3.62 (d, *J* = 3.5 Hz, 6H). ^13^C-NMR (150 MHz, DMSO-*d*_6_) δ: 172.03, 161.67 (d, *J* = 241 Hz), 152.76, 149.88, 142.87, 142.11, 138.26, 135.43, 135.42, 129.71, 129.66, 128.44, 126.94(×2), 126.28, 117.36, 115.67, 115.53, 113.94, 106.34, 101.24, 56.53, 55.80, 55.30, 54.16, 49.06. HRMS calcd. for C_26_H_22_F_4_N_2_O_2_S, [M + Na]^+^, 525.1236; found 525.1303. HPLC: *t*_R_ = 9.44 min, 98.71%.

*4-(2-(((3,4-Dimethoxyphenyl)(4-fluorobenzyl)amino)methyl)thiazol-4-yl)benzonitrile* (**33**). Yellow solid, yield: 69.9%. m.p. 105.4–106.6 °C. ^1^H-NMR (600 MHz, DMSO-*d*_6_) δ: 8.28 (s, 1H), 8.14 (d, *J* = 8.1 Hz, 2H), 7.91 (d, *J* = 8.3 Hz, 2H), 7.40~7.36 (m, 2H), 7.16 (t, *J* = 9.7 Hz, 2H), 6.74 (d, *J* = 8.8 Hz, 1H), 6.56 (d, *J* = 2.8 Hz, 1H), 6.28 (dd, *J* = 8.8, 2.9 Hz, 1H), 4.92 (s, 2H), 4.65 (s, 2H), 3.62 (d, *J* = 5.8 Hz, 6H). ^13^C-NMR (150 MHz, DMSO-*d*_6_) δ: 172.18, 161.67 (d, *J* = 241 Hz), 152.54, 149.88, 142.84, 142.12, 138.66, 135.42, 133.38(×2), 129.72, 129.67, 127.00, 119.35, 118.27, 115.68, 115.54, 113.95, 110.60, 106.34, 101.25, 56.53, 55.82, 55.29, 54.12, 49.07. HRMS calcd. for C_26_H_22_FN_3_O_2_S, [M + Na]^+^, 482.1314; found 482.1375. HPLC: *t*_R_ = 5.87 min, 97.66%.

*N-(4-Fluorobenzyl)-3,4-dimethoxy-N-((4-(3-nitrophenyl)thiazol-2-yl)methyl)aniline* (**34**). Light yellow solid, yield: 53.7%. m.p. 120.5–121.6 °C. ^1^H-NMR (600 MHz, CDCl_3_) δ: 8.74 (t, *J* = 1.9 Hz, 1H), 8.21~8.16 (m, 2H), 7.59 (t, *J* = 8.0 Hz, 1H), 7.56 (s, 1H), 7.32 (dd, *J* = 8.5, 5.4 Hz, 2H), 7.05~7.01 (m, 2H), 6.76~6.74 (m, 1H), 6.56 (d, *J* = 2.1 Hz, 1H), 6.43 (dd, *J* = 8.7, 2.5 Hz, 1H), 4.80 (s, 2H), 4.58 (s, 2H), 3.80 (s, 3H), 3.76 (s, 3H). ^13^C-NMR (150 MHz, DMSO-*d*_6_) δ: 172.21, 161.68 (d, *J* = 240 Hz), 151.94, 149.89, 148.86, 142.85, 142.14, 136.09, 135.44, 132.50, 130.98, 129.72, 129.69, 123.00, 120.71, 117.40, 115.68, 115.54, 113.95, 106.37, 101.28, 56.53, 55.81, 55.33, 54.16. HRMS calcd. for C_25_H_22_FN_3_O_4_S, [M + Na]^+^, 502.1213; found 502.1272. HPLC: *t*_R_ = 6.87 min, 95.41%.

*N-(4-Fluorobenzyl)-3,4-dimethoxy-N-((4-(2-nitrophenyl)thiazol-2-yl)methyl)aniline* (**35**). Light yellow oil, yield: 54.6%. ^1^H-NMR (600 MHz, DMSO-*d*_6_) δ: 7.95 (s, 1H), 7.90 (dd, *J* = 8.0, 1.0 Hz, 1H), 7.83~7.78 (m, 1H), 7.76~7.72 (m, 1H), 7.64~7.60 (m, 1H), 7.36 (dd, *J* = 8.5, 5.6 Hz, 2H), 7.18~7.12 (m, 2H), 6.74 (d, *J* = 8.8 Hz, 1H), 6.52 (d, *J* = 2.8 Hz, 1H), 6.27 (dd, *J* = 8.8, 2.8 Hz, 1H), 4.78 (s, 2H), 4.59 (s, 2H), 3.63 (d, *J* = 10.9 Hz, 6H). ^13^C-NMR (150 MHz, DMSO-*d*_6_) δ: 171.08, 161.68 (d, *J* = 241 Hz), 149.96, 149.86, 149.21, 142.87, 142.20, 135.35, 132.97, 131.17, 129.85, 129.85, 129.79, 128.17, 124.43, 118.86, 115.65, 115.51, 113.85, 106.64, 101.40, 56.51, 55.76, 55.03, 49.07. HRMS calcd. for C_25_H_22_FN_3_O_4_S, [M + Na]^+^, 502.1213; found 502.1277. HPLC: *t*_R_ = 5.44 min, 98.06%.

*N-((4-(4-Bromophenyl)thiazol-2-yl)methyl)-N-(4-fluorobenzyl)-3,4-dimethoxyaniline* (**36**). Dark yellow oil, yield: 71.6%. ^1^H-NMR (400 MHz, DMSO-*d*_6_) δ: 8.06 (s, 1H), 7.91 (d, *J* = 8.6 Hz, 2H), 7.64 (d, *J* = 8.6 Hz, 2H), 7.38 (dd, *J* = 8.5, 5.7 Hz, 2H), 7.16 (t, *J* = 8.9 Hz, 2H), 6.74 (d, *J* = 8.8 Hz, 1H), 6.56 (d, *J* = 2.7 Hz, 1H), 6.27 (dd, *J* = 8.8, 2.8 Hz, 1H), 4.90 (s, 2H), 4.65 (s, 2H), 3.62 (d, *J* = 4.2 Hz, 6H). ^13^C-NMR (150 MHz, DMSO-*d*_6_) δ: 171.65, 161.67 (d, *J* = 240 Hz), 153.17, 149.88, 142.89, 142.07, 135.45, 133.79, 132.22(×2), 129.69, 129.64, 128.38(×2), 121.54, 115.67, 115.53(×2), 113.97, 106.28, 101.20, 56.55, 55.81, 55.26, 54.16. HRMS calcd. for C_25_H_22_BrFN_2_O_2_S, [M + Na]^+^, 535.0467; found 535.0539. HPLC: *t*_R_ = 11.61 min, 97.19%.

*N-(4-Fluorobenzyl)-3,4-dimethoxy-N-((4-(4-(1-methyl-1H-pyrazol-4-yl)phenyl)thiazol-2-yl)methyl)aniline* (**37**). Brown solid, yield: 42.1%. m.p. 126.1–130.3 °C. ^1^H-NMR (600 MHz, DMSO-*d*_6_) δ: 8.18 (s, 1H), 7.96 (s, 1H), 7.94~7.90 (m, 3H), 7.63 (d, *J* = 8.4 Hz, 2H), 7.39 (dd, *J* = 8.5, 5.6 Hz, 2H), 7.16 (t, *J* = 8.8 Hz, 2H), 6.74 (d, *J* = 8.8 Hz, 1H), 6.57 (d, *J* = 2.8 Hz, 1H), 6.28 (dd, *J* = 8.8, 2.8 Hz, 1H), 4.91 (s, 2H), 4.65 (s, 2H), 3.87 (s, 3H), 3.62 (d, *J* = 9.6 Hz, 6H). ^13^C-NMR (150 MHz, DMSO-*d*_6_) δ: 170.07, 160.59 (d, *J* = 240 Hz), 153.23, 148.79, 141.88, 140.95, 135.47, 131.62, 131.44, 131.16, 130.91, 130.84, 128.56, 128.20, 128.12, 127.30, 125.77, 124.57, 120,89, 114.59, 114.45, 112.90, 105.18, 100.11, 55.48, 54.73, 54.17, 53.13, 47.99. HRMS calcd. for C_29_H_27_FN_4_O_2_S, [M + Na]^+^, 537.1736; found 537.1822. HPLC: *t*_R_ = 5.19 min, 95.89%.

*N-((4-(4-(3,5-Dimethylisoxazol-4-yl)phenyl)thiazol-2-yl)methyl)-N-(4-fluorobenzyl)-3,4-dimethoxyaniline* (**38**). Yellow solid, yield: 51.6%. m.p. 69.0–69.8 °C. ^1^H-NMR (400 MHz, DMSO-*d*_6_) δ: 8.08~8.02 (m, 3H), 7.46 (d, *J* = 8.4 Hz, 2H), 7.39 (dd, *J* = 8.5, 5.6 Hz, 2H), 7.16 (t, *J* = 8.9 Hz, 2H), 6.74 (d, *J* = 8.8 Hz, 1H), 6.58 (d, *J* = 2.8 Hz, 1H), 6.29 (dd, *J* = 8.8, 2.8 Hz, 1H), 4.92 (s, 2H), 4.66 (s, 2H), 3.63 (d, *J* = 9.1 Hz, 6H), 2.43 (s, 3H), 2.26 (s, 3H). ^13^C-NMR (150 MHz, DMSO-*d*_6_) δ: 171.35, 165.67, 161.68 (d, *J* = 240 Hz), 158.59, 153.93, 149.89, 142.95, 142.07, 135.48, 133.68, 129.86, 129.71(×2), 129.65, 126.79(×2), 116.07, 115.67, 115.53, 115.07, 113.97, 106.29, 101.23, 56.55, 55.81, 55.28, 54.16, 49.07, 11.90, 11.02. HRMS calcd. for C_30_H_28_FN_3_O_3_S, [M + Na]^+^, 552.1733; found 552.1817. HPLC: *t*_R_ = 7.26 min, 96.49%.

*N-(4-Fluorobenzyl)-3,4-dimethoxy-N-((4-(4-(1-methyl-1H-pyrazol-5-yl)phenyl)thiazol-2-yl)methyl)aniline* (**39**). Dark yellow solid, yield: 49.0%. m.p. 102.4–103.3 °C. ^1^H-NMR (400 MHz, DMSO-*d*_6_) δ: 8.11 (s, 1H), 8.06 (d, *J* = 8.3 Hz, 2H), 7.62 (d, *J* = 8.3 Hz, 2H), 7.49 (d, *J* = 1.8 Hz, 1H), 7.40 (dd, *J* = 8.4, 5.7 Hz, 2H), 7.17 (t, *J* = 8.8 Hz, 2H), 6.75 (d, *J* = 8.8 Hz, 1H), 6.57 (d, *J* = 2.8 Hz, 1H), 6.47 (d, *J* = 1.8 Hz, 1H), 6.29 (dd, *J* = 8.8, 2.8 Hz, 1H), 4.93 (s, 2H), 4.67 (s, 2H), 3.90 (s, 3H), 3.63 (d, *J* = 5.9 Hz, 6H). ^13^C-NMR (150 MHz, DMSO-*d*_6_) δ: 171.50, 161.67 (d, *J* = 241 Hz), 153.73, 149.88, 142.93, 142.78, 142.06, 138.44, 135.47, 134.38, 130.02, 129.71, 129.66, 129.27(×2), 126.67(×2), 115.68, 115.54, 115.48, 113.98, 106.34, 106.29, 101.22, 56.56, 55.82, 55.28, 54.17, 49.07. HRMS calcd. for C_29_H_27_FN_4_O_2_S, [M + Na]^+^, 537.1736; found 537.1815. HPLC: *t*_R_ = 5.84 min, 99.44%.

*N-(4-Fluorobenzyl)-3,4-dimethoxy-N-((4-(4-(thiophen-2-yl)phenyl)thiazol-2-yl)methyl)aniline* (**40**). Dark yellow solid, yield: 43.7%. m.p. 115.8–119.0 °C. ^1^H-NMR (400 MHz, DMSO-*d*_6_) δ: 8.04 (s, 1H), 7.99 (d, *J* = 8.4 Hz, 2H), 7.74 (d, *J* = 8.4 Hz, 2H), 7.61~7.56 (m, 2H), 7.39 (dd, *J* = 8.4, 5.7 Hz, 2H), 7.20~7.14 (m, 3H), 6.75 (d, *J* = 8.8 Hz, 1H), 6.57 (d, *J* = 2.7 Hz, 1H), 6.28 (dd, *J* = 8.8, 2.7 Hz, 1H), 4.92 (s, 2H), 4.66 (s, 2H), 3.63 (d, *J* = 5.9 Hz, 6H). ^13^C-NMR (150 MHz, DMSO-*d*_6_) δ: 171.41, 161.68 (d, *J* = 241 Hz), 153.90, 149.89, 143.41, 142.95, 142.08, 135.45, 133.71, 133.67, 129.69, 129.64, 129.07, 127.05(×2), 126.31, 126.19(×2), 124.34, 115.67, 115.53, 114.84, 113.97, 106.31, 101.22, 56.54, 55.80, 55.28, 54.21. HRMS calcd. for C_29_H_25_FN_2_O_2_S_2_, [M + Na]^+^, 539.1239; found 539.1324. HPLC: *t*_R_ = 13.67 min, 97.82%.

*N-(4-Fluorobenzyl)-3,4-dimethoxy-N-((4-(naphthalen-2-yl)thiazol-2-yl)methyl)aniline* (**41**). Light yellow oil, yield: 76.1%. ^1^H-NMR (400 MHz, DMSO-*d*_6_) δ: 8.54 (s, 1H), 8.14~8.08 (m, 2H), 7.98 (t, *J* = 8.4 Hz, 2H), 7.92 (d, *J* = 7.3 Hz, 1H), 7.57~7.48 (m, 2H), 7.40 (dd, *J* = 8.3, 5.7 Hz, 2H), 7.17 (t, *J* = 8.8 Hz, 2H), 6.74 (d, *J* = 8.8 Hz, 1H), 6.60 (d, *J* = 2.6 Hz, 1H), 6.30 (dd, *J* = 8.8, 2.7 Hz, 1H), 4.95 (s, 2H), 4.67 (s, 2H), 3.63 (d, *J* = 12.8 Hz, 6H). ^13^C-NMR (150 MHz, DMSO-*d*_6_) δ: 171.60, 161.68 (d, *J* = 241 Hz), 154.35, 149.90, 142.96, 142.09, 135.50, 133.66, 133.07, 132.04, 129.71, 129.66, 128.84, 128.70, 128.09, 127.04, 126.71, 125.03, 124.61, 115.69, 115.54, 115.28, 113.99, 106.32, 101.25, 56.55, 55.82, 55.31, 49.07. HRMS calcd. for C_29_H_25_FN_2_O_2_S, [M + Na]^+^, 507.1518; found 507.1588. HPLC: *t*_R_ = 11.73 min, 96.23%.

*N-(4-Fluorobenzyl)-3,4-dimethoxy-N-((4-(p-tolyl)thiazol-2-yl)methyl)aniline* (**42**). Yellow solid, yield: 67.1%. m.p. 105.3–107.0 °C. ^1^H-NMR (400 MHz, DMSO-*d*_6_) δ: 7.90 (s, 1H), 7.85 (d, *J* = 8.1 Hz, 2H), 7.38 (dd, *J* = 8.5, 5.7 Hz, 2H), 7.25 (d, *J* = 8.0 Hz, 2H), 7.16 (t, *J* = 8.9 Hz, 2H), 6.74 (d, *J* = 8.8 Hz, 1H), 6.57 (d, *J* = 2.8 Hz, 1H), 6.27 (dd, *J* = 8.8, 2.8 Hz, 1H), 4.90 (s, 2H), 4.65 (s, 2H), 3.62 (d, *J* = 5.9 Hz, 6H), 2.33 (s, 3H). ^13^C-NMR (150 MHz, DMSO-*d*_6_) δ: 171.06, 161.67 (d, *J* = 240 Hz), 160.87, 154.50, 149.87, 142.97, 142.03, 137.76, 135.48, 131.97, 129.82(×2), 129.67, 129.62(×2), 126.31, 115.66, 115.52, 113.98, 113.75, 106.25, 101.17, 56.55, 55.78, 55.25, 54.21, 21.28. HRMS calcd. for C_26_H_25_FN_2_O_2_S, [M + Na]^+^, 471.1518; found 471.1578. HPLC: *t*_R_ = 9.58 min, 97.85%.

*N-(4-Fluorobenzyl)-3,4-dimethoxy-N-((4-(4-methoxyphenyl)thiazol-2-yl)methyl)aniline* (**43**). Dark yellow solid, yield: 83.7%. m.p. 76.3–79.0 °C. ^1^H-NMR (400 MHz, DMSO-*d*_6_) δ: 7.88 (d, *J* = 8.8 Hz, 2H), 7.82 (s, 1H), 7.39 (dd, *J* = 8.5, 5.7 Hz, 2H), 7.16 (t, *J* = 8.8 Hz, 2H), 7.00 (d, *J* = 8.8 Hz, 2H), 6.74 (d, *J* = 8.8 Hz, 1H), 6.56 (d, *J* = 2.8 Hz, 1H), 6.27 (dd, *J* = 8.8, 2.8 Hz, 1H), 4.89 (s, 2H), 4.65 (s, 2H), 3.80 (s, 3H), 3.62 (d, *J* = 4.0 Hz, 6H). ^13^C-NMR (150 MHz, DMSO-*d*_6_) δ: 170.93, 161.67 (d, *J* = 241 Hz), 159.56, 154.34, 149.88, 142.99, 142.03, 135.48, 129.67, 129.61, 127.73(×2), 127.48, 115.66, 115.52, 114.60(×2), 113.98, 112.54, 106.25, 101.17, 56.54, 55.79, 55.60, 55.25, 54.20. HRMS calcd. for C_26_H_25_FN_2_O_3_S, [M + Na]^+^, 487.1468; found 487.1535. HPLC: *t*_R_ = 7.05 min, 98.64%.

*N-((4-(4-Aminophenyl)thiazol-2-yl)methyl)-N-(4-fluorobenzyl)-3,4-dimethoxyaniline* (**44**). Light yellow solid, yield: 87.3%. m.p. 144.7–146.2 °C. ^1^H-NMR (400 MHz, DMSO-*d*_6_) δ: 7.61 (d, *J* = 8.5 Hz, 2H), 7.55 (s, 1H), 7.40~7.36 (m, 2H), 7.16 (dd, *J* = 12.3, 5.4 Hz, 2H), 6.74 (d, *J* = 8.8 Hz, 1H), 6.60 (d, *J* = 8.6 Hz, 2H), 6.55 (d, *J* = 2.8 Hz, 1H), 6.25 (dd, *J* = 8.8, 2.8 Hz, 1H), 5.28 (s, 2H), 4.86 (s, 2H), 4.64 (s, 2H), 3.62 (d, *J* = 4.2 Hz, 6H). ^13^C-NMR (150 MHz, DMSO-*d*_6_) δ: 170.23, 161.65 (d, *J* = 241 Hz), 155.52, 149.86, 149.12, 143.04, 141.95, 135.54, 129.65, 129.60, 127.41(×2), 122.83, 115.65, 115.51, 114.27(×2), 114.00, 109.74, 106.17, 101.11, 56.57, 55.78, 55.21, 54.23. HRMS calcd. for C_25_H_24_FN_3_O_2_S, [M + Na]^+^, 472.1471; found 472.1538. HPLC: *t*_R_ = 4.25 min, 95.78%.

*N-(4-(2-(((3,4-Dimethoxyphenyl)(4-fluorobenzyl)amino)methyl)thiazol-4-yl)phenyl)-2,2,2-trifluoroacetamide* (**45**). Light yellow solid, yield: 75.8%. m.p. 143.6–145.8 °C. ^1^H-NMR (600 MHz, DMSO-*d*_6_) δ: 11.34 (s, 1H), 8.01~7.96 (m, 3H), 7.76 (d, *J* = 8.7 Hz, 2H), 7.38 (dd, *J* = 8.4, 5.7 Hz, 2H), 7.16 (t, *J* = 8.8 Hz, 2H), 6.74 (d, *J* = 8.8 Hz, 1H), 6.56 (d, *J* = 2.8 Hz, 1H), 6.28 (dd, *J* = 8.8, 2.8 Hz, 1H), 4.90 (s, 2H), 4.65 (s, 2H), 3.62 (d, *J* = 6.5 Hz, 6H). ^13^C-NMR (150 MHz, DMSO-*d*_6_) δ: 171.42, 161.67 (d, *J* = 240 Hz), 155.02, 154.78, 153.72, 149.88, 142.93, 142.06, 136.43, 135.47, 131.92, 129.69, 129.64, 126.95(×2), 121.69, 115.67, 115.53, 114.62, 113.98, 106.28, 101.20, 56.55, 55.80, 55.26, 54.19, 49.06. HRMS calcd. for C_27_H_23_F_4_N_3_O_3_S, [M + Na]^+^, 568.1294; found 568.1379. HPLC: *t*_R_ = 5.34 min, 97.66%.

*N-(4-(2-(((3,4-Dimethoxyphenyl)(4-fluorobenzyl)amino)methyl)thiazol-4-yl)phenyl)acetamide* (**46**). Light yellow solid, yield: 86.1%. m.p. 134.6–137.0 °C. ^1^H-NMR (600 MHz, DMSO-*d*_6_) δ: 10.03 (s, 1H), 7.88~7.83 (m, 3H), 7.64 (d, *J* = 8.6 Hz, 2H), 7.38 (dd, *J* = 8.4, 5.7 Hz, 2H), 7.16 (t, *J* = 8.8 Hz, 2H), 6.74 (d, *J* = 8.8 Hz, 1H), 6.55 (d, *J* = 2.8 Hz, 1H), 6.27 (dd, *J* = 8.8, 2.8 Hz, 1H), 4.89 (s, 2H), 4.64 (s, 2H), 3.62 (d, *J* = 6.2 Hz, 6H), 2.06 (s, 3H). ^13^C-NMR (150 MHz, DMSO-*d*_6_) δ: 171.06, 168.81, 161.66 (d, *J* = 241 Hz), 154.29, 149.87, 142.95, 142.03, 139.57, 135.48, 129.69, 129.63, 129.48, 126.81(×2), 119.49(×2), 115.66, 115.52, 113.98, 113.25, 106.26, 101.18, 56.55, 55.80, 55.24, 54.20, 49.07. HRMS calcd. for C_27_H_26_FN_3_O_3_S, [M + Na]^+^, 514.1577; found 514.1662. HPLC: *t*_R_ = 3.84 min, 97.51%.

*Methyl(4-(2-(((3,4-dimethoxyphenyl)(4-fluorobenzyl)amino)methyl)thiazol-4-yl)phenyl)glycinate* (**47**). Light yellow solid, yield: 79.4%. m.p. 103.5–105.4 °C. ^1^H-NMR (600 MHz, DMSO-*d*_6_) δ: 7.67 (d, *J* = 8.6 Hz, 2H), 7.59 (s, 1H), 7.37 (dd, *J* = 8.5, 5.6 Hz, 2H), 7.15 (t, *J* = 8.8 Hz, 2H), 6.73 (d, *J* = 8.8 Hz, 1H), 6.60 (d, *J* = 8.7 Hz, 2H), 6.54 (d, *J* = 2.8 Hz, 1H), 6.27~6.22 (m, 2H), 4.86 (s, 2H), 4.63 (s, 2H), 3.96 (d, *J* = 6.4 Hz, 2H), 3.66 (s, 3H), 3.61 (d, *J* = 5.2 Hz, 6H). ^13^C-NMR (150 MHz, DMSO-*d*_6_) δ: 172.19, 170.39, 161.65 (d, *J* = 240 Hz), 155.25, 149.85, 148.47, 143.02, 141.95, 135.53, 129.66, 129.61, 127.37(×2), 123.48, 115.65, 115.51, 114.00, 112.55, 110.26, 106.18, 101.12, 56.57, 55.79, 55.20, 54.22, 52.11, 49.07, 44.89. HRMS calcd. for C_28_H_28_FN_3_O_4_S, [M + Na]^+^, 544.1682; found 544.1757. HPLC: *t*_R_ = 4.77 min, 95.90%.

*Ethyl-4-(4-(2-(((3,4-dimethoxyphenyl)(4-fluorobenzyl)amino)methyl)thiazol-4-yl)phenoxy)butanoate* (**48**). yellow oil, yield: 32.9%. ^1^H-NMR (400 MHz, DMSO-*d*_6_) δ: 7.86 (d, *J* = 8.6 Hz, 2H), 7.81 (s, 1H), 7.38 (dd, *J* = 8.3, 5.8 Hz, 2H), 7.16 (t, *J* = 8.8 Hz, 2H), 6.98 (d, *J* = 8.8 Hz, 2H), 6.74 (d, *J* = 8.8 Hz, 1H), 6.55 (d, *J* = 2.6 Hz, 1H), 6.27 (dd, *J* = 8.8, 2.7 Hz, 1H), 4.89 (s, 2H), 4.64 (s, 2H), 4.08 (q, *J* = 7.1 Hz, 2H), 4.03 (t, *J* = 6.3 Hz, 2H), 3.62 (d, *J* = 3.7 Hz, 6H), 2.47 (t, *J* = 7.3 Hz, 2H), 2.03~1.95 (m, 2H), 1.19 (t, *J* = 7.1 Hz, 3H). ^13^C-NMR (150 MHz, DMSO-*d*_6_) δ: 173.04, 170.93, 161.67 (d, *J* = 240 Hz), 158.77, 154.31, 149.87, 142.98, 142.01, 135.50, 129.68, 129.62, 127.73(×2), 127.50, 115.67, 115.53, 115.09(×2), 113.99, 112.57, 106.23, 101.16, 67.00, 60.36, 56.56, 55.79, 55.24, 54.19, 30.61, 24.70, 14.58. HRMS calcd. for C_31_H_33_FN_2_O_5_S, [M + Na]^+^, 587.1992; found 587.2083. HPLC: *t*_R_ = 8.39 min, 96.53%.

*N-(4-Fluorobenzyl)-3,4-dimethoxy-N-((4-(4-morpholinophenyl)thiazol-2-yl)methyl)aniline* (**49**). White solid, yield: 53.7%. m.p. 139.0–140.4 °C. ^1^H-NMR (600 MHz, DMSO-*d*_6_) δ: 7.81~7.78 (m, 2H), 7.74 (s, 1H), 7.38 (dd, *J* = 8.6, 5.6 Hz, 2H), 7.17~7.14 (m, 2H), 6.99 (d, *J* = 9.0 Hz, 2H), 6.73 (d, *J* = 8.8 Hz, 1H), 6.55 (d, *J* = 2.8 Hz, 1H), 6.26 (dd, *J* = 8.8, 2.9 Hz, 1H), 4.88 (s, 2H), 4.64 (s, 2H), 3.76~3.73 (m, 4H), 3.61 (d, *J* = 5.7 Hz, 6H), 3.17~3.14 (m, 4H). ^13^C-NMR (150 MHz, DMSO-*d*_6_) δ: 170.13, 161.08 (d, *J* = 241 Hz), 154.11, 150.51, 149.28, 142.42, 141.41, 134.93, 129.09, 129.04, 126.65(×2), 125.16, 115.08, 114.44, 114.20(×2), 113.42, 111.12, 105.63, 100.56, 65.93(×2), 55.99, 55.21, 54.65, 53.64, 48.49, 47.92. HRMS calcd. for C_29_H_30_FN_3_O_3_S, [M + Na]^+^, 542.1890; found 542.1975. HPLC: *t*_R_ = 6.29 min, 98.33%.

### 3.4. In Vitro CETP Inhibitory Assay

All tested compounds were dissolved in 100% DMSO. The compound was dissolved totally, and the solution was vibrated hard on an oscillator for more than 30 s and then stored in a nitrogen cabinet. The stock solutions (10 mM) were diluted with DMSO for an eight-point titration (1:5 serial dilutions) in a 96-well dilution plate. The activity was estimated in accordance with the instructions for the CETP inhibitor screening kit and recombinant CETP. The compounds were tested at eight concentrations, and the fluorescence intensities were measured using a fluorometer (ExEm = 465/535 nm). The IC_50_ was determined from a curve fit of the data with each concentration tested three times.

### 3.5. Metabolic Stability Study

Ten microliters (10 μL, 100 μM/L) of compounds and 80 μL of liver microsomes were mixed and incubated at 37 °C for 10 min, and then 10 μL of NADPH regenerating system was added. Samples were obtained at 0 min, 5 min, 10 min, 20 min, 30 min and 60 min. respectively, and 300 μL stop solution (cold in 4 °C, including 100 ng/mL tolbutamide and 100 ng/mL labetalol) was added to terminate the reaction. After oscillating for 10 min, the plates were centrifuged (4000 rpm) at room temperature for 20 min, and the supernatants were used for analysis.

## 4. Conclusions

A series of *N*,*N*-disubstituted-4-arylthiazole-2-methylamine derivatives were designed, synthesized, and evaluated for their inhibitory activity against CETP by a BODIPY-CE fluorescence assay. Compounds **30** and **32** displayed substantial CETP inhibitory activity in vitro with IC_50_ values of 0.79 ± 0.02 μM and 0.97 ± 0.01 μM, respectively, and demonstrated weak human/rat liver microsome stability. This suggests that compounds **30** and **32** could act as potential CETP inhibitors to be used for further optimization.

## Figures and Tables

**Table 1:**
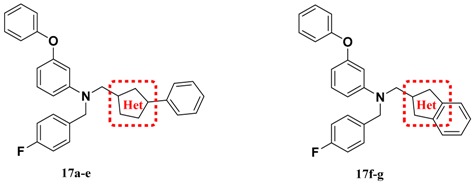
Structures and activities of compounds **17a** to **g**.

Compound	Het	IC_50_ (µM)	Compound	Het	IC_50_ (µM)
**17a**	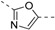	25.73 ± 0.41	**17e**	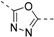	26.87 ± 0.35
**17b**	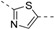	10.60 ± 0.32	**17f**	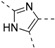	13.68 ± 0.63
**17c**	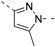	17.10 ± 0.44	**17g**	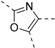	15.23 ± 0.52
**17d**	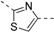	9.03 ± 0.21	Anacetrapib ^a^		0.04 ± 0.01

^a^ Used as a positive control.

**Table 2:**
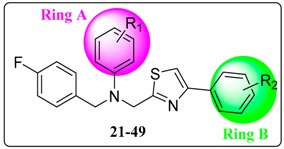
Structures and activities of compounds **21** to **49**.

Compound	R_1_	R_2_	IC_50_ (µM)	Compound	R_1_	R_2_	IC_50_ (µM)
**21**	3-OCH_3_	—	13.96 ± 0.11	**36**	3,4-diOCH_3_	4-Br	8.96 ± 0.15
**22**	3,4-diOCH_3_	—	3.58 ± 0.03	**37**	3,4-diOCH_3_	4- 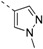	4.02 ± 0.05
**23**	3-OCF_3_	4-NO_2_	31.69 ± 0.25	**38**	3,4-diOCH_3_	4- 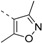	7.57 ± 0.10
**24**	3-CF_3_	4-NO_2_	13.79 ± 0.14	**39**	3,4-diOCH_3_	4- 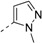	6.38 ± 0.09
**25**	4-Ph	4-NO_2_	9.55 ± 0.08	**40**	3,4-diOCH_3_	4- 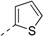	6.03 ± 0.04
**26**	3-Ph	4-NO_2_	8.98 ± 0.05	**41**	3,4-diOCH_3_	[3,4-*a*] benzene	3.51 ± 0.06
**27**	2-Ph	4-NO_2_	1.02 ± 0.01	**42**	3,4-diOCH_3_	4-CH_3_	14.05 ± 0.21
**28**	3- 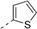	4-NO_2_	7.77 ± 0.13	**43**	3,4-diOCH_3_	4-OCH_3_	10.96 ± 0.31
**29**	3- 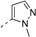	4-NO_2_	24.56 ± 0.22	**44**	3,4-diOCH_3_	4-NH_2_	16.66 ± 0.23
**30**	3,4-diOCH_3_	4-NO_2_	0.79 ± 0.02	**45**	3,4-diOCH_3_	4-NHCOCF_3_	15.30 ± 0.17
**31**	3,4-diOCH_3_	4-F	2.46 ± 0.06	**46**	3,4-diOCH_3_	4-NHCOCH_3_	˃50 ^b^
**32**	3,4-diOCH_3_	4-CF_3_	0.97 ± 0.01	**47**	3,4-diOCH_3_	4- 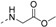	3.54 ± 0.03
**33**	3,4-diOCH_3_	4-CN	1.50 ± 0.03	**48**	3,4-diOCH_3_	4- 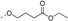	8.40 ± 0.04
**34**	3,4-diOCH_3_	3-NO_2_	1.38 ± 0.07	**49**	3,4-diOCH_3_	4-morpholinyl	˃50 ^b^
**35**	3,4-diOCH_3_	2-NO_2_	6.36 ± 0.12	Anace ^a^			0.04 ± 0.01

^a^ Used as a positive control; ^b^ Considered with no CETP inhibition activity.

**Table 3:** In vitro metabolic stability study of **30**, **32**.

Compound	Human Liver Microsome Rat Liver Microsome					
	CL ^a^ (μL/min/mg)	Remaining (T = 60 min)	Remaining (NCF = 60 min) ^b^	CL ^a^ (μL/min/mg)	Remaining (T = 60 min)	Remaining (NCF = 60 min) ^b^
**30**	48.1	22.9%	68.0%	121.7	3.2%	90.5%
**32**	29.8	39.3%	69.1%	79.3	10.1%	101.3%

^a^ Clearance rate, CL < 100 μL/min/mg means acceptable stability; ^b^ The abbreviation of no co-factor. No NADPH (nicotinamide adenine dinucleotide phosphate) regenerating system is added into NCF (no co-factor) sample (replaced by buffer) during the 60 min incubation.

## Supplementary Materials

Supplementary materials are available online.
