# Supplementary figures and images for: Design, Synthesis, and Biological Evaluation of N,N-Disubstituted-4-arylthiazole-2-methylamine Derivatives as Cholesteryl Ester Transfer Inhibitors
Source: Molecules. 2017 Nov 7;22(11):1925. doi: 10.3390/molecules22111925 (PMC6150381; doi:10.3390/molecules22111925)

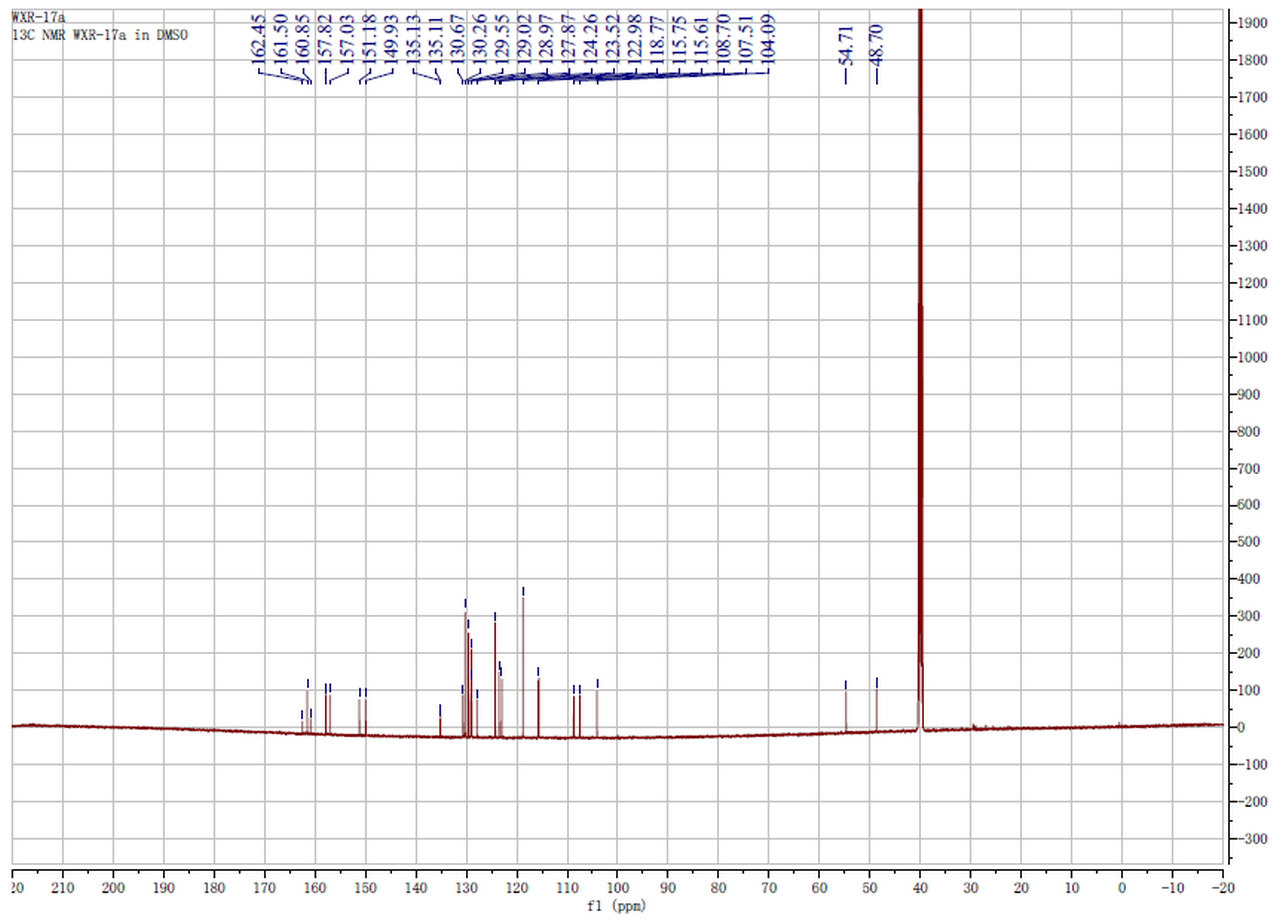

Supplement: Supplementary file 1 [file molecules-22-01925-s001.zip › Molecules-238010--13C NMR/17a.png]

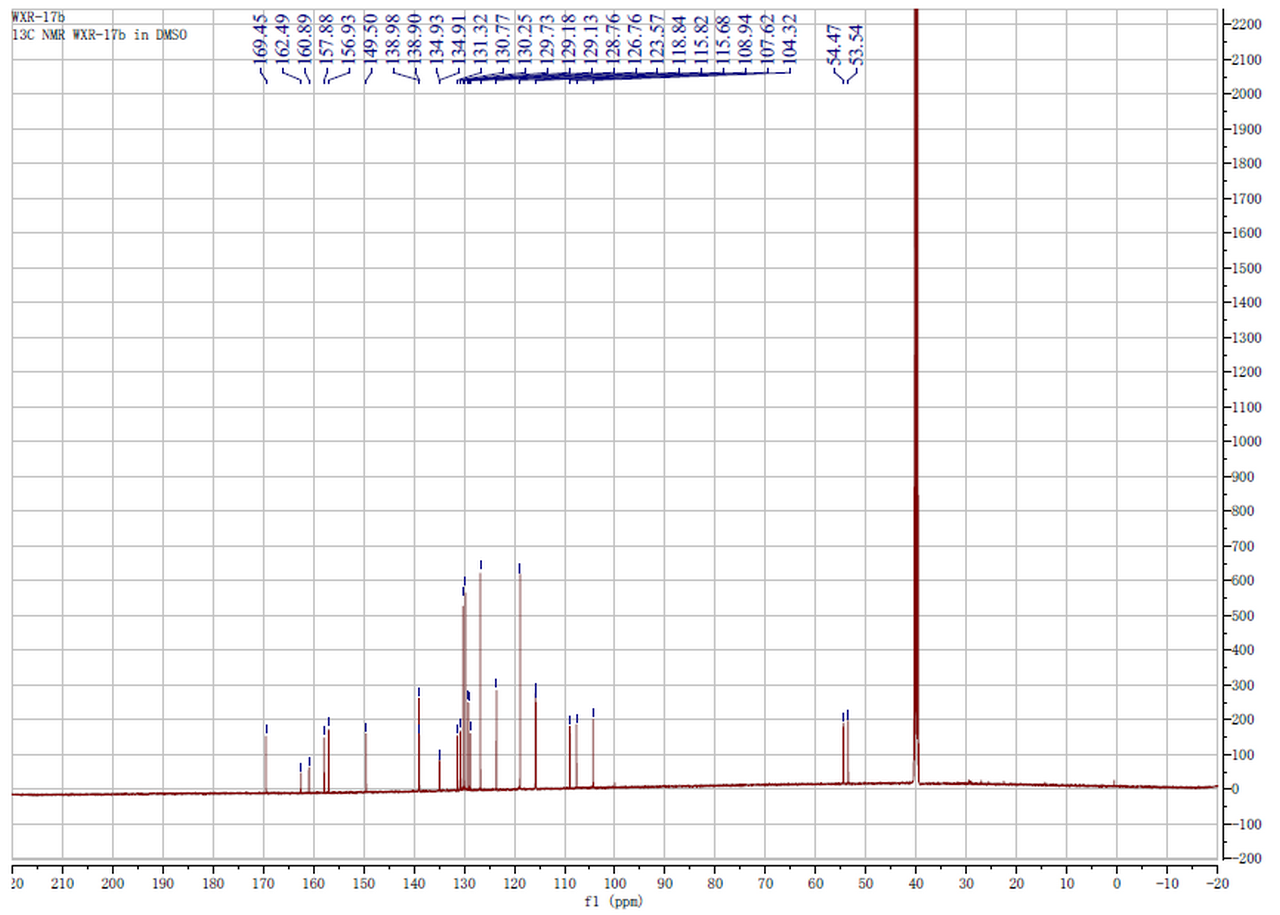

Supplement: Supplementary file 1 [file molecules-22-01925-s001.zip › Molecules-238010--13C NMR/17b.png]

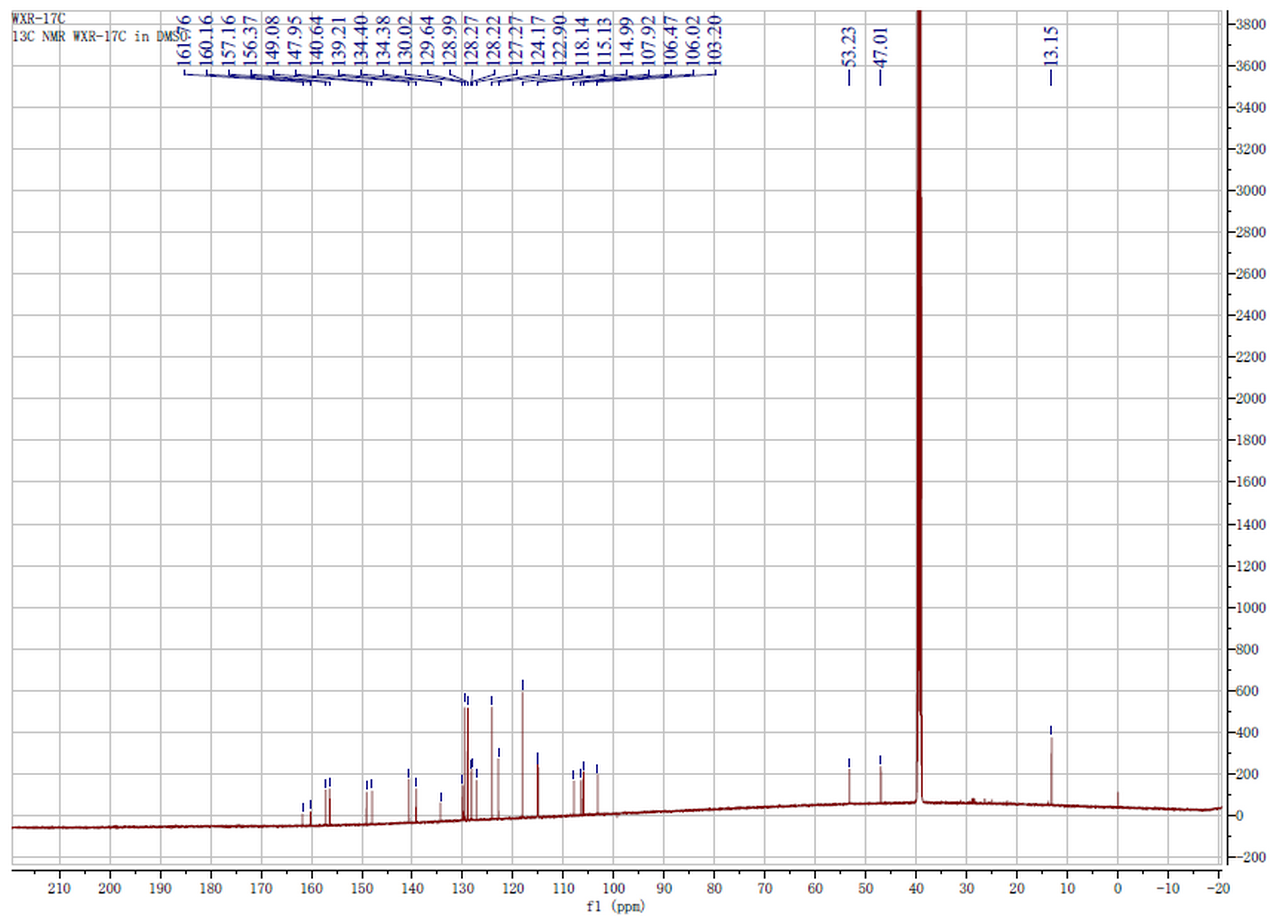

Supplement: Supplementary file 1 [file molecules-22-01925-s001.zip › Molecules-238010--13C NMR/17c.png]

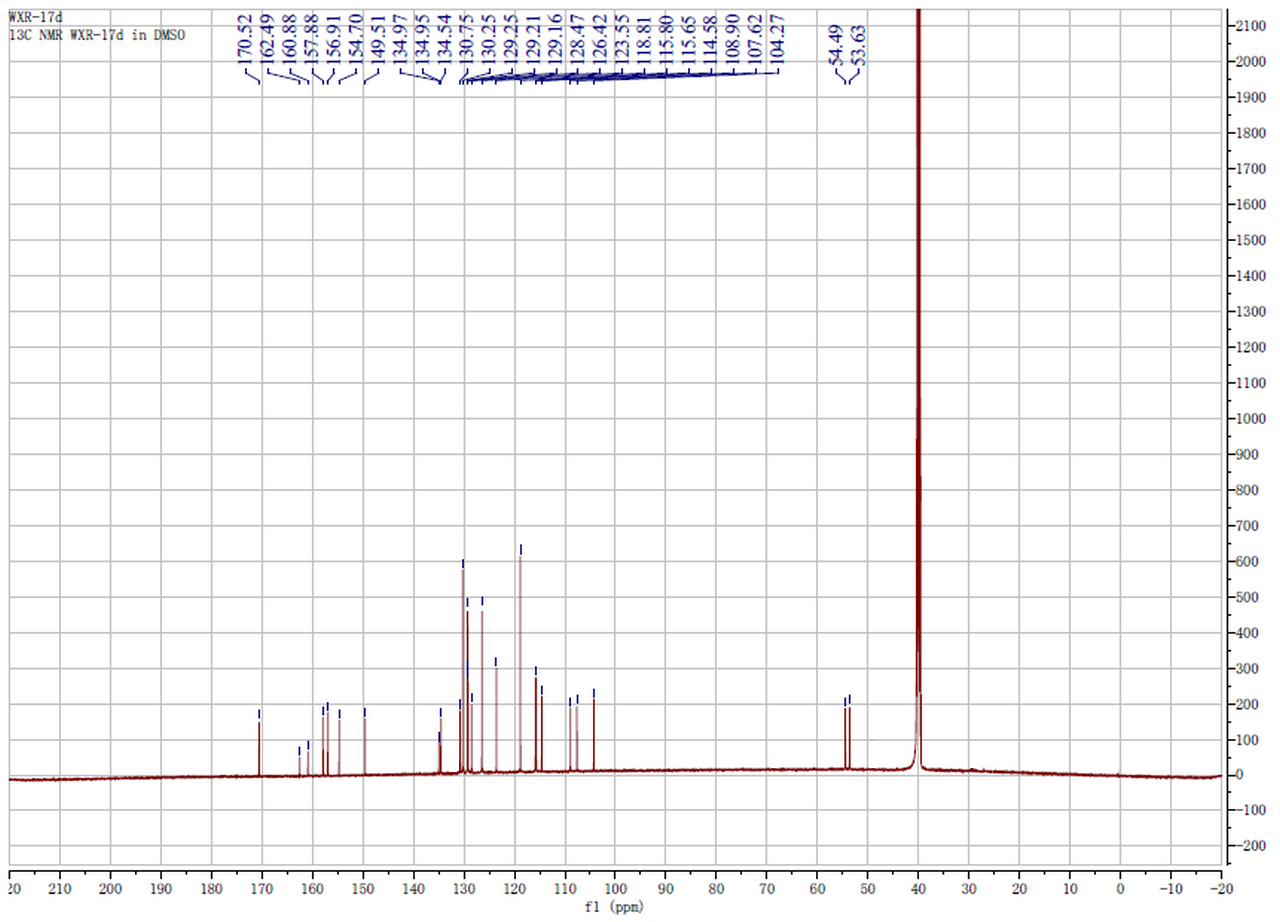

Supplement: Supplementary file 1 [file molecules-22-01925-s001.zip › Molecules-238010--13C NMR/17d.png]

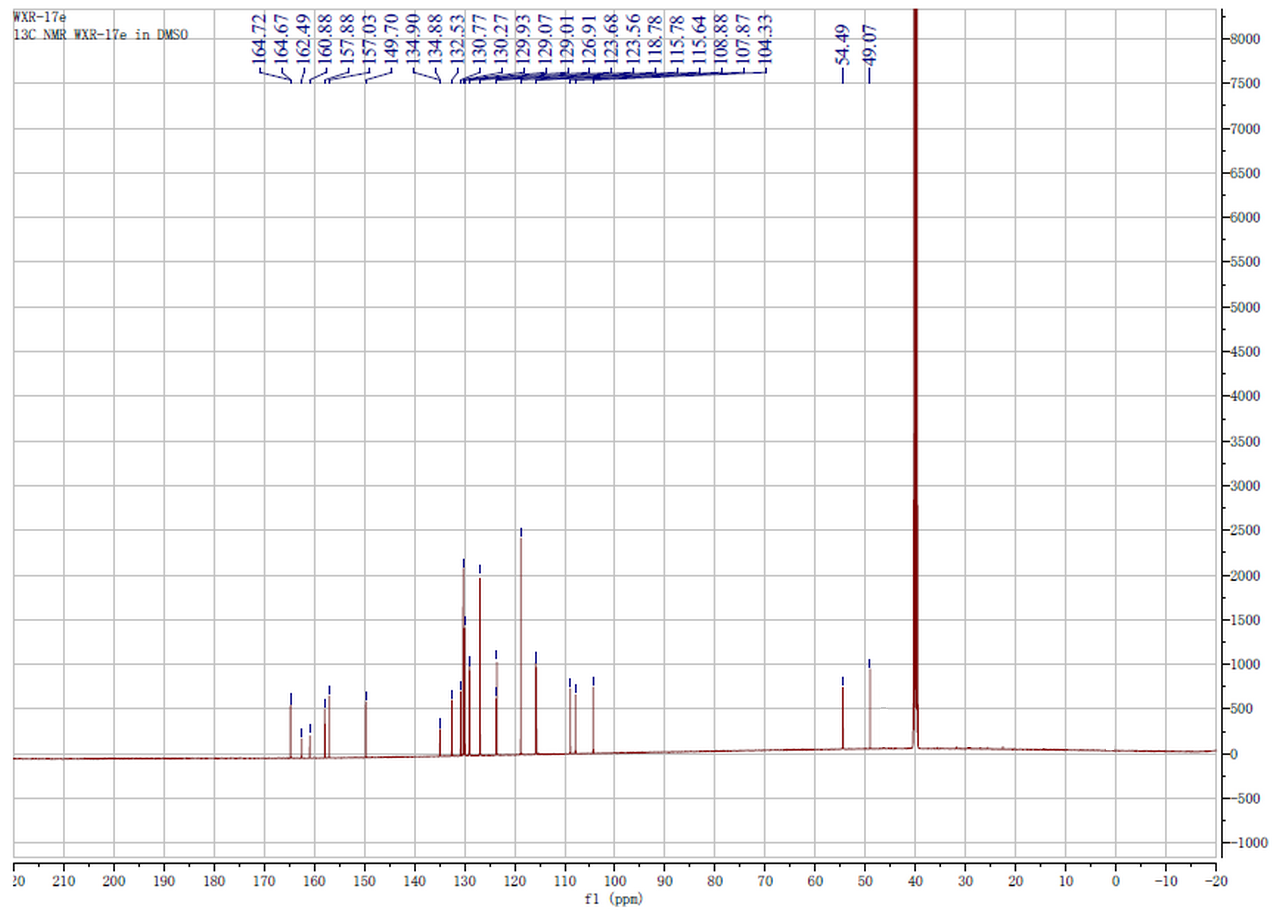

Supplement: Supplementary file 1 [file molecules-22-01925-s001.zip › Molecules-238010--13C NMR/17e.png]

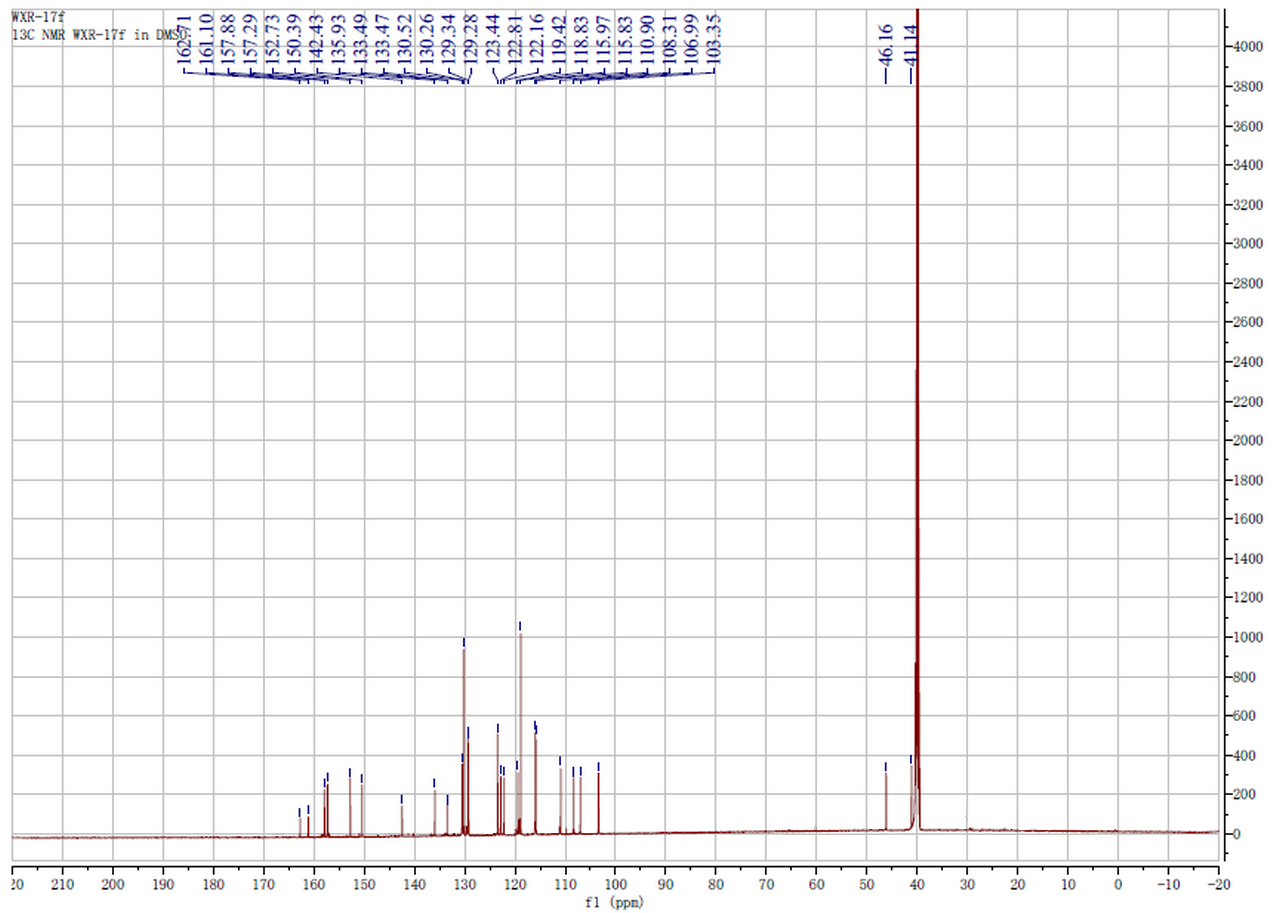

Supplement: Supplementary file 1 [file molecules-22-01925-s001.zip › Molecules-238010--13C NMR/17f.png]

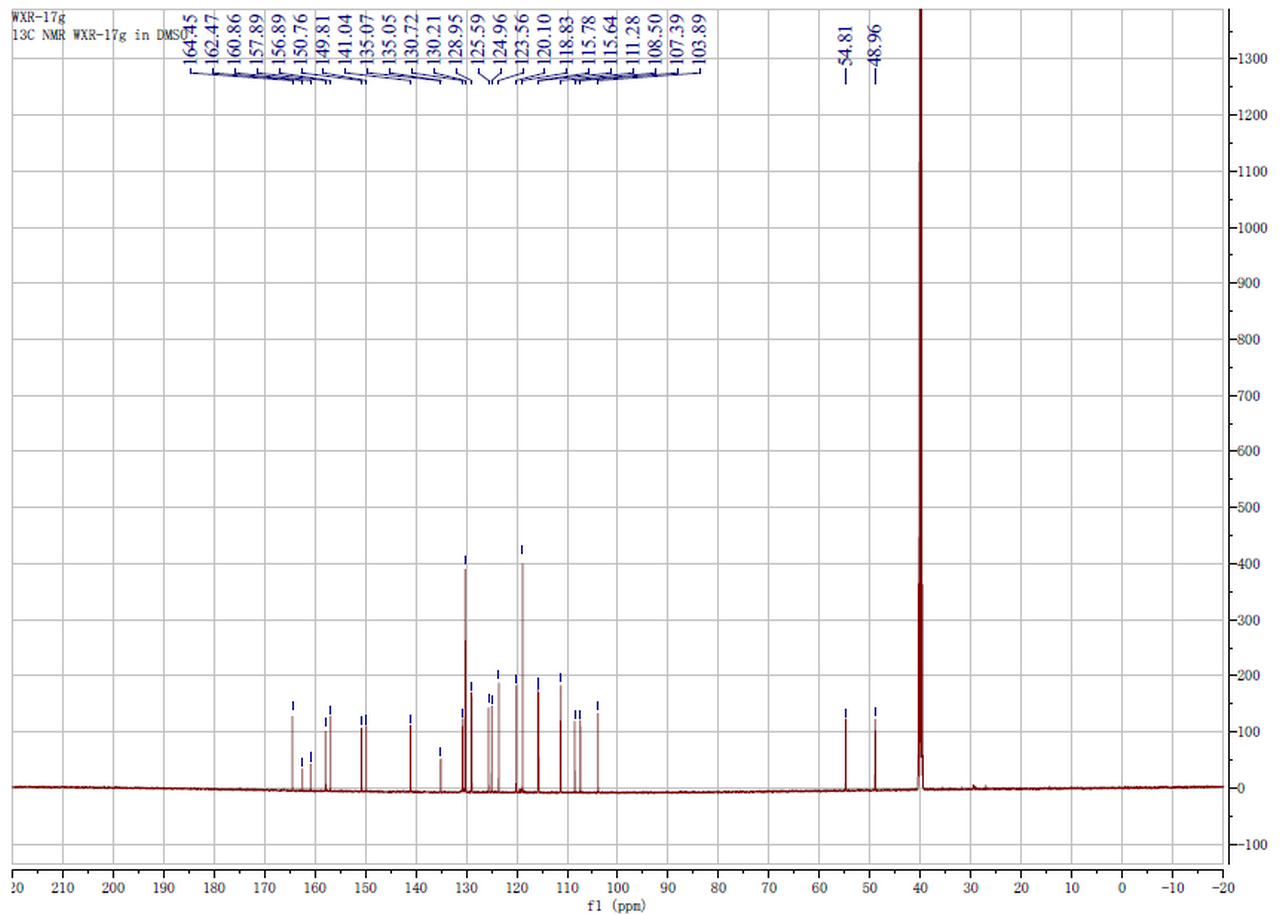

Supplement: Supplementary file 1 [file molecules-22-01925-s001.zip › Molecules-238010--13C NMR/17g.png]

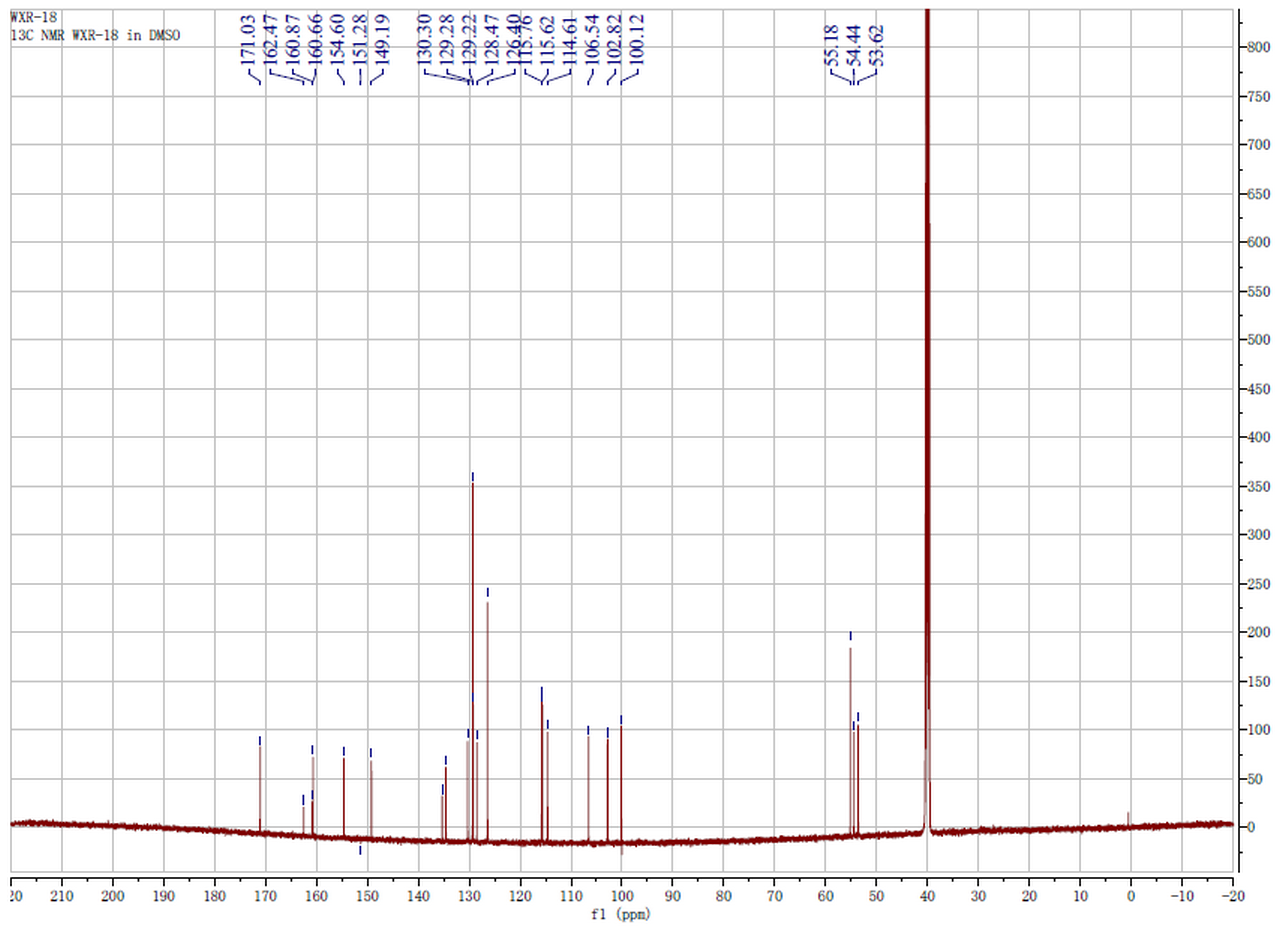

Supplement: Supplementary file 1 [file molecules-22-01925-s001.zip › Molecules-238010--13C NMR/21.png]

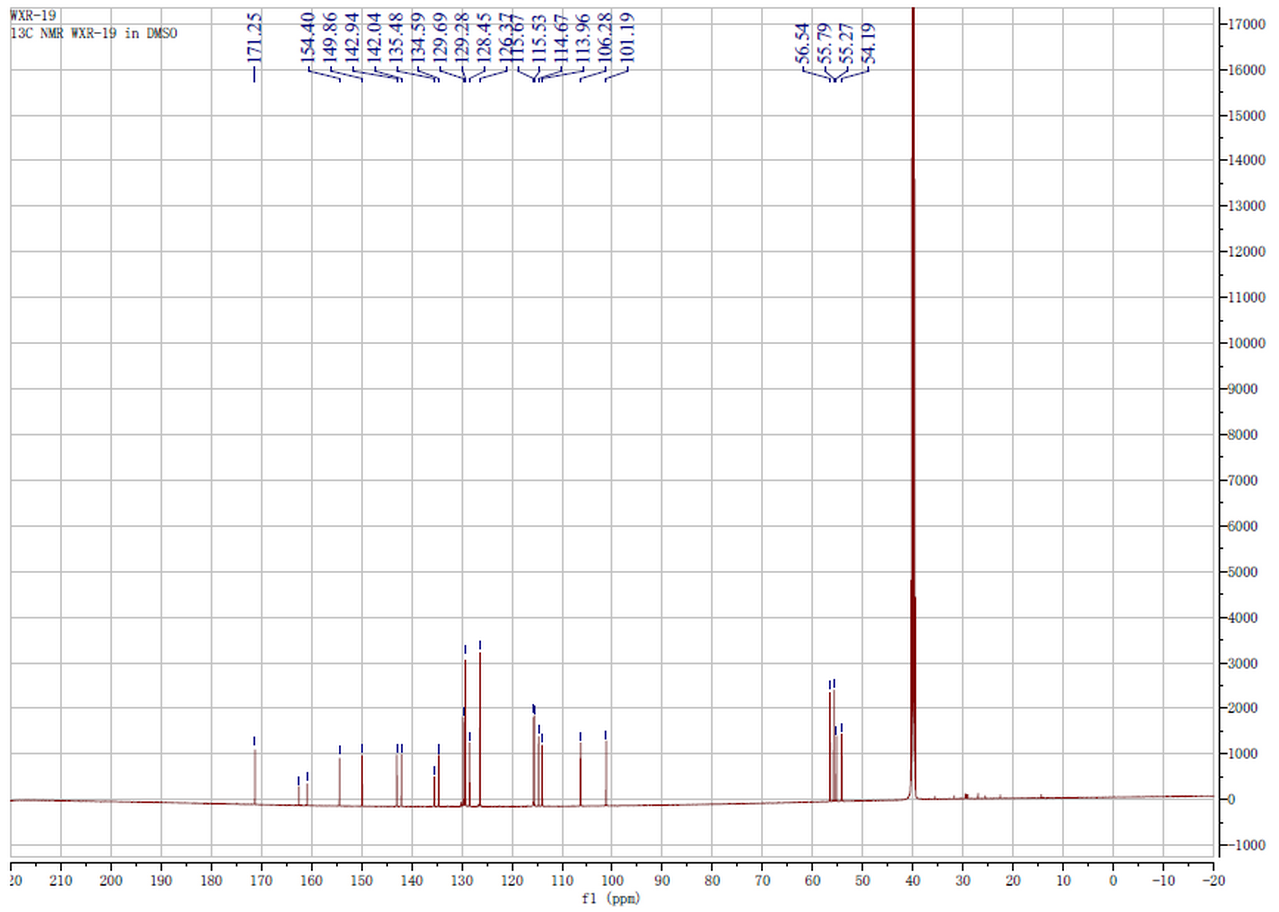

Supplement: Supplementary file 1 [file molecules-22-01925-s001.zip › Molecules-238010--13C NMR/22.png]

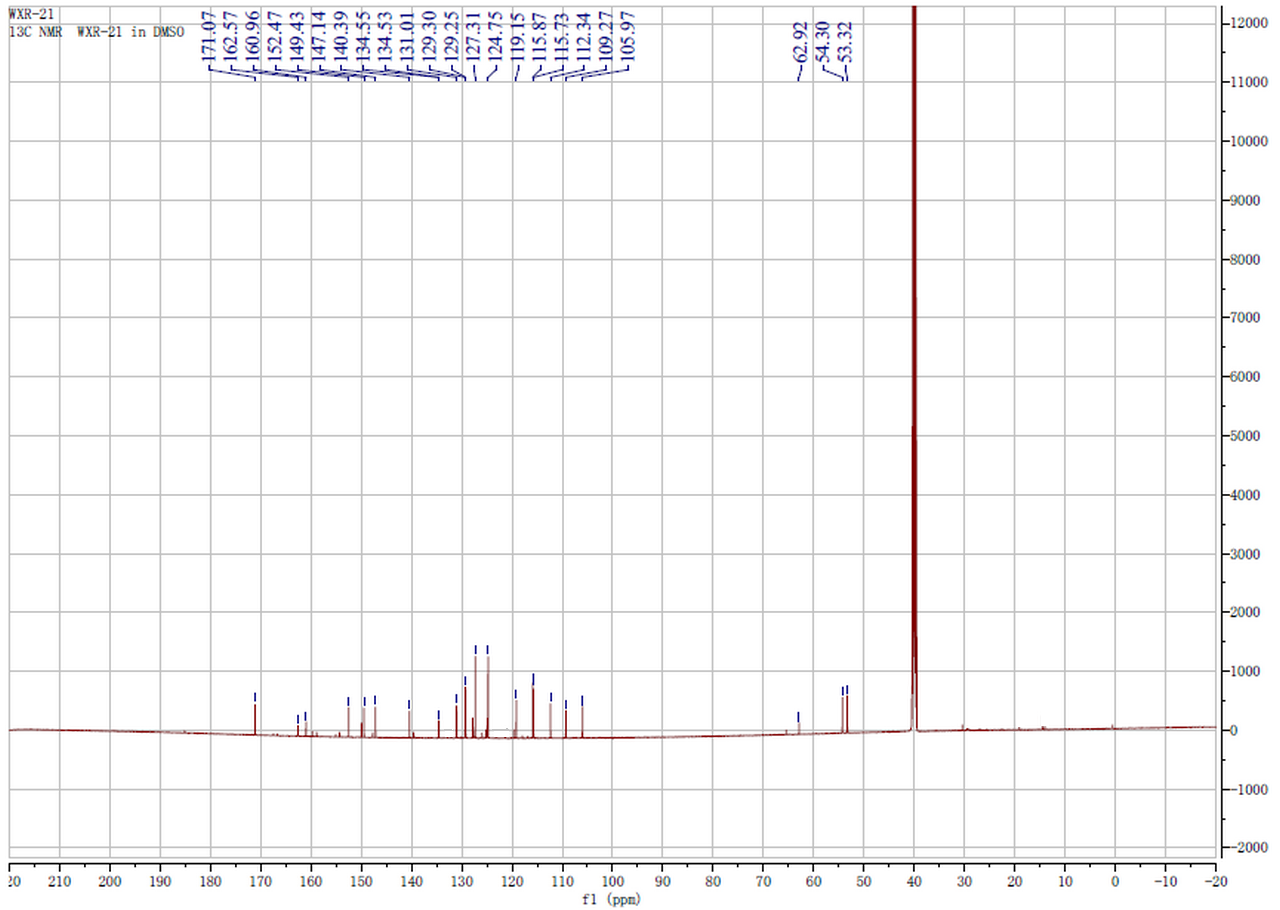

Supplement: Supplementary file 1 [file molecules-22-01925-s001.zip › Molecules-238010--13C NMR/23.png]

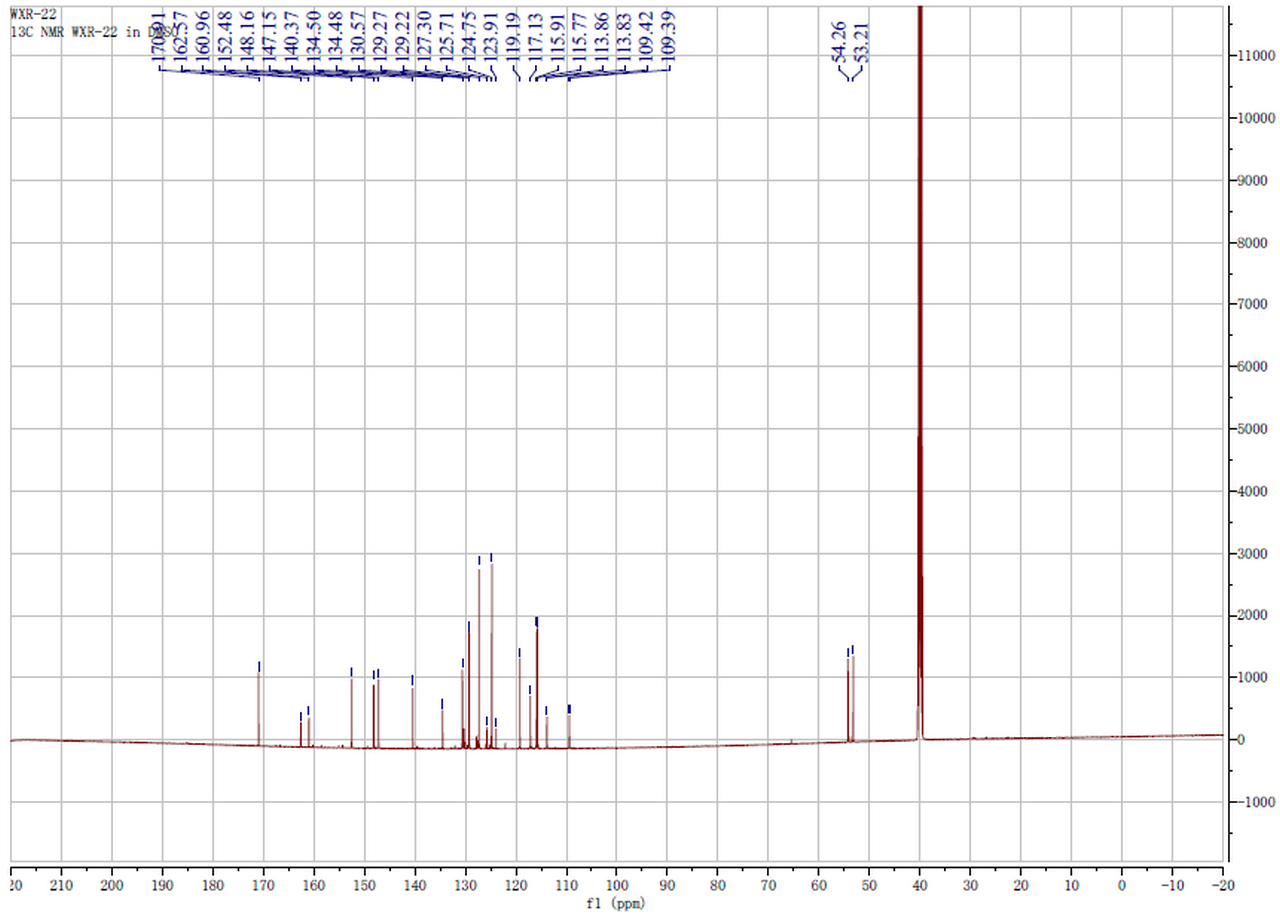

Supplement: Supplementary file 1 [file molecules-22-01925-s001.zip › Molecules-238010--13C NMR/24.png]

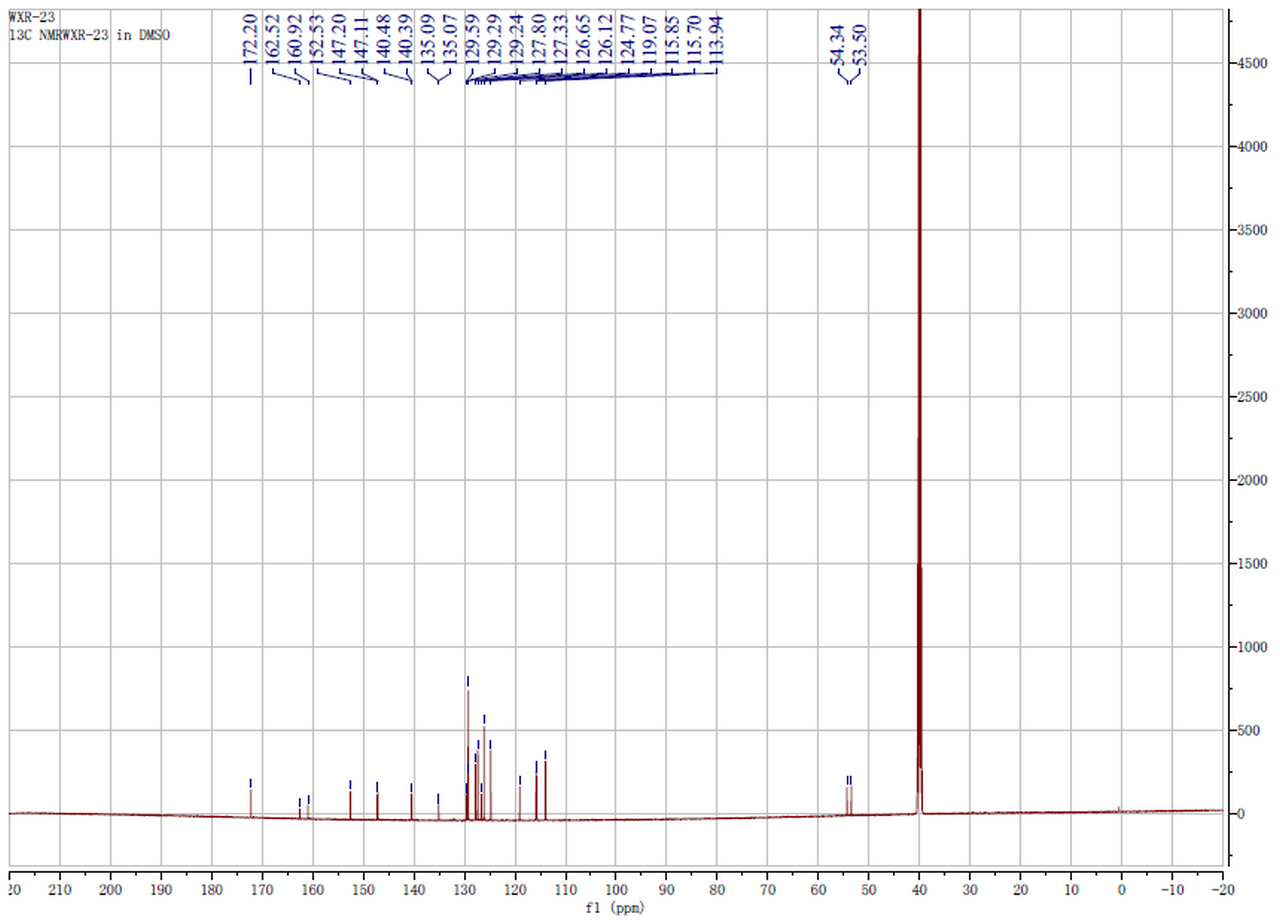

Supplement: Supplementary file 1 [file molecules-22-01925-s001.zip › Molecules-238010--13C NMR/25.png]

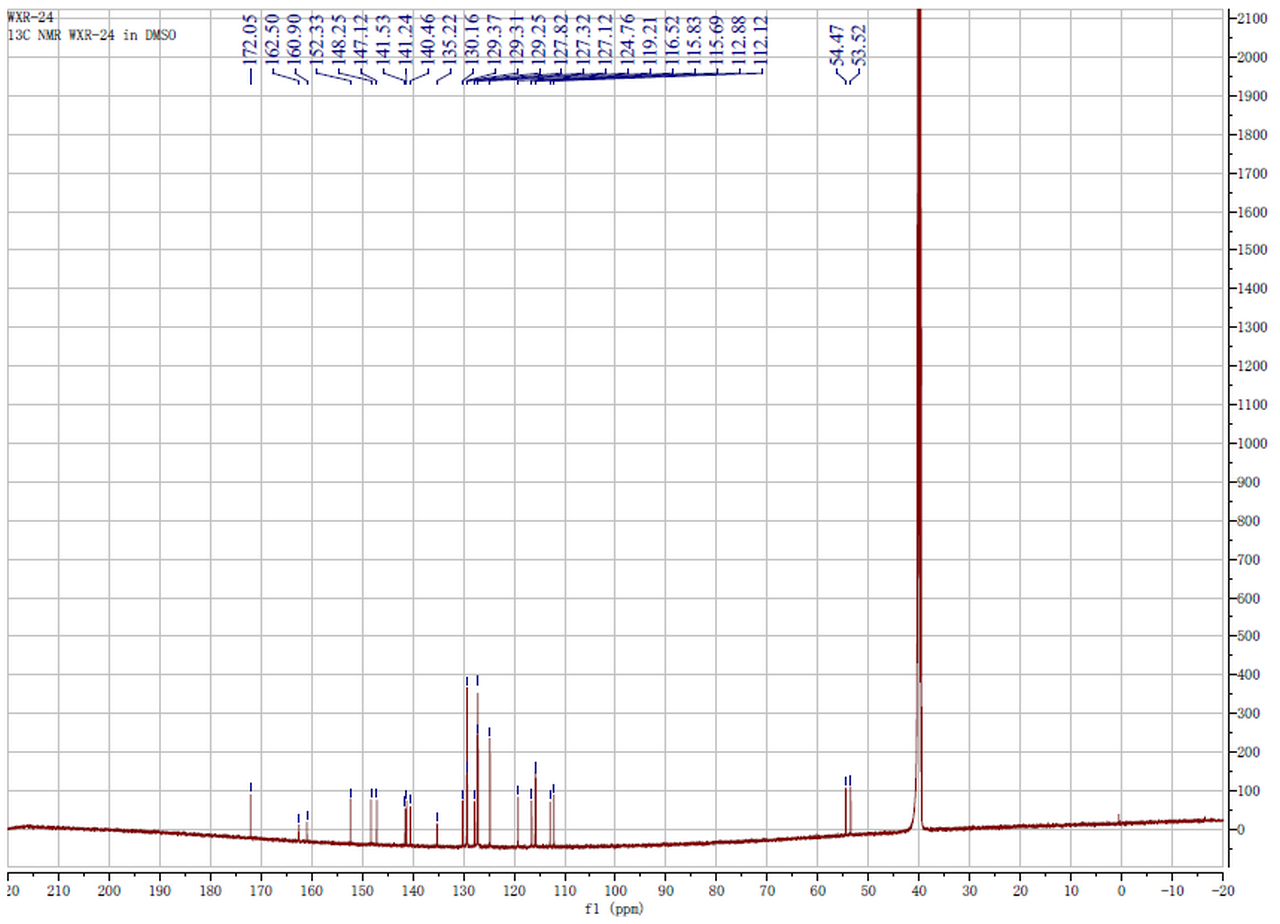

Supplement: Supplementary file 1 [file molecules-22-01925-s001.zip › Molecules-238010--13C NMR/26.png]

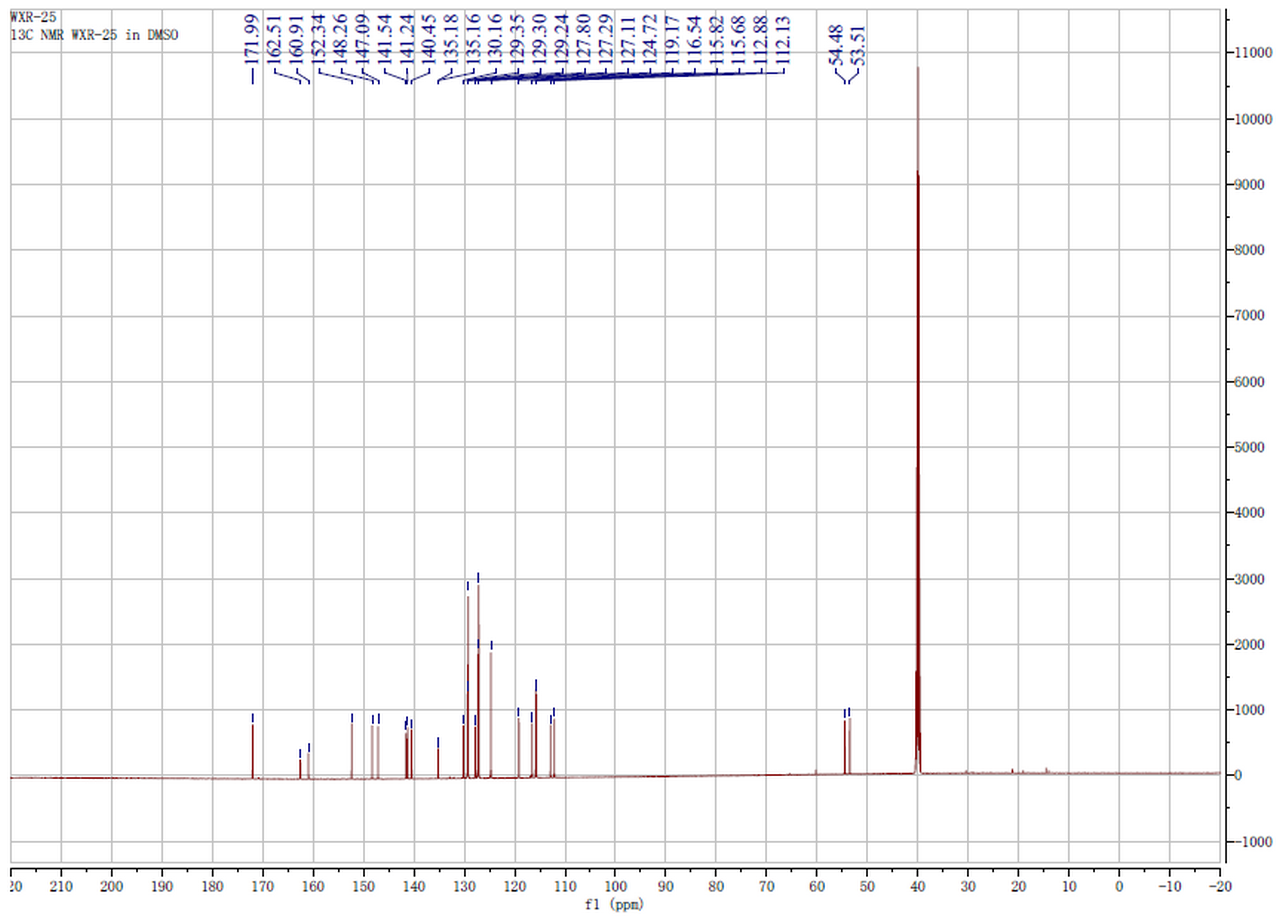

Supplement: Supplementary file 1 [file molecules-22-01925-s001.zip › Molecules-238010--13C NMR/27.png]

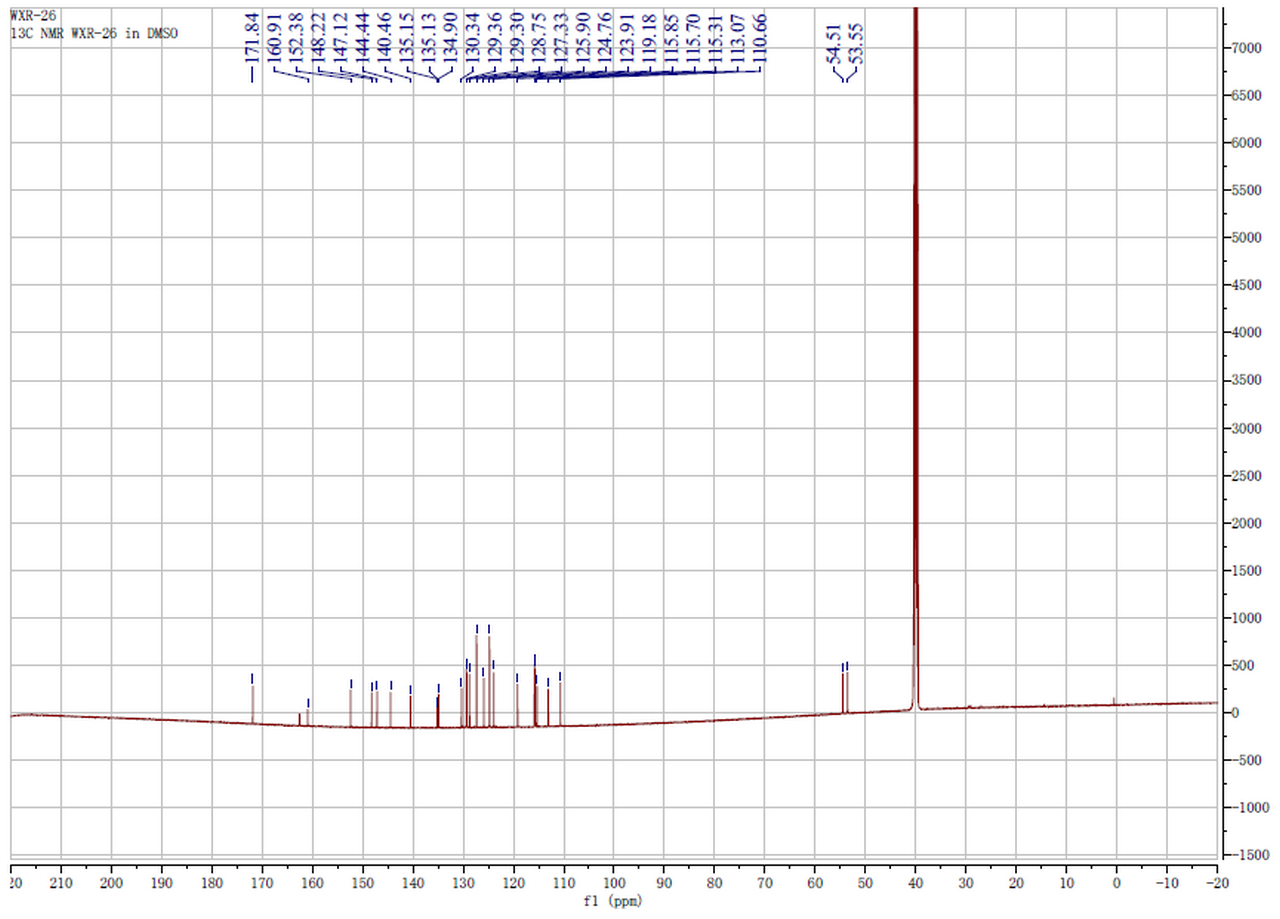

Supplement: Supplementary file 1 [file molecules-22-01925-s001.zip › Molecules-238010--13C NMR/28.png]

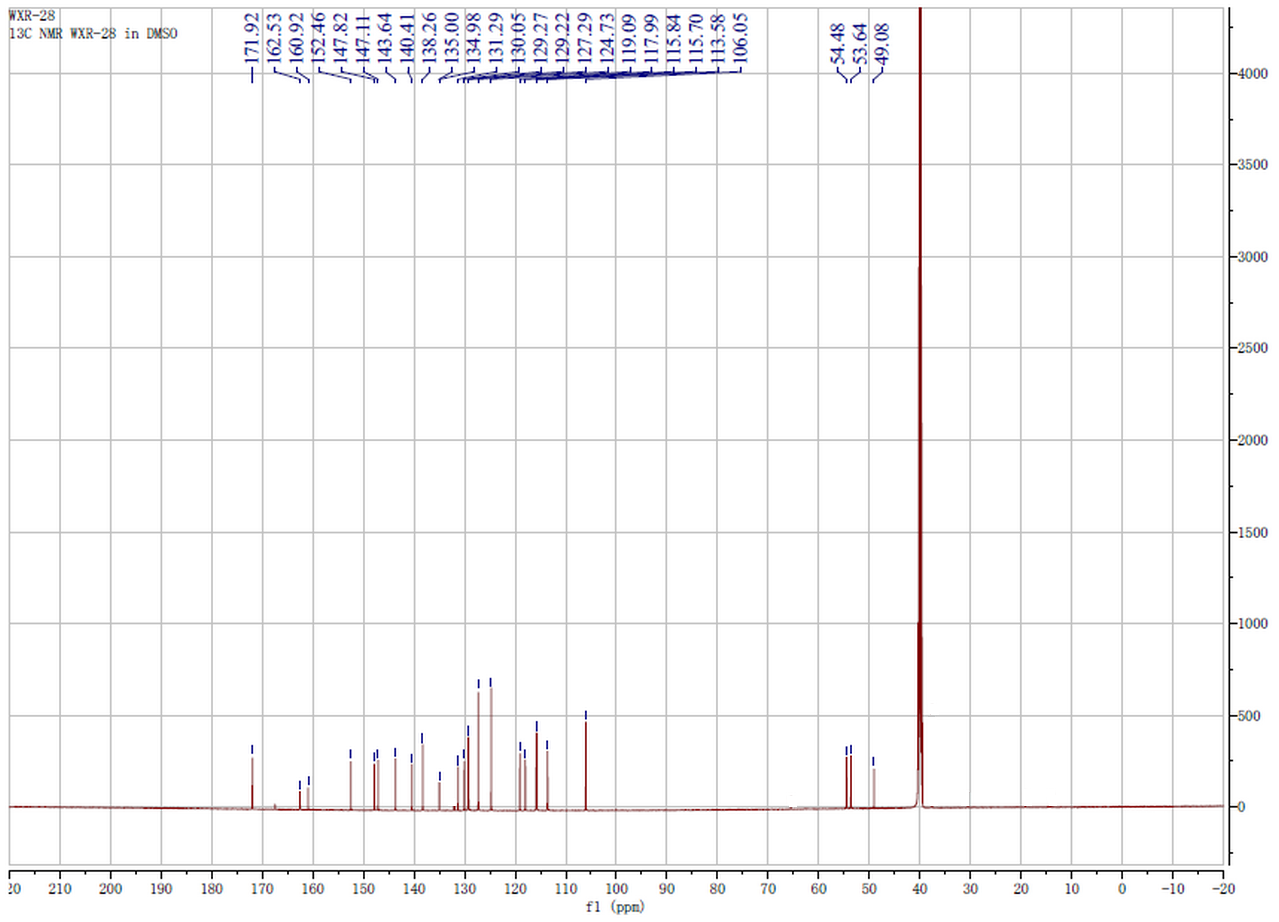

Supplement: Supplementary file 1 [file molecules-22-01925-s001.zip › Molecules-238010--13C NMR/29.png]

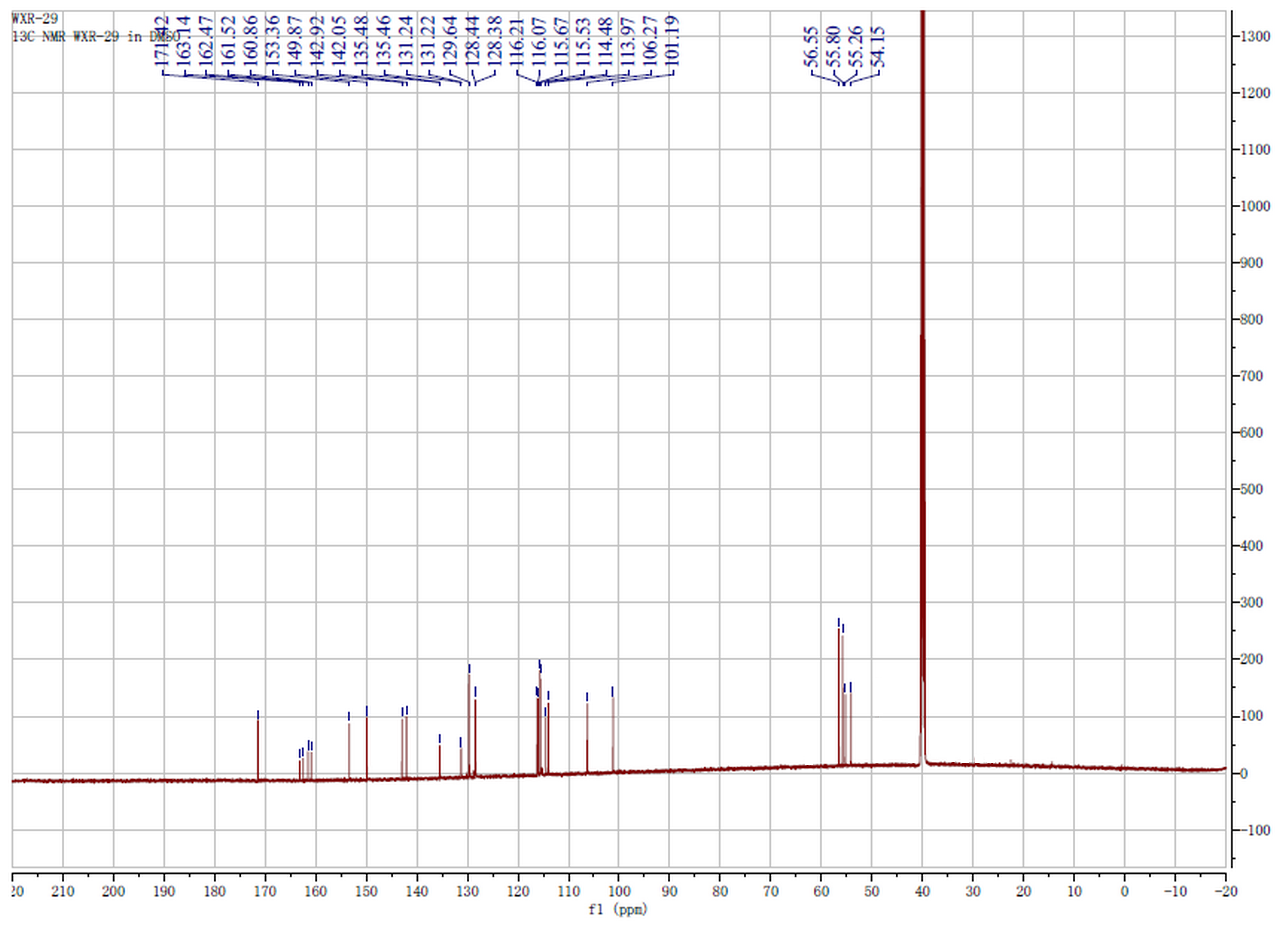

Supplement: Supplementary file 1 [file molecules-22-01925-s001.zip › Molecules-238010--13C NMR/30.png]

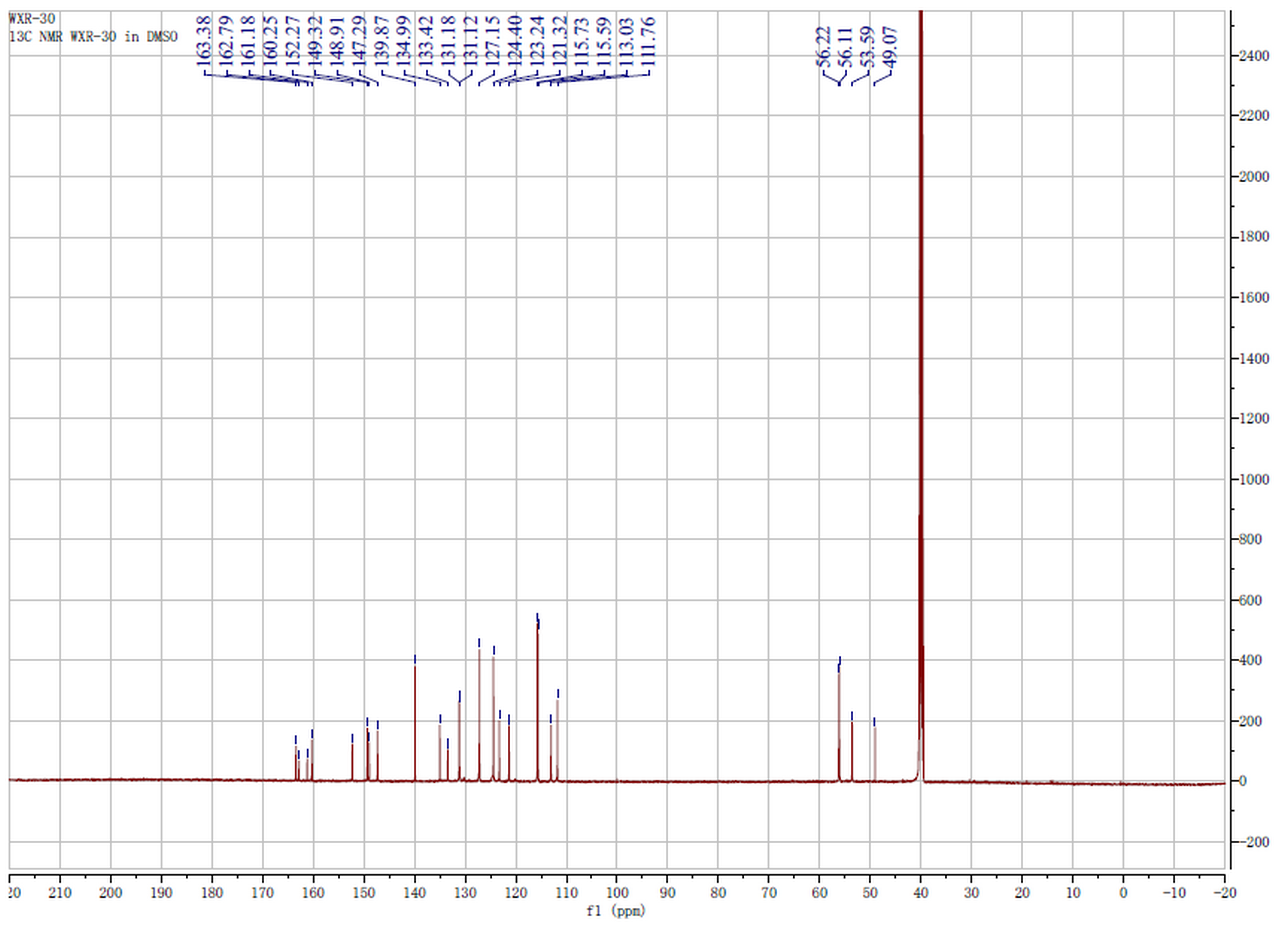

Supplement: Supplementary file 1 [file molecules-22-01925-s001.zip › Molecules-238010--13C NMR/31.png]

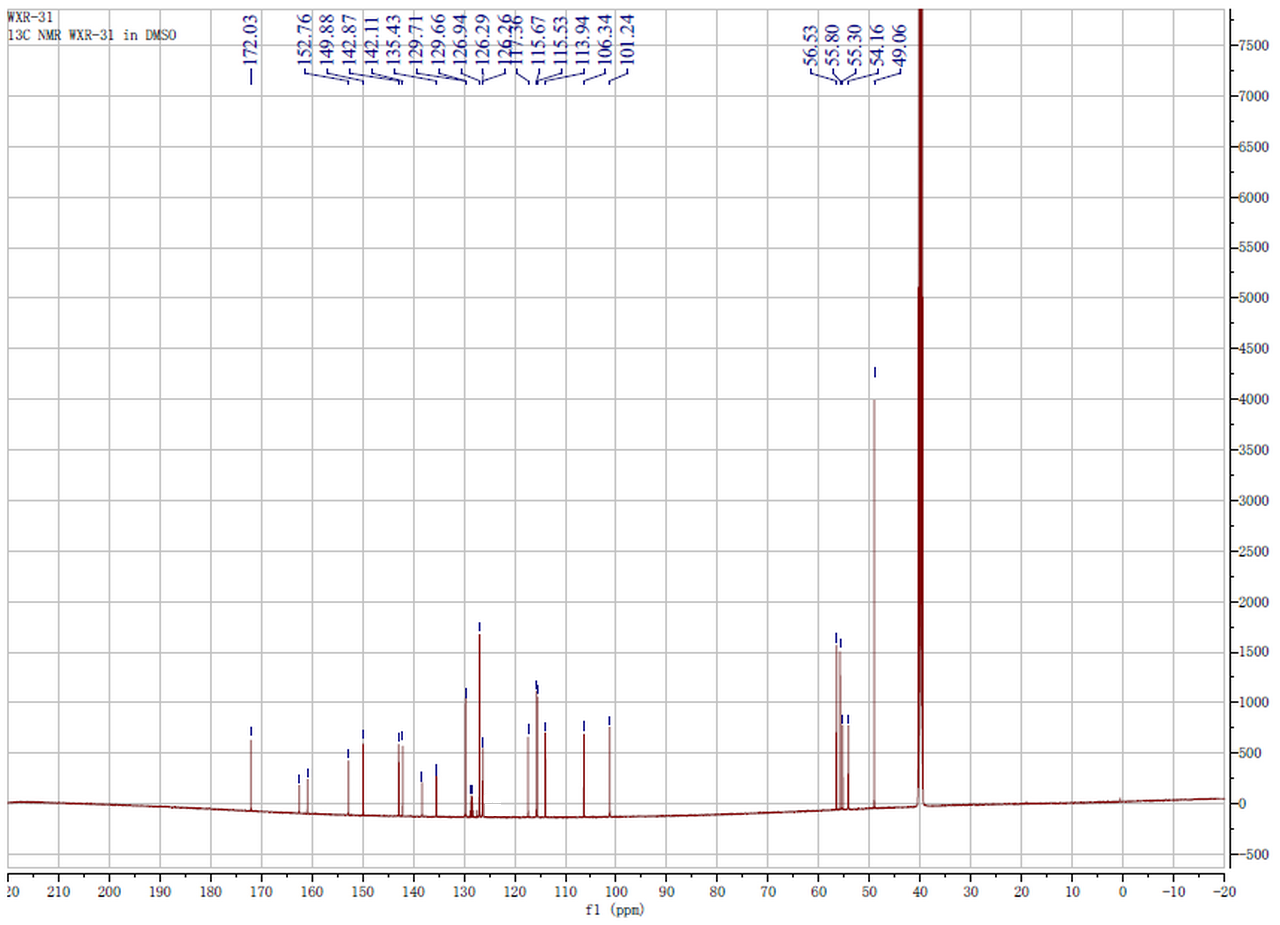

Supplement: Supplementary file 1 [file molecules-22-01925-s001.zip › Molecules-238010--13C NMR/32.png]

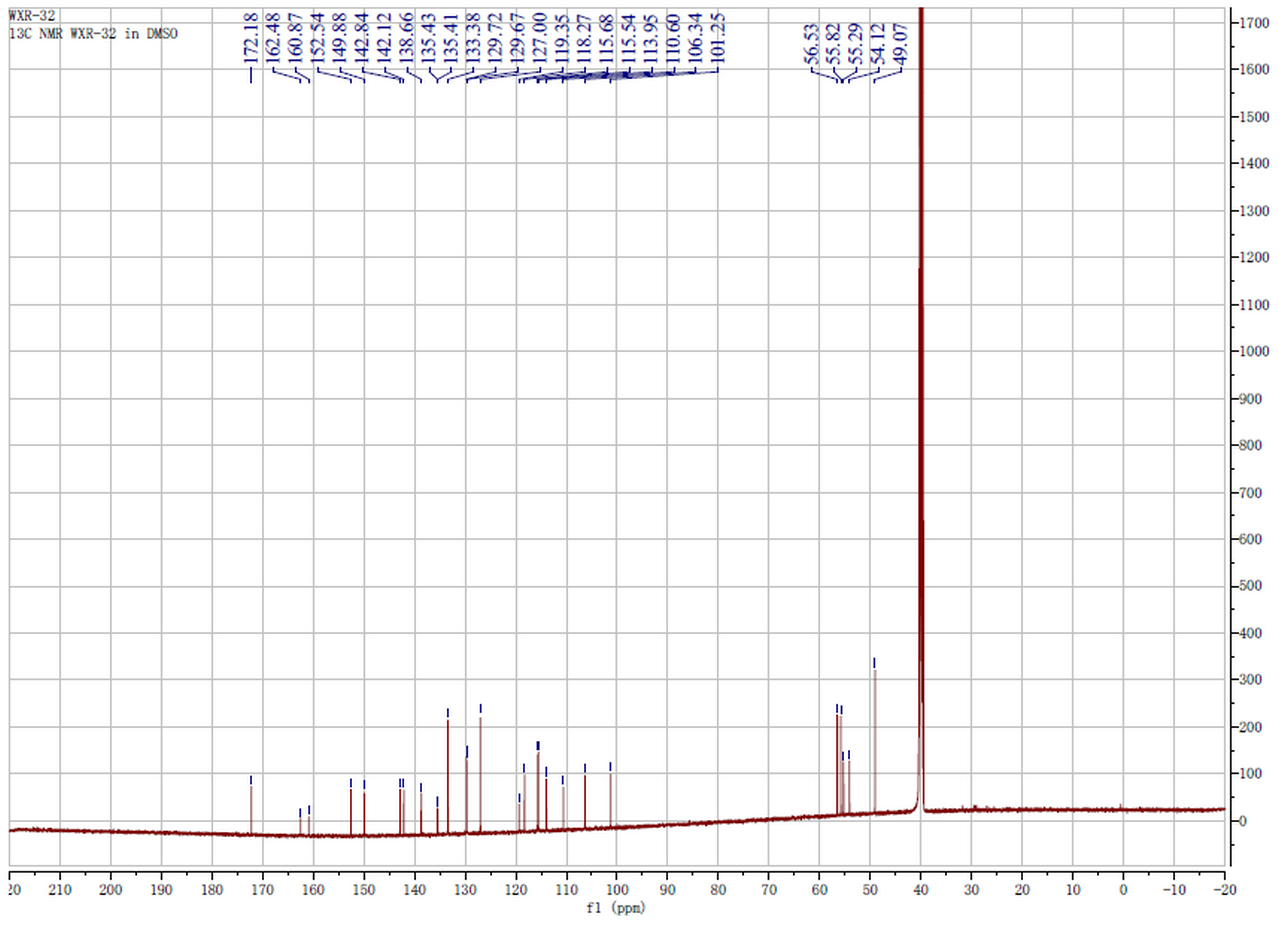

Supplement: Supplementary file 1 [file molecules-22-01925-s001.zip › Molecules-238010--13C NMR/33.png]

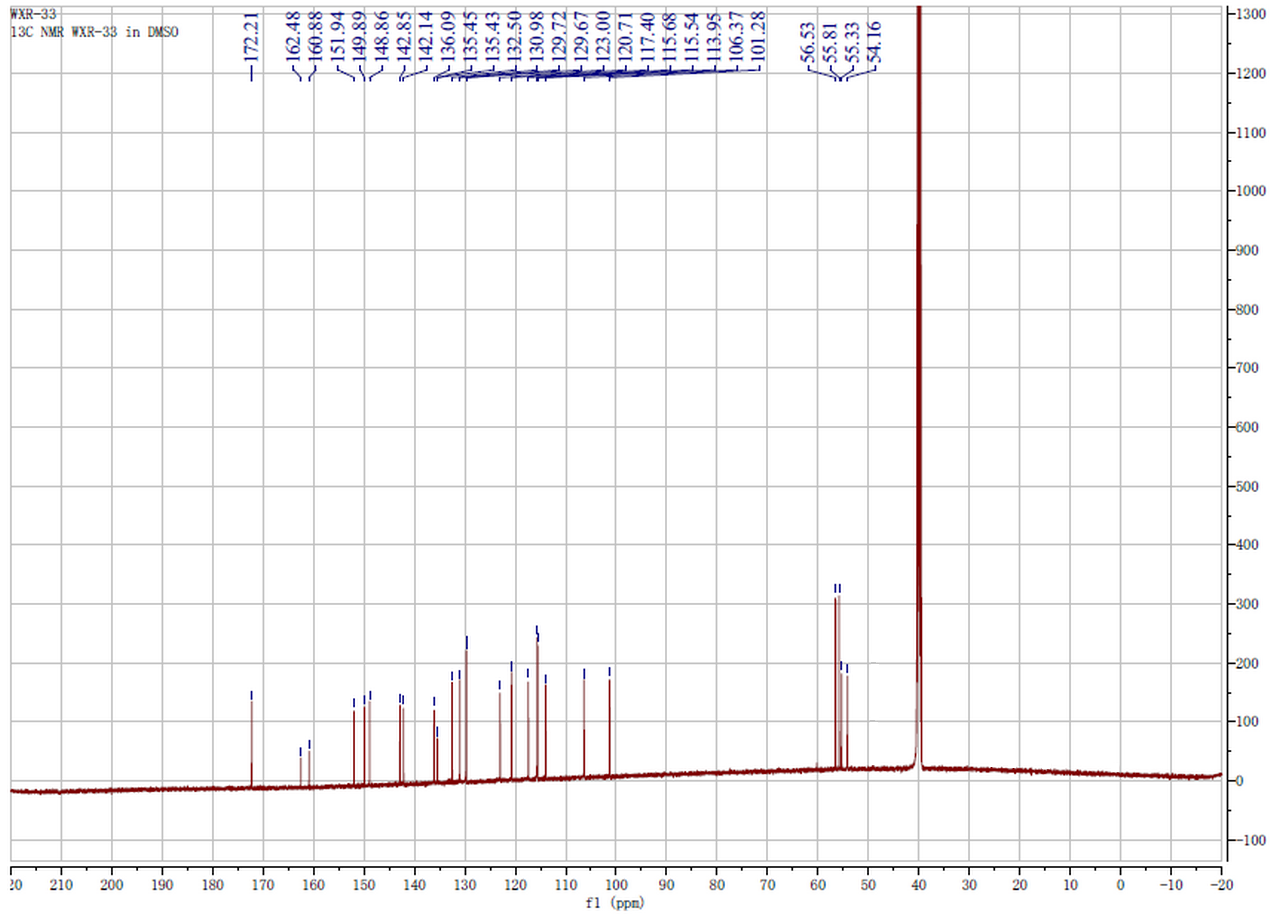

Supplement: Supplementary file 1 [file molecules-22-01925-s001.zip › Molecules-238010--13C NMR/34.png]

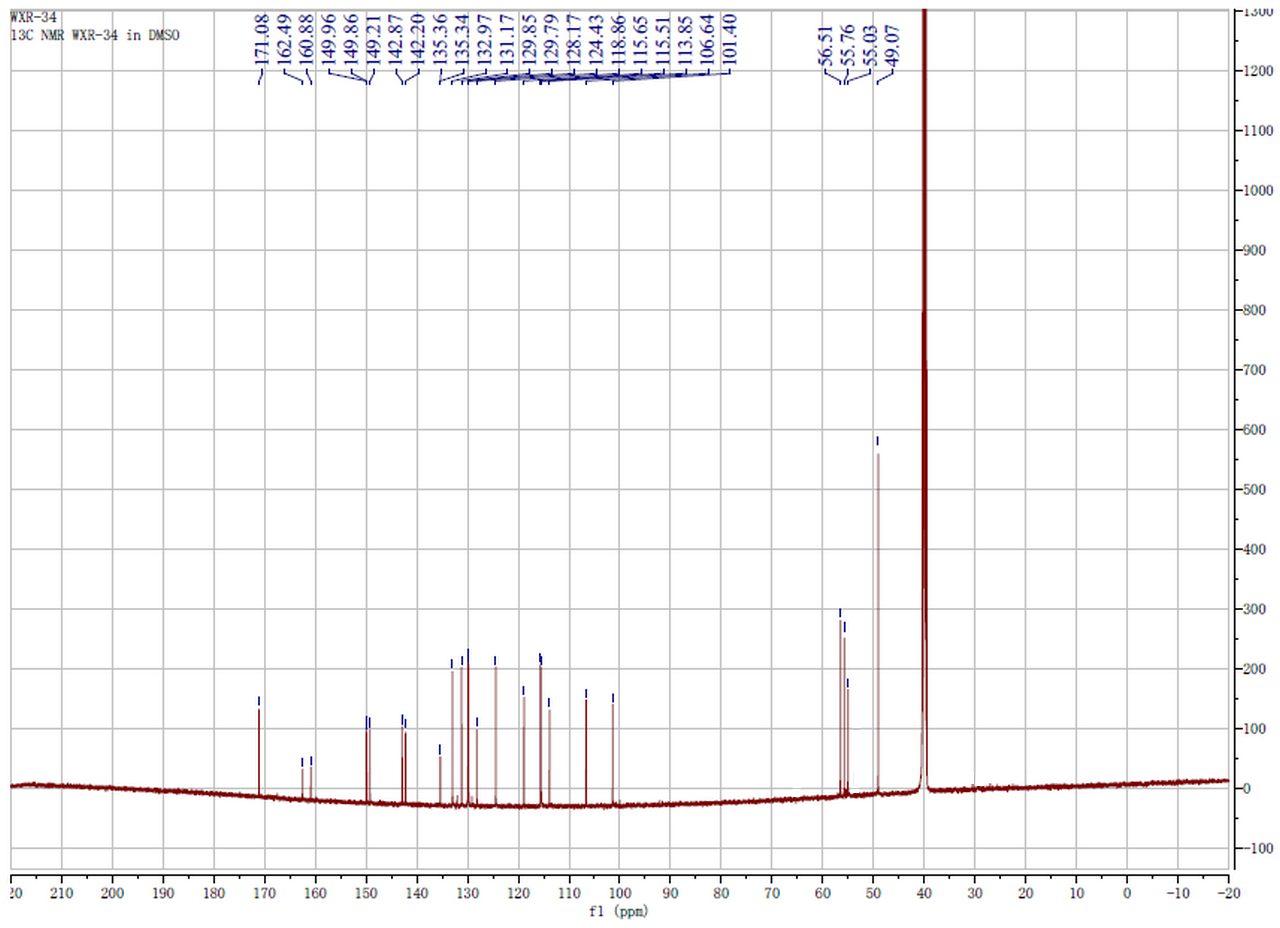

Supplement: Supplementary file 1 [file molecules-22-01925-s001.zip › Molecules-238010--13C NMR/35.png]

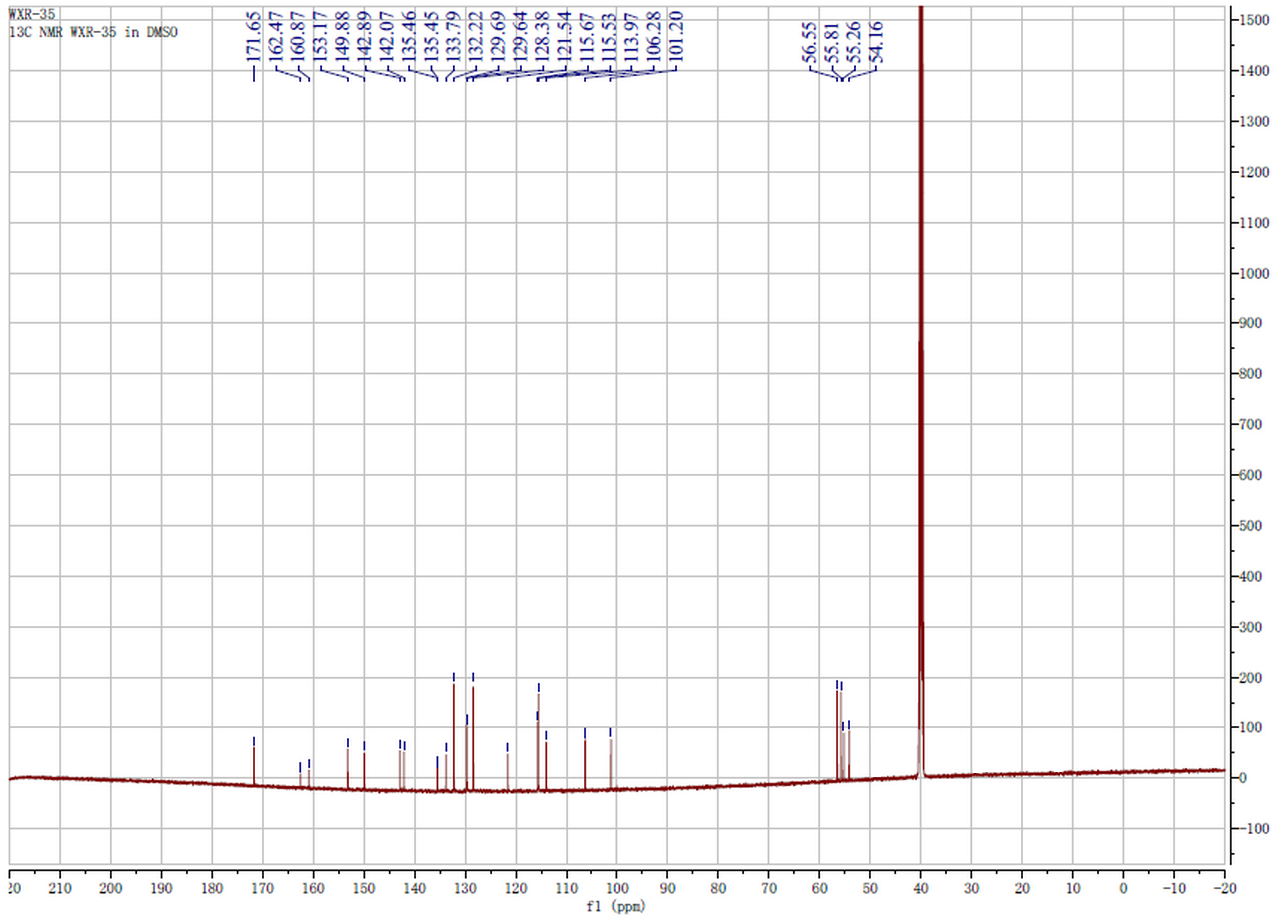

Supplement: Supplementary file 1 [file molecules-22-01925-s001.zip › Molecules-238010--13C NMR/36.png]

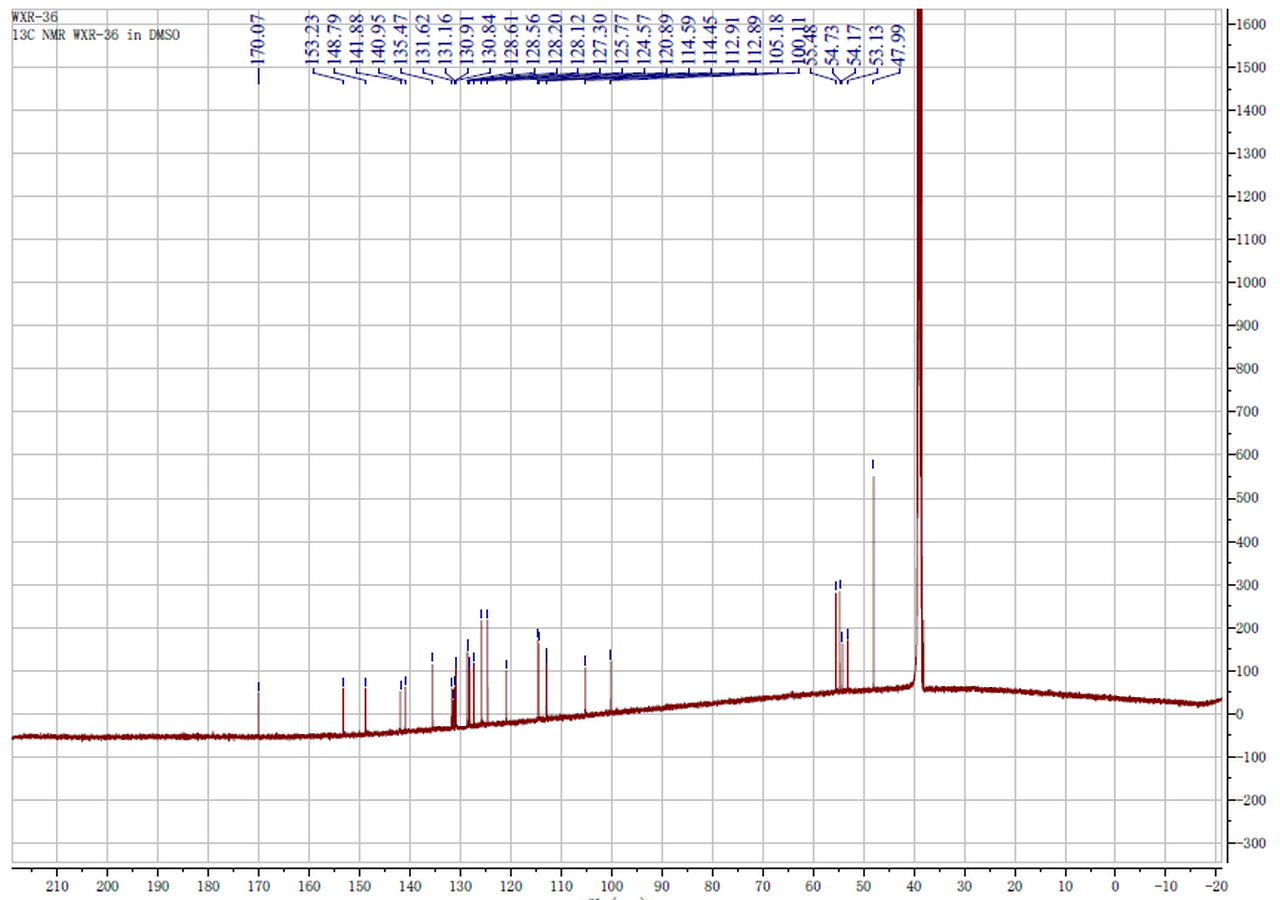

Supplement: Supplementary file 1 [file molecules-22-01925-s001.zip › Molecules-238010--13C NMR/37.png]

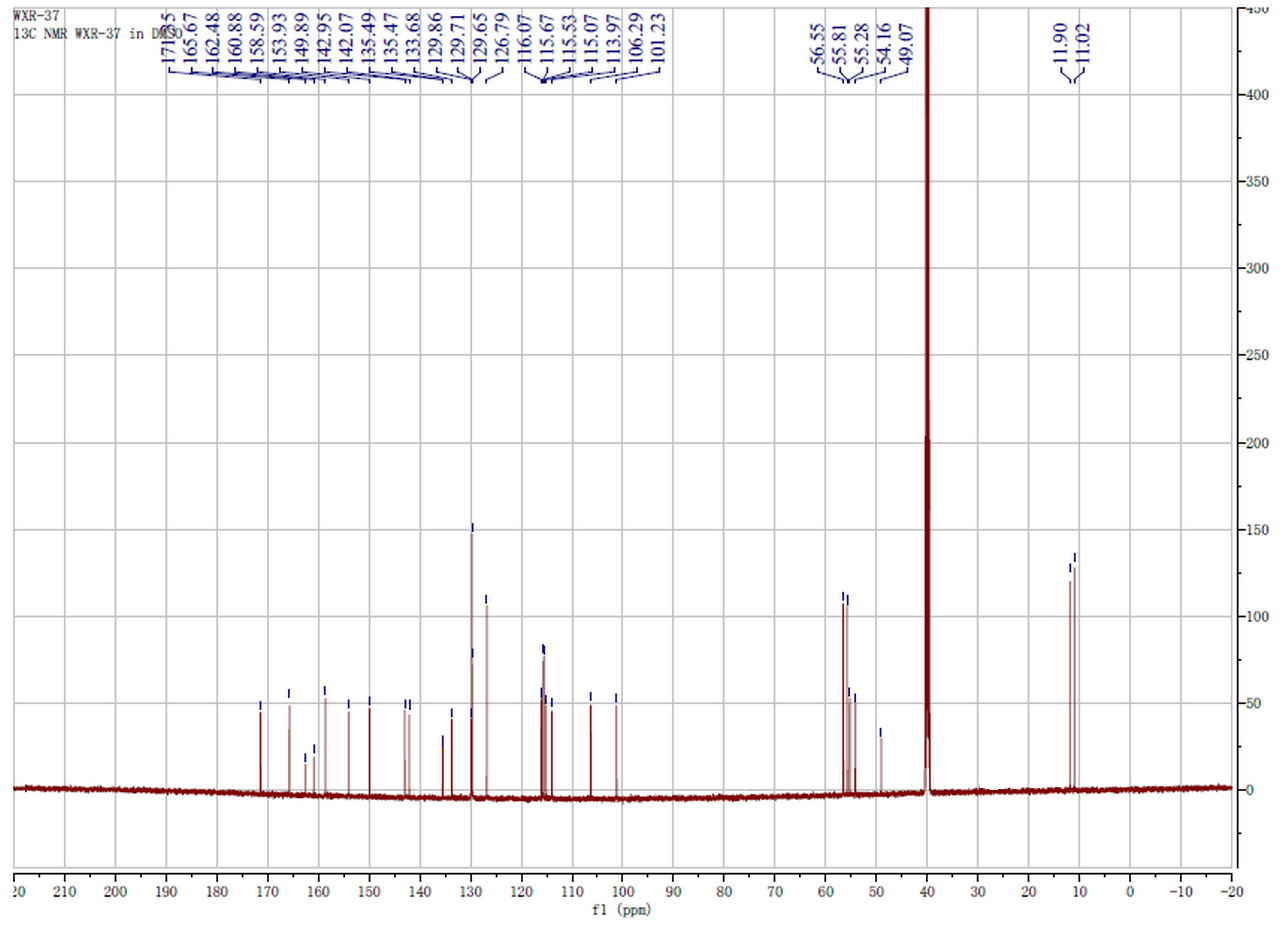

Supplement: Supplementary file 1 [file molecules-22-01925-s001.zip › Molecules-238010--13C NMR/38.png]

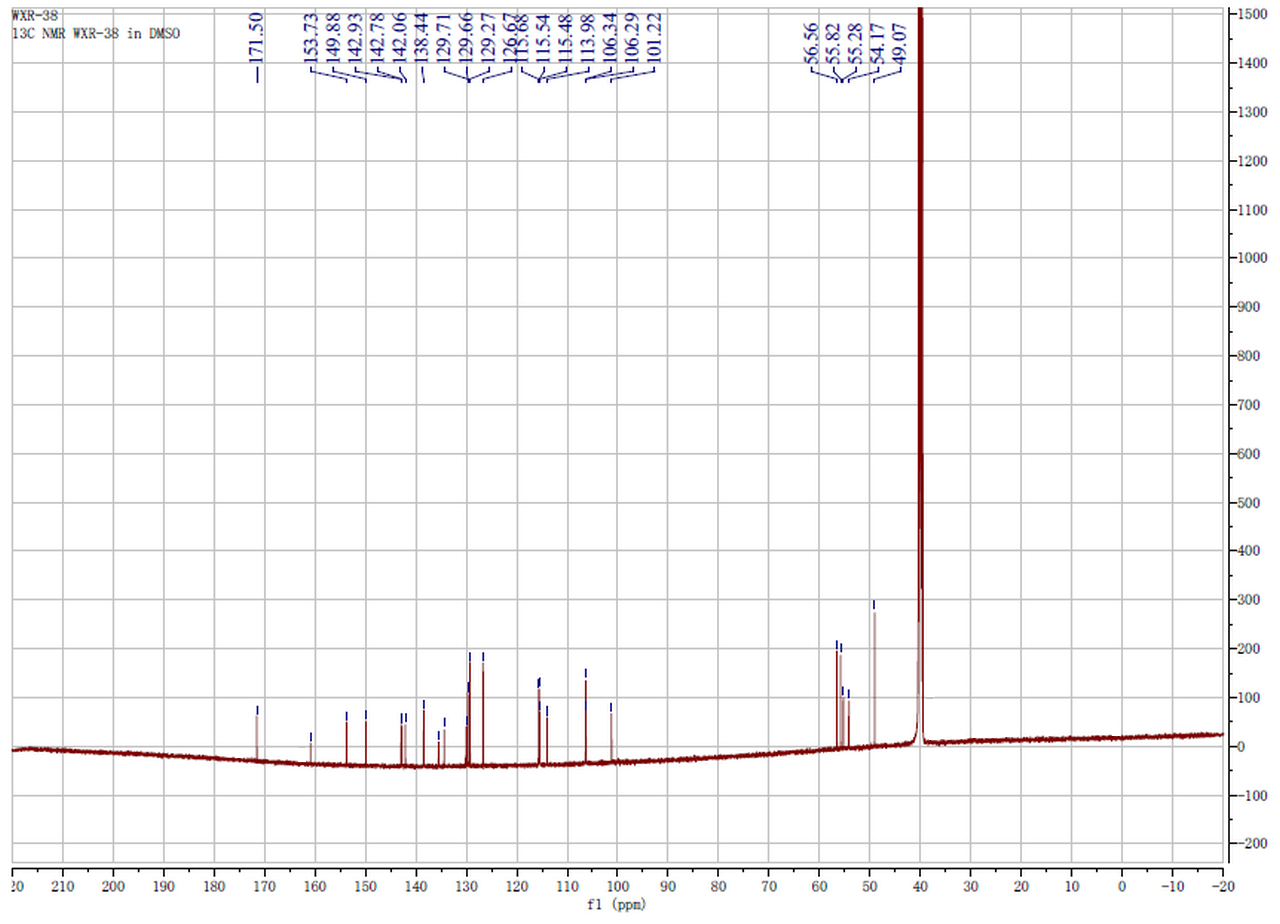

Supplement: Supplementary file 1 [file molecules-22-01925-s001.zip › Molecules-238010--13C NMR/39.png]

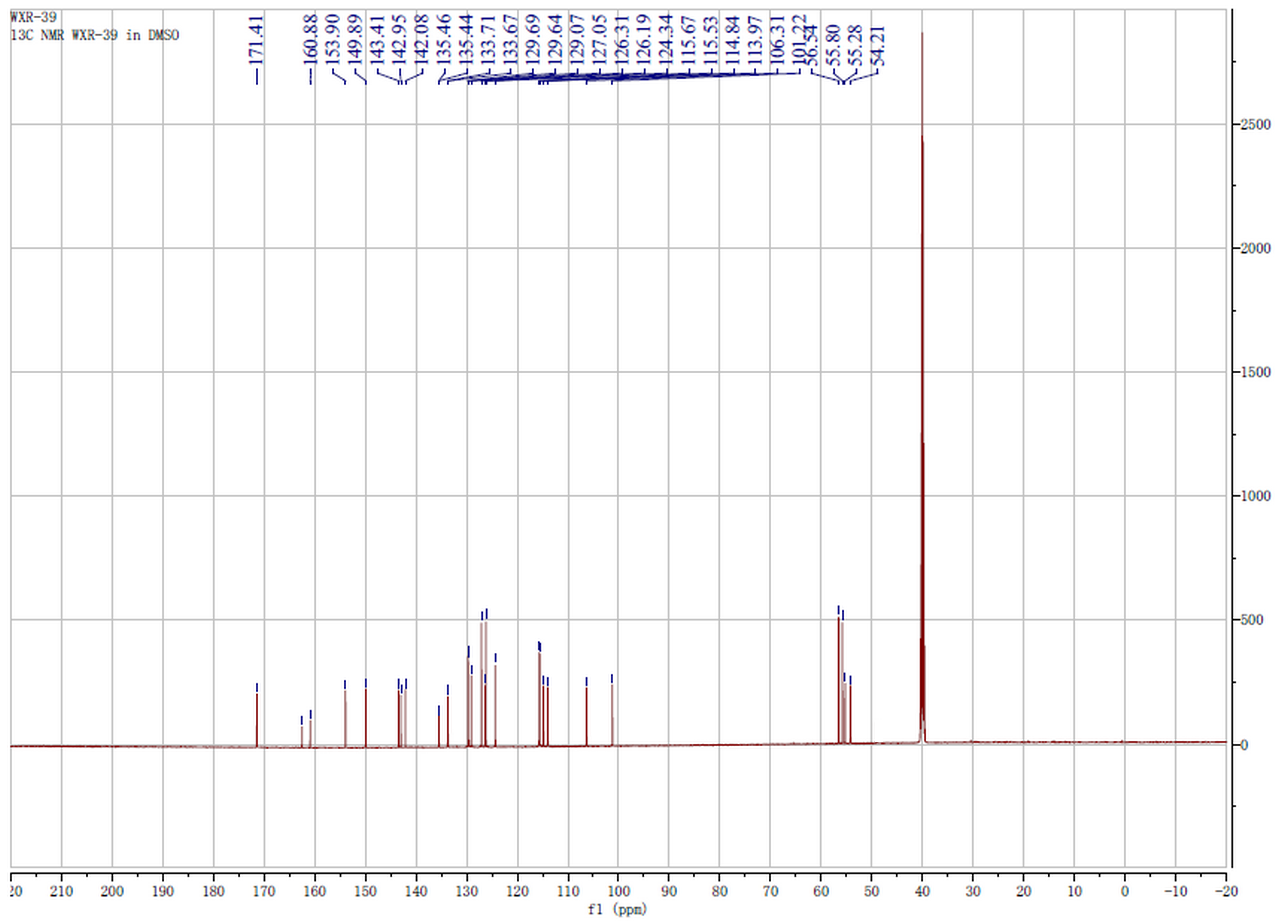

Supplement: Supplementary file 1 [file molecules-22-01925-s001.zip › Molecules-238010--13C NMR/40.png]

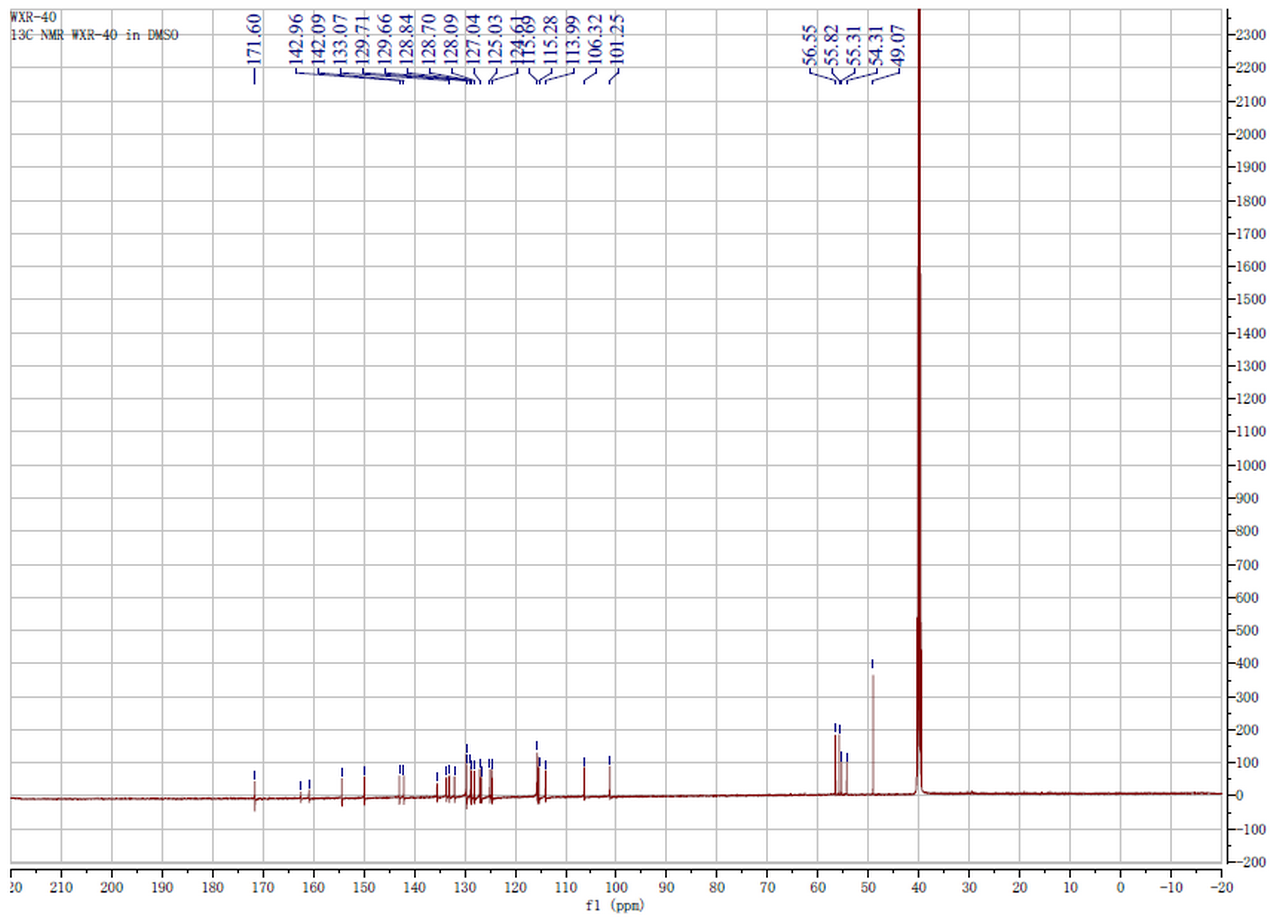

Supplement: Supplementary file 1 [file molecules-22-01925-s001.zip › Molecules-238010--13C NMR/41.png]

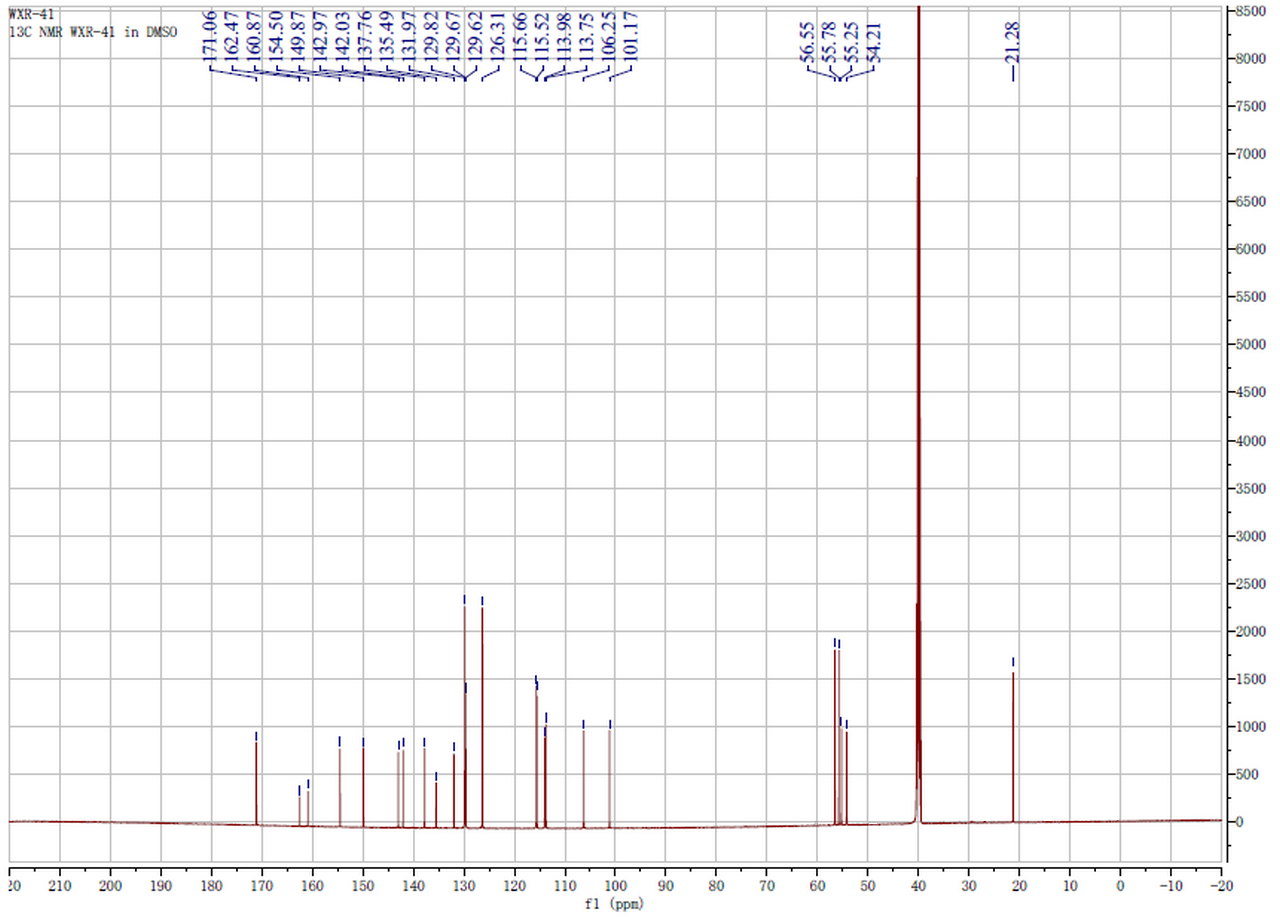

Supplement: Supplementary file 1 [file molecules-22-01925-s001.zip › Molecules-238010--13C NMR/42.png]

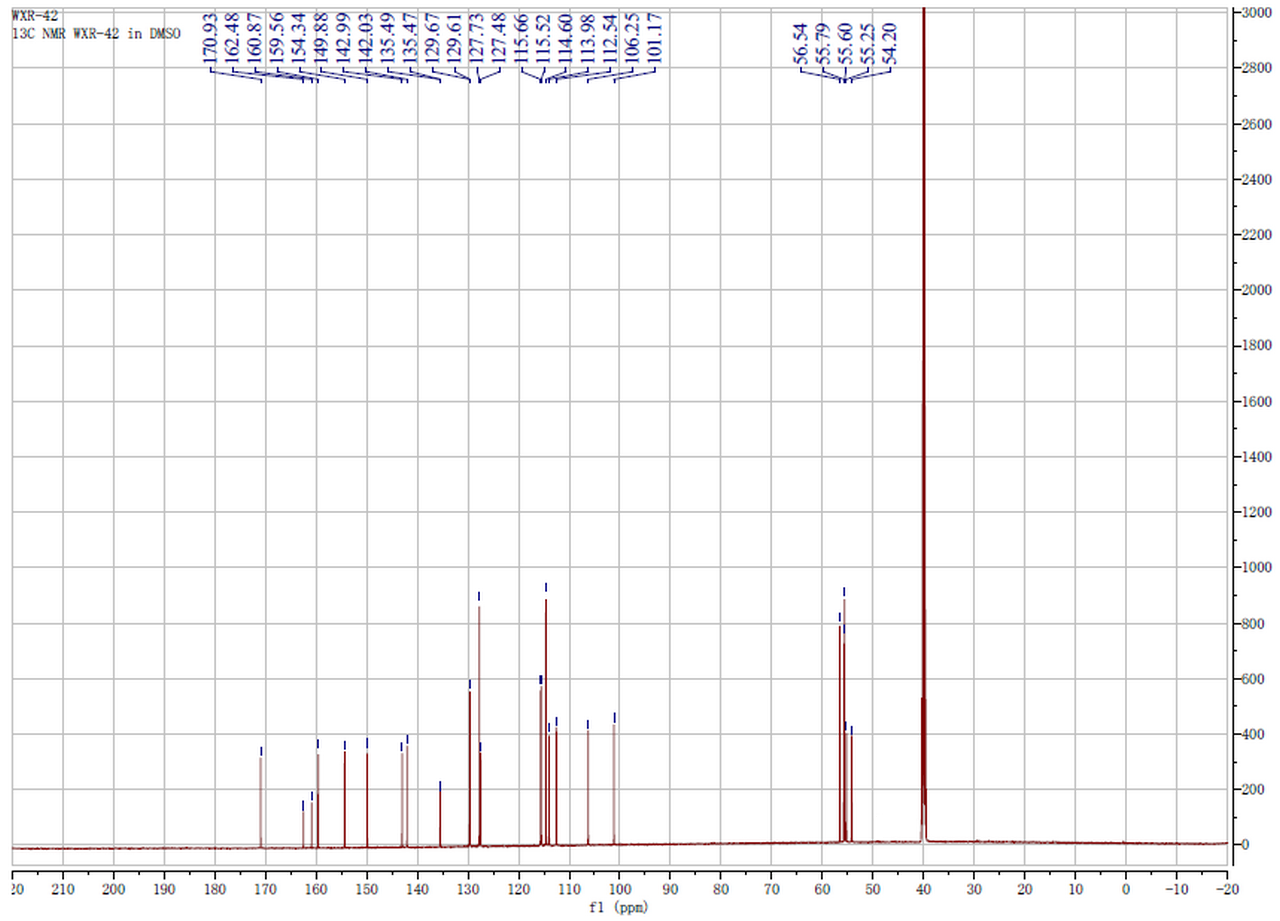

Supplement: Supplementary file 1 [file molecules-22-01925-s001.zip › Molecules-238010--13C NMR/43.png]

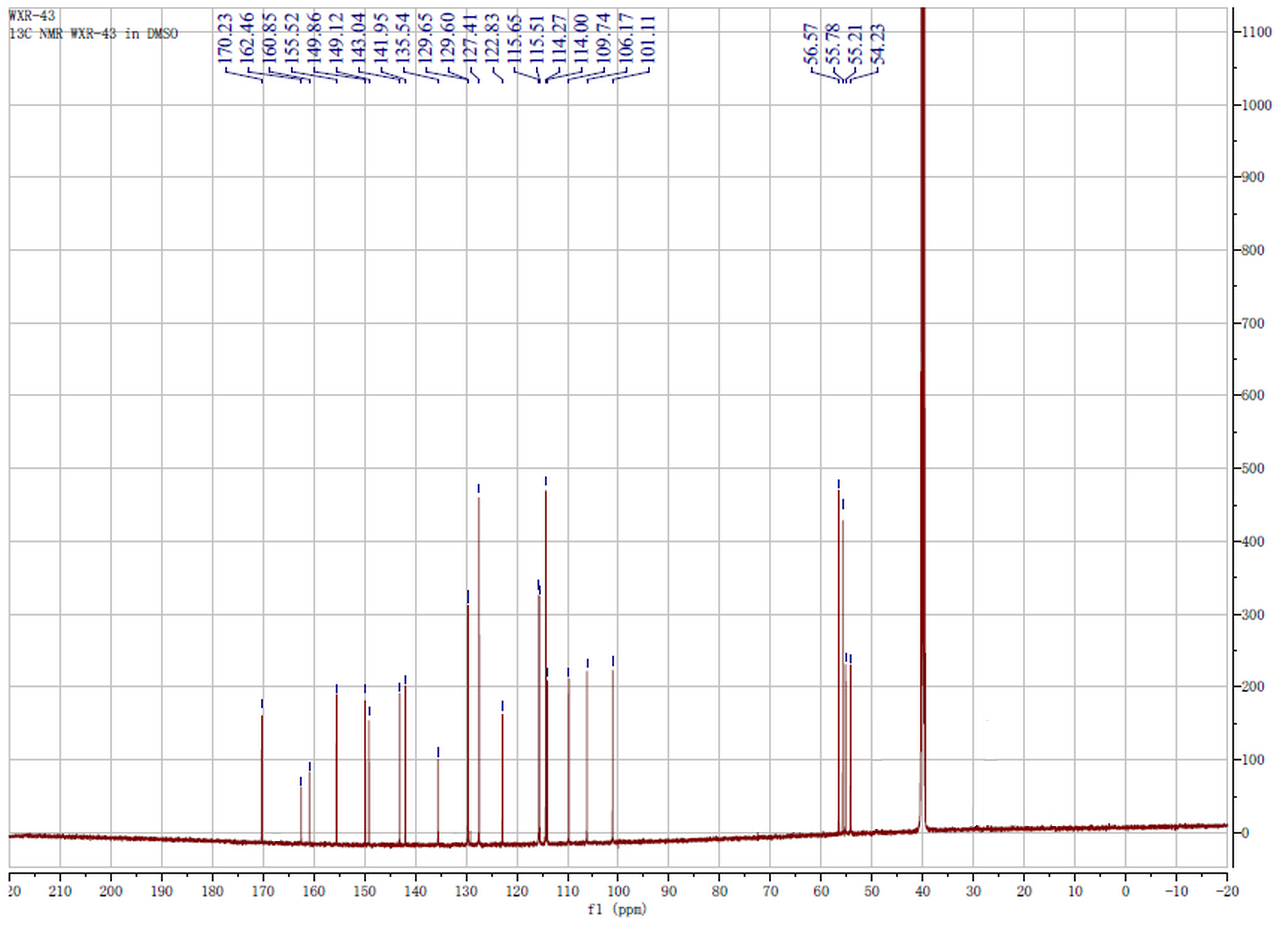

Supplement: Supplementary file 1 [file molecules-22-01925-s001.zip › Molecules-238010--13C NMR/44.png]

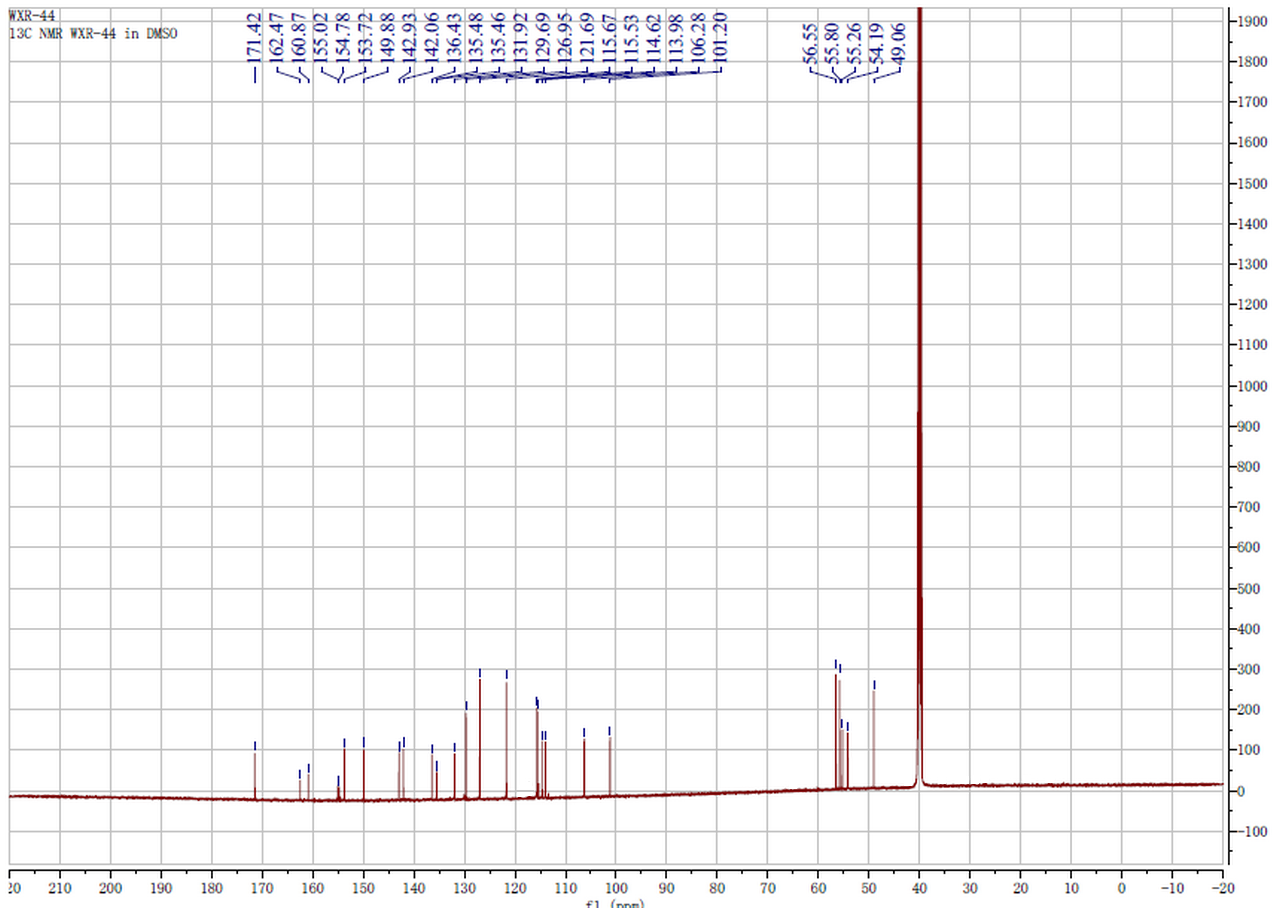

Supplement: Supplementary file 1 [file molecules-22-01925-s001.zip › Molecules-238010--13C NMR/45.png]

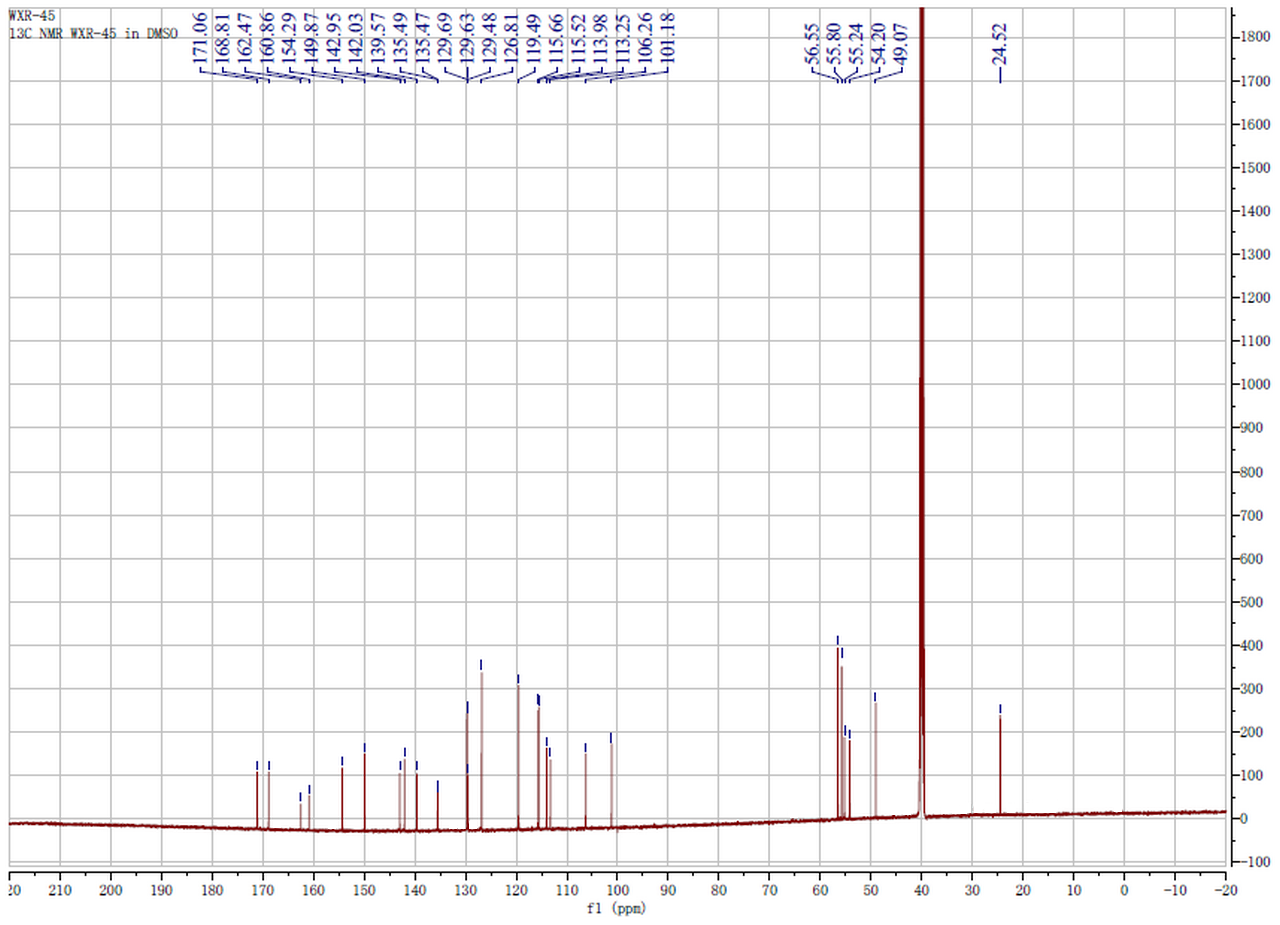

Supplement: Supplementary file 1 [file molecules-22-01925-s001.zip › Molecules-238010--13C NMR/46.png]

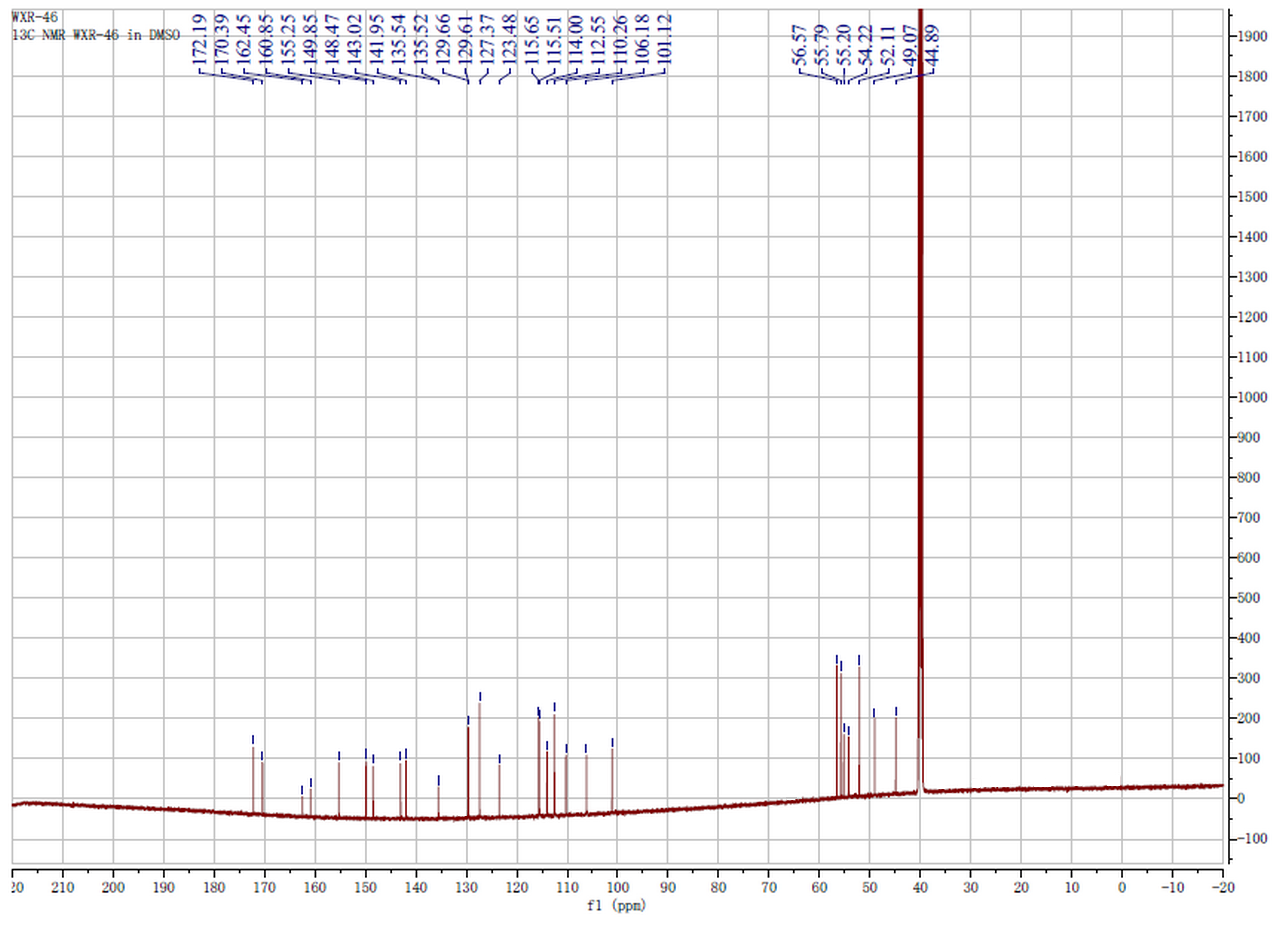

Supplement: Supplementary file 1 [file molecules-22-01925-s001.zip › Molecules-238010--13C NMR/47.png]

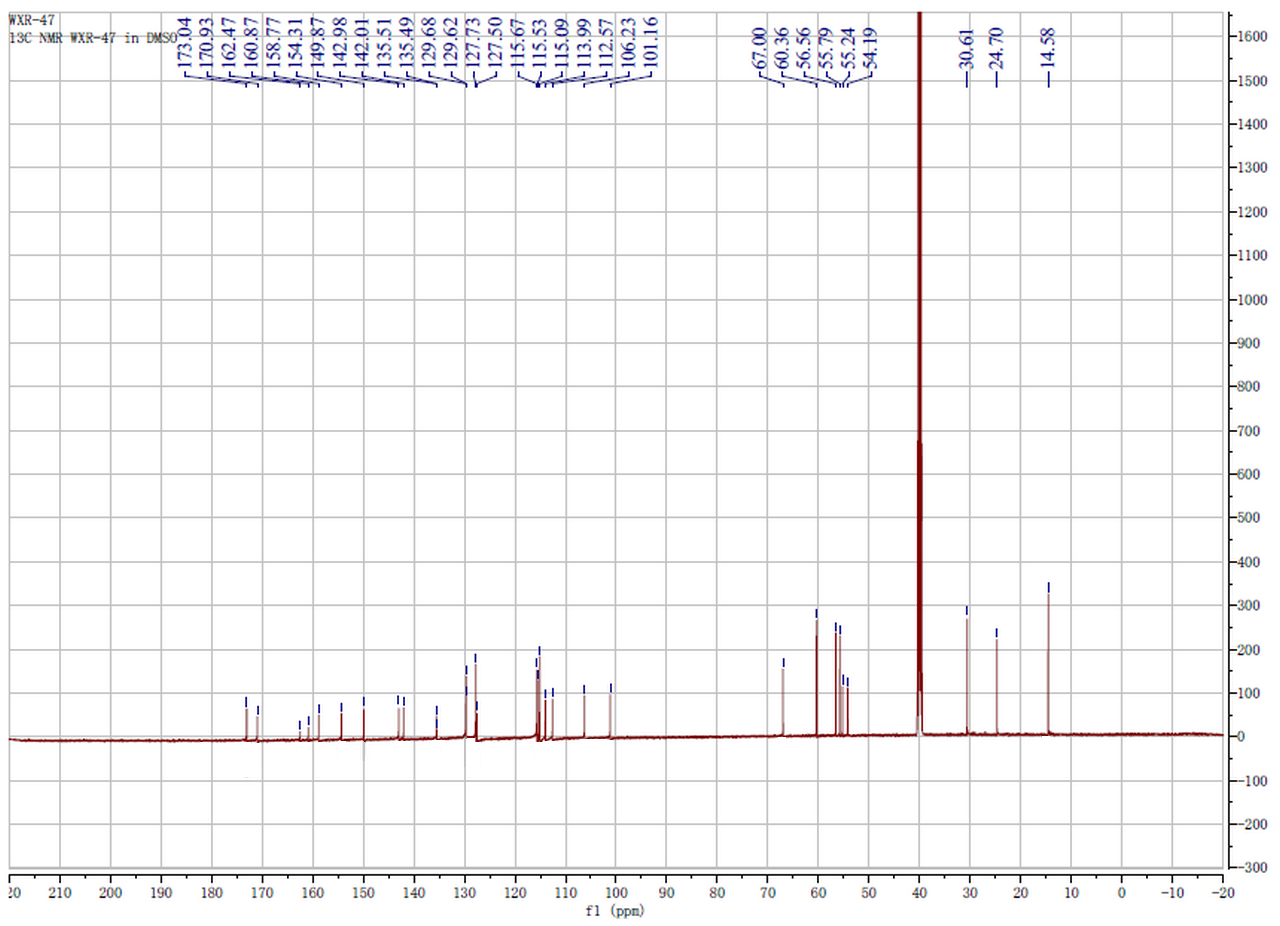

Supplement: Supplementary file 1 [file molecules-22-01925-s001.zip › Molecules-238010--13C NMR/48.png]

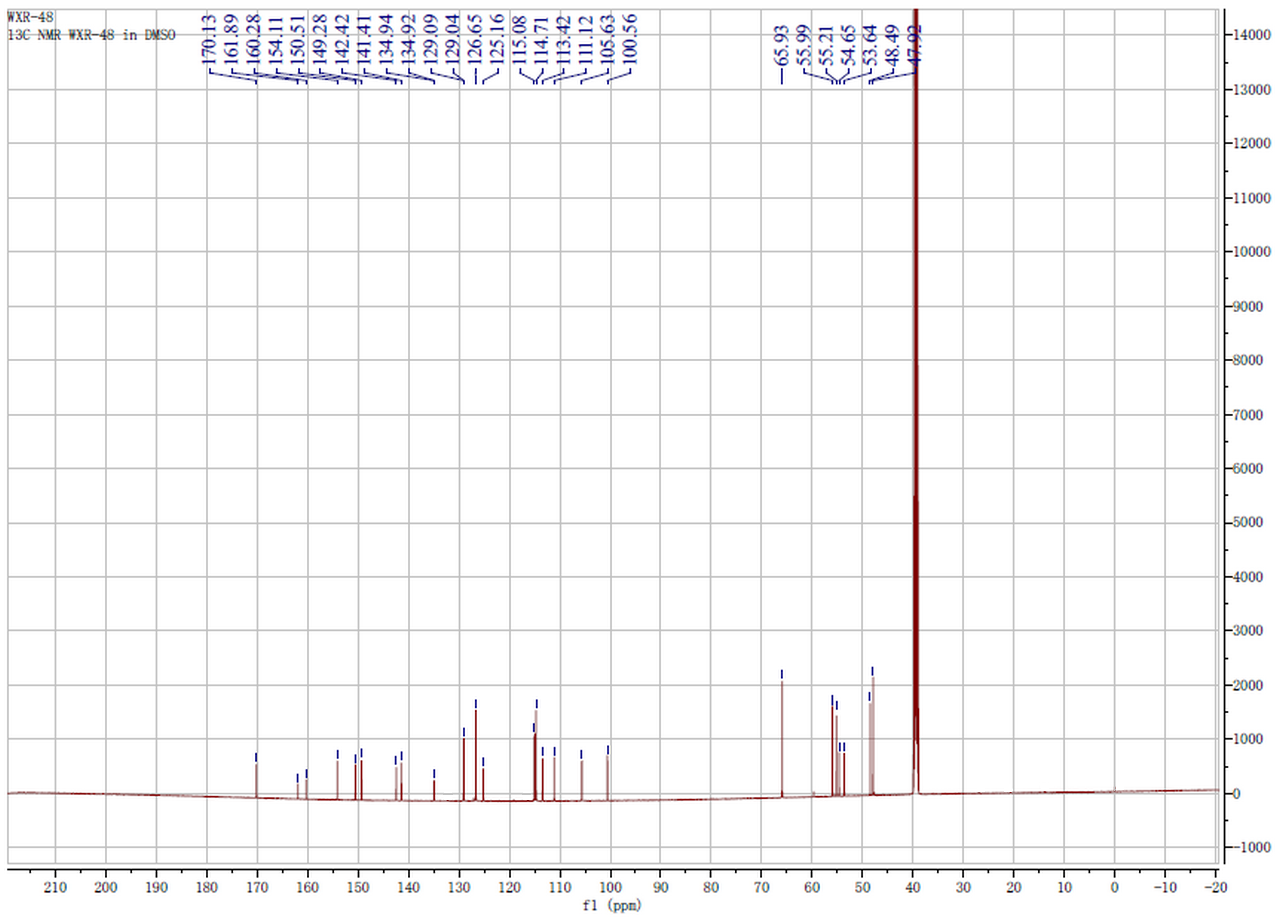

Supplement: Supplementary file 1 [file molecules-22-01925-s001.zip › Molecules-238010--13C NMR/49.png]
